# Yeast Biological Networks Unfold the Interplay of Antioxidants, Genome and Phenotype, and Reveal a Novel Regulator of the Oxidative Stress Response

**DOI:** 10.1371/journal.pone.0013606

**Published:** 2010-10-25

**Authors:** Jose M. Otero, Manos A. Papadakis, D. B. R. K. Gupta Udatha, Jens Nielsen, Gianni Panagiotou

**Affiliations:** 1 Department of Systems Biology, Center for Microbial Biotechnology, Technical University of Denmark, Lyngby, Denmark; 2 Department of Systems Biology, Center for Biological Sequence Analysis, Technical University of Denmark, Lyngby, Denmark; 3 Department of Chemical and Biological Engineering, Chalmers University of Technology, Gothenburg, Sweden; Georgia Institute of Technology, United States of America

## Abstract

**Background:**

Identifying causative biological networks associated with relevant phenotypes is essential in the field of systems biology. We used ferulic acid (FA) as a model antioxidant to characterize the global expression programs triggered by this small molecule and decipher the transcriptional network controlling the phenotypic adaptation of the yeast *Saccharomyces cerevisiae*.

**Methodology/Principal Findings:**

By employing a strict cut off value during gene expression data analysis, 106 genes were found to be involved in the cell response to FA, independent of aerobic or anaerobic conditions. Network analysis of the system guided us to a key target node, the FMP43 protein, that when deleted resulted in marked acceleration of cellular growth (∼15% in both minimal and rich media). To extend our findings to human cells and identify proteins that could serve as drug targets, we replaced the yeast FMP43 protein with its human ortholog BRP44 in the genetic background of the yeast strain Δ*fmp43*. The conservation of the two proteins was phenotypically evident, with BRP44 restoring the normal specific growth rate of the wild type. We also applied homology modeling to predict the 3D structure of the FMP43 and BRP44 proteins. The binding sites in the homology models of FMP43 and BRP44 were computationally predicted, and further docking studies were performed using FA as the ligand. The docking studies demonstrated the affinity of FA towards both FMP43 and BRP44.

**Conclusions:**

This study proposes a hypothesis on the mechanisms yeast employs to respond to antioxidant molecules, while demonstrating how phenome and metabolome yeast data can serve as biomarkers for nutraceutical discovery and development. Additionally, we provide evidence for a putative therapeutic target, revealed by replacing the FMP43 protein with its human ortholog BRP44, a brain protein, and functionally characterizing the relevant mutant strain.

## Introduction

Practically all cellular processes, including every step in the flow of genetic information from gene expression to protein synthesis and degradation, can be affected by lifestyle and dietary habits. Human disorders and nutrient metabolic responses suggest features whose complexities are defined by interactions among genes, and between genes and environmental stimuli. These interactions are often amplified and modulated through regulatory, protein, metabolic and signaling networks. However, our understanding on the nutrient-related network characteristics and function is still limited. Exploiting roles of nutritional compounds for the rational design of strategies to beneficially manipulate cell functions and/or cell fates is highly restricted by this lack of information. Network biology provides the opportunity to gain insight into the structure and function of biological systems, and a practical scaffold to bridge the gap between reductionistic and systems biology [1]. More complete knowledge of network function further enhances our ability to predict quantitative or qualitative relationships between specific health outcomes and diverse patterns or levels of nutrient intake in genetically diverse individuals and populations [2], [3].

A fruitful strategy to explore the field of nutritional research is to use well-established methods applied in medical and pharmacological research [4]. For example, in analogy to pharmacology, nutrients can be considered as signaling molecules recognized by specific cellular sensing mechanisms. However, in nutritional studies the system response is uniquely confounded by the simultaneous presence of many signal inputs, or in this case, nutrients with diverse chemical structures that have numerous targets with different affinities and specificities.

**Figure 1:**
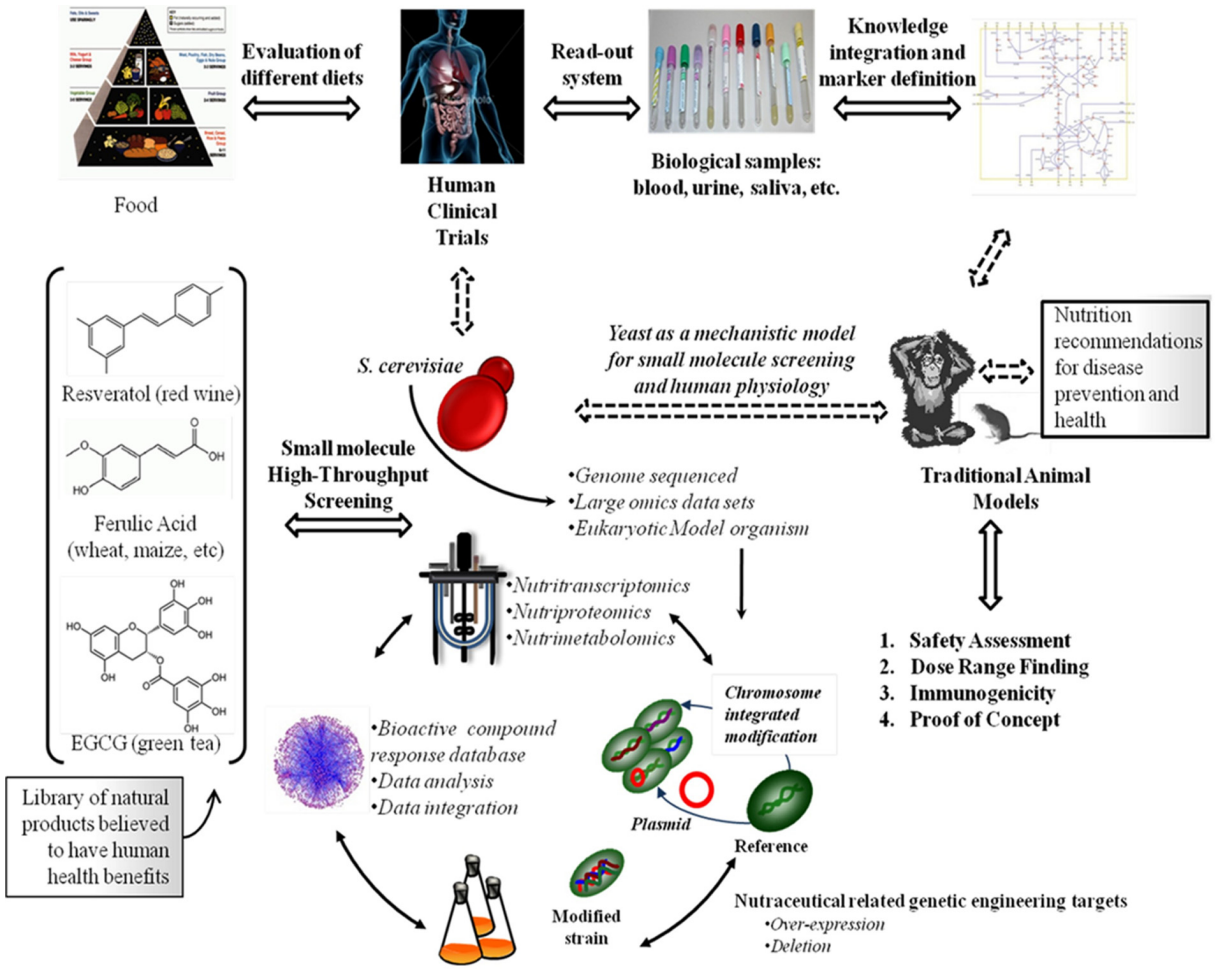
Application of bottom-up and top-down systems biology approaches for the development of functional foods that target specific body functions through either individual ingredients (e.g., resveratrol, ferulic acid, epigallocatechin gallate, EGCG) or specially designed mixtures (e.g., food ingestion). A discovery process that derives molecular markers for the bioactivity of defined foods through a human intervention trial, and the use of pathway models (bottom-up) should be followed by an animal model study to verify the markers *in vivo*. Similarly, the biomarker signature identification of specific nutrients using nutri-omics technology in yeast (top-down) will eventually be tested by an intervention study in an animal model. The output of this process, which might well be iterative, is new knowledge obtained for the biological system, as well as the potential for predictive understanding of that system; in the nutritional arena this would lead to personalized nutrition.

A logical approach to successfully overcome the layered complexity of nutritional research is to dissect, reduce, and classify the challenges. This approach allows the definition of specific hypotheses that can be evaluated using an appropriate model system (Figure 1), and thus obtain clear answers to the research question being addressed [5], [6]. The current enthusiasm for antioxidants is perhaps of no surprise, as studies suggesting health benefits from these compounds continue to attract main stream headlines [7]–[9]. Yet, even though antioxidants have been studied for the last 60 years, much about how the human body absorbs and utilizes such compounds remains unknown. Often, products boasting health benefits are largely unsubstantiated in their scientific claims and mechanism of action. Ferulic acid (FA) is unique among the plant phenolic compounds being a dietary supplement exhibiting the highest bioavailability among all flavonoid and monophenolics tested until today. By virtue of its high scavenging activity against free radicals, its potent membrane antioxidant properties, and its ability to inhibit enzymes that catalyze the production of free radicals, FA could as a nutraceutical play a role in the pre-disease state, either for improving human health, or for preventing disease.

The importance of FA as a nutraceutical or pharmaceutical agent against diverse human disorders has been extensively evaluated. First of all, FA exhibits strong activity against microbes, including many bacteria and viruses. Secondly, FA and its ester derivatives decrease the levels of some inflammatory mediators, such as TNF-α [10]. In addition, FA has been proven beneficial against cardiovascular diseases, as it decreases the levels of the very low density (VLDL) and low density (LDL) lipoproteins and increases the levels of the high-density lipoprotein (HDL) cholesterol in plasma. Moreover, FA derivatives not only inhibit collagen-induced platelet aggregation, which is closely associated with thrombosis, but they are also capable of dissolving thrombi [11]. Furthermore, FA shows anti-diabetic effects by neutralizing the free radicals present in the pancreas, and thus helps the beta cells to proliferate and secrete more insulin. In return, elevated insulin levels increase glucose utilization by the extra hepatic tissues, resulting in lower blood glucose concentration levels [12]. Polyphenols, including FA, reduce proliferative activity and induce apoptosis in a variety of tumor cells [13]. Finally, a recent study on rats revealed that orally administrated FA enhances the proliferation of adult neural stem/progenitor cells *in vivo*. The same study suggested a potential anti-depression effect of FA in mice [14].

Conversely, plant phenolic acids are potent inhibitors of microorganisms and provide a natural protection against pathogenic infections. Diverse biotechnological or industrial processes accomplished by *S. cerevisiae* are affected by such inhibitory properties. The inhibitory effect of FA and other phenolic compounds involved in the diverse FA degradation pathways (e.g. vanillin, coniferyl alcohol, eugenol etc.) on yeast cultures has recently been confirmed. Specifically, the biomass yield and growth rate, but not ethanol yield, are highly affected by the presence of FA and its related compounds [15]. Interestingly, *S. cerevisiae* has been shown to convert FA into vinyl guaiacol (4-hydroxy-3-methoxy styrene) in 96% yield and diFA (4-hydroxy-3-methoxy phenylpropionic acid) in 54% yield under an atmosphere of argon [16].

A new biomarker concept to predict human health effects of nutrients and develop nutraceuticals based on systems and network biology is presented here. We characterize the pathway responses of *S. cerevisiae* upon defined perturbations: controlled environmental stimuli using an antioxidant model compound, deletion of a gene –whose protein product constitutes a significant node of the network architecture– and insertion of its human ortholog, and we assess their interaction by integrating such information into graphical network models, which elucidate predictive hypotheses to explain emergent behaviors.

## Materials and Methods

### Strains and media

The haploid, prototrophic *S. cerevisiae* strain CEN.PK 113-5D (*MAT* **a** *SUC2 MAL2*-*8* ^c^ *ura3*-52) was used in all cultivations. Cultures were maintained by plating in YPD medium and such stocks were used to inoculate the pre-cultures. Pre-cultures were prepared in shake flasks using defined mineral medium [17], supplemented with vitamins, adjusted to pH 6.0 and containing 2% (w/v) glucose. For anaerobic cultivations, the medium was supplemented with Tween 80 and ergosterol. When needed, FA was added with sterile filtration in a final concentration of 0.8 g/L.

### Construction of the yeast strain

#### CEN.PK 113-5D/Δ*fmp43*

The strain CEN.PK 113-5D with the single *FMP43* deletion was created using a two-step gene deletion strategy [18]. The upstream and downstream regions of *FMP43* were amplified using the genomic DNA of strain CEN.PK 113-5D as a template. *Kluyveromyces lactis URA3* was amplified as two overlapping fragments−referred to as upstream and downstream− using two primer pairs and the plasmid pWJ1042 as a template. The amplified upstream fragment of *FMP43* was fused to the upstream fragment of *URA3*, and the *FMP43* downstream fragment was fused to the *URA3* downstream fragment. These two fusion fragments were used as transformation material for the strain CEN.PK 113-5D. A total of 10 Ura^+^ transformants −grown on synthetic complete (SC) Ura^−^ medium− were streak-purified and re-streaked on SC plates containing 5-fluoroorotic acid (5-FOA, Zymo Research) to selectively loop out the *K. lactis* gene. The 5-FOA-resistant colonies were picked out and checked for loss of *URA3* by replica plating on SC-Ura^−^ plates. The deletion of *FMP43* was confirmed by PCR (see Table S8).

### Construction of the yeast strain

#### CEN.PK 113-5D/Δ*fmp43*/pRS426-*BRP44*

The synthetic, codon-optimized *BRP44* expressed on pUC19 was purchased from Codon Devices, Inc. Restriction endonucleases, enzyme buffers (NEB) and bovine serum albumin (BSA) were purchased from New England Biolabs, Inc. The frame for cloning of the *BRP44* fragment, i.e pRS426, was a vector kindly provided by Dr S. Wattanachaisaereekul. The extraction of the pRS426 or pRS426-*BRP44* DNA from *E. coli* DH5α cells was achieved using the GenElute Plasmid Miniprep Kit from Sigma-Aldrich, Co. For the purification steps, the QIAEX II Gel Extraction Kit from Qiagen was used.

Liquid cultures of *E. coli* DH5α cells used during cloning were prepared in 5 mL lysogeny broth (LB) in sterile 15 mL tubes and left to shake at 150 rpm overnight at 37°C. The medium contained 10 g tryptone, 5 g yeast extract, and 10 g NaCl pr. liter, taken to pH equal to 7.0 with NaOH prior to autoclavation. The ligation product was transformed into strain CEN.PK 113-5D/Δ*fmp43* to over-express *BRP44*, and the Ura^+^ phenotype was selected on SC-Ura^−^ plates.

### Batch and chemostat cultivation conditions

To determine the physiological characteristics of the different yeast strains in the presence and absence of FA, the *S. cerevisiae* control and recombinant strains were grown in batch cultivations in well-controlled 2 L bioreactors with a working volume of 1.5 L. The chemostat cultivations (at 0.1 h^−1^dilution rate) were performed in the same advanced bioreactors with 1 L working volume. The bioreactors were provided with the defined mineral medium described above, containing glucose (2% w/v) as the limited nutrient. The bioreactors were equipped with two disc-turbine impellers rotating at 600 rpm. The pH was kept constant at 5.0 by addition of 2 M KOH or HCl, and the temperature was maintained at 30°C. Air or nitrogen was used for sparging the bioreactor at a constant flow rate of 1.0 vvm (volume of gas per volume of liquid per minute).

### Analysis of substrates and products

Cell dry weight was determined using nitrocellulose filters (pore size 0.45 µm, Gelman Sciences). Fermentation samples were immediately filtered and stored at −20°C until analysis. The concentrations of glucose, ethanol, glycerol, acetate, succinate, pyruvate and FA were determined by HPLC as previously described [19], [20]. Yields and production/consumption rates were calculated in C-moles.

### Sampling, extraction, determination and analysis of intracellular and extracellular intermediary metabolites

For the analysis of intracellular and extracellular metabolites, triplicate biological samples were collected and immediately quenched in cold 72% v/v methanol (−40°C) as previously described [21]. The lyophilized samples were derivatized using methyl chloroformate, and amino and non-amino organic acids were analyzed by GC-MS [22]. GC-MS analysis was performed with a Hewlett-Packard system HP 6890 gas chromatograph coupled to a HP 5973 quadrupole mass selective detector (EI) operated at 70eV. The profile of identified intracellular and extracellular amino and non-amino organic acids was expressed in peak areas normalized by the mass of biomass.

### DNA microarrays; Harvesting, RNA extraction and microarray hybridization

Biomass samples were collected rapidly to limit any potential changes to the transcriptional profiles of the strains. Forty mL of culture broth was sampled directly from the bioreactor into a 50 mL centrifuge tube containing crushed ice. After vigorous stirring, the sample froze instantly. The cells were pelleted (5000 rpm at 0°C for 4 min), and RNA was extracted using the hot-phenol method [23]. RNA quality was checked using the Agilent 2100 Bioanalyzer. Fifteen µg of fragmented biotin-labeled cRNA was prepared from 5 µg of total RNA and hybridized to the YEAST 2.0 Affymetrix GeneChip according to the Affymetrix GeneChip expression analysis technical manual [24].

### Analysis of transcriptome data

The differential gene transcription caused by the presence of FA under aerobic and anaerobic chemostat cultivations (D = 0.1 h^−1^) was examined. The scanned probe array images (.DAT files) were converted into CEL files using the default values of the GeneChip operating software (version 1.4) from Affymetrix. The probe-level data in CEL files were subsequently processed using the statistical open source language R (version 2.5) [25]. Data pre-processing was carried out using the robust multichip average (RMA) method [26], which is available in the affy package [27]. This package implements RMA by correcting the perfect match (PM) probes, performing quantile normalization [28] and calculating the expression measure by using median polish.

The effects of FA were examined by 2×2 ANOVA statistical analysis of different subgroups (sg); sg1:(+)O_2_(±FA) vs. sg2:(−)O_2_(±)FA vs. sg3:(±)O_2_(-FA) vs. sg4:(±)O_2_(+FA), to identify significantly differentially expressed genes being FA-responsive, independent of oxygen supply. Statistical analysis to determine genes subject to differential transcription regulation was performed using the limma package [29]. Moderated t-tests were used for the comparison of FA effect on yeast cells. For all comparisons, we used empirical Bayesian statistics to moderate standard errors within each gene and the Benjamini-Hochberg's method to adjust p-values for multiple testing [30]. To determine statistically significant gene expression changes, the logarithmic fold change was higher than 1 and the highest cut off value for the calculated adjusted p-values was set at 0.001.

### Bioinformatic tools and software

Cytoscape (version 2.5.2) [31] was used for the PPI network analysis, where several plug-ins were applied to the resulted sub-networks: Molecular COmplex DEtection (MCODE, version 1.3), Biological Networks Gene Ontology (BiNGO, version 2.0), jActiveModules (version 2.23), and cell region-based rendering and layout (Cerebral, version 1.2). The MCODE algorithm was applied by using the following parameters: for network scoring, a degree cutoff of 2 was chosen, while loops were not included. For cluster finding, the node score cut off was 0.2, haircuts (but not fluffs) were considered, k-core was equal to 2, and the maximum depth from seed was set to 100. During BiNGO application for the study of biological process (BP) enrichment in the data, the hypergeometric statistical test was chosen, while for multiple testing correction the Benjamini-Hochberg false discovery rate (FDR) method with a significance threshold of 0.05 was applied. To derive the corresponding functional enrichment, each protein complex or selected network region was tested versus the whole available set of genome annotation. The GENECODIS tool [32] was also used for the gene ontology analysis of the ACTMOD network, and specifically for the cellular component (CC) enrichment analysis of the latter. During GENECODIS application, the hypergeometric statistical test without p-value correction was chosen to retrieve CC term enrichments at the lowest level.

During the study of reporter features resulting from the transcriptional profiles of the yeast strains Δ*fmp43* and WT, the REPORTER FEATURE software (version 5.0) was used [33]. Also, diverse databases were explored throughout the present work (data downloaded in September 2009): eukarYOtic ortholoGY (YOGY, www.sanger.ac.uk/PostGenomics/S_pombe/YOGY), *Saccharomyces* Genome Database (SGD, www.yeastgenome.org), YEASTRACT (www.yeastract.com), PubMed (www.ncbi.nlm.nih.gov), BLAST (www.ncbi.nlm.nih.gov/blast/Blast.cgi), and UniProt (www.uniprot.org). Predictions for the FMP43 protein were performed using multiple servers available at the Center for Biological Sequence analysis (CBS, Kgs. Lyngby, Denmark): ProtFun (version 2.2), TMHMM (version 2.0), NetNES (version 1.1), SignalP (version 3.0), TargetP (version 1.1), NetPhosYeast (version 1.0), NetAcet (version 1.0) and YinOYang (version 1.2).

*Homology modeling and structure validation*: Sequences of PDB structures showing similarity with the BRP44 and FMP43 amino acid sequences were retrieved from the RCSB Protein Data Bank using different cut offs for E-values. Secondary structure analysis of the BRP44, FMP43 and template sequences was accomplished using the SOPMA secondary structure prediction method. Homology modeling of both BRP44 and FMP43 proteins was performed using the Swiss-PdbViewer. The ProSA-web protein structure analysis tool was used to validate the overall quality of structural models obtained from homology modeling. The compatibility of atomic model (3D) with its own amino acid sequence was analyzed using the Verify3D structure evaluation server. The stereo-chemical quality of each protein structure was checked using the Procheck program. We used the Protein Structure Validation software suite to determine statistical parameters of structure quality factors.

### Prediction of protein-ligand binding sites

Protein-ligand binding sites in BRP44, FMP43, FAEA (*Aspergillus niger*) and FAE domain of xyn10B (*Clostridium thermocellum*) were predicted using the Q-SiteFinder tool. Q-SiteFinder works by binding hydrophobic (CH_3_) probes to the protein, and then finding clusters of probes with the most favorable binding energy. These clusters are ranked according to the sum of total binding energies, indicating the likelihood of each cluster to be a binding site.

### Molecular Docking

Chemical Entities of Biological Interest (ChEBI), a freely available dictionary of molecular entities, was used to retrieve FA in the Mol format. Protein-ligand docking studies using Lamarckian genetic algorithm scoring functions to find the low-energy binding mode were employed in the ArgusLab software.

## Results

### Phenotypic biomarkers in S. cerevisiae cultivations reveal the presence of an antioxidant molecule

The *S. cerevisiae* CEN.PK 113-5D (CON) was grown at aerobic and anaerobic conditions in well- controlled bioreactors in the presence and absence of FA using defined medium with glucose as the sole carbon source. CON was capable of growing aerobically and anaerobically in the presence of FA at a specific growth rate (μ_max_) of 0.23 h^−1^and 0.15 h^−1^, respectively. This result shows the paramount effects of FA −when added in deleterious concentrations− on the physiology of yeast cells and in particular on growth, since µ_max_ was decreased by 32% and 46% (with and without oxygen supply, respectively) compared with the cultivations without FA (Table 1).

**Table 1:** Physiological characterization of S. cerevisiae (CON), with and without perturbation by an antioxidant compound (FA), under aerobic and anaerobic conditions.

		Aerobic Cultivation Conditions		Anaerobic Cultivation Conditions	
		(-)FA	(+)FA	(-)FA	(+)FA
	µ_max,_ h^−1^	0.34	0.23	0.29	0.16
Specific _Production/Consumption Rates_		_C-_mmol/g-DCW/h		C-mmol/g-DCW/h	
	Glucose	100.21	80.29	93.11	66.42
	Biomass	10.69	6.85	12.48	5.61
	Ethanol	44.28	34.27	41.03	25.95
	Acetate	0.17	0.23	0.36	0.41
	Glycerol	5.53	4.81	10.46	7.71
	Pyruvate	0.37	0.16	0.33	0.13
	CO_2_	11.57	7.21	11.06	4.97
	O_2_	8.04	2.09	nd	nd

Yields		C-mol/C-mol glucose		C-mol/C-mol glucose	
	Biomass	0.107	0.085	0.134	0.084
	Ethanol	0.442	0.427	0.441	0.391
	Acetate	0.002	0.003	0.004	0.006
	Glycerol	0.055	0.061	0.112	0.116
	Pyruvate	0.004	0.002	0.004	0.002
	^CO^_2_	0.115	0.091	0.119	0.075
	O_2_	0.08	0.026	Nd	Nd

All values were calculated in batch culture (biological triplicates) during exponential growth phase on glucose, and identified by the linear relationship between the natural logarithm of biomass and the culture time.

Under aerobic conditions, the presence of FA reduced markedly the glucose consumption rate, r_s_, as well as the biomass production rate, r_x_. However the most devastating effect of FA was on the ability of the cells to respire. In the presence of FA, the determined maximum specific consumption rate of oxygen was 2.1 mmol/g-DW/h, which corresponds to a 4-fold decrease when compared with the condition where no FA was supplemented (Table 1). The yield and productivity of acetate and ethanol, which constitute two common metabolites in yeast metabolism −both originating from pyruvate with different redox cofactors involved in their pathways− were also affected by the addition of FA. In both aerobic and anaerobic cultivations, acetate level was increased by FA supplementation, while the opposite trend was observed for ethanol. Furthermore, at aerobic conditions and in the presence of FA, yeast showed lower respiratory capacity resulting in inability to consume ethanol after glucose depletion (data not shown). In addition, under anaerobic cultivations and in the presence of FA, the carbon evolution rate (CER) was represented by one wide peak (data not shown), reaching the maximum value when the glucose concentration in the medium was 8–9 g/L. This indicates that the impact of FA on yeast is extended to mechanisms other than simply respiratory metabolism.

### Reflection of the antioxidant activity of FA on the metabolome of the yeast cell

To further evaluate the effect of FA glucose-limited chemostat experiments were performed at a dilution rate of D = 0.1 h^−1^ under both aerobic and anaerobic conditions and in the presence and absence of FA. This experimental set-up facilitates the direct measurement of the impact of FA on the endo-metabolome of the yeast *S. cerevisiae* avoiding growth-rate associated trends. All carbon-containing substrates can be oxidized to CO_2_, and relative to this end product of biochemical reactions, the substrates and the carbon-containing metabolic products are in the reduced state. The degree of reduction (DOR) is a systematic way of defining the redox level of different chemical compounds. A higher value of the DOR indicates a less oxidized compound.

To evaluate the applicability of the *S. cerevisiae* platform for antioxidant discovery, the oxidative state of the 45 measured amino and non-amino organic acids was calculated using the DOR of each metabolite (Table S1). The value for the metabolite with the highest DOR was arbitrarily assigned to 1, and all the other metabolites were accordingly linearly scaled. The ratio of the concentration of each intracellular metabolite in the presence versus absence of FA was plotted against the DOR of the metabolite. By comparing the level of the intracellular metabolites under aerobic conditions, with and without FA, we observed that the presence of FA increased the concentration of metabolites with DOR higher than the average value (0.77) and decreased the concentration of metabolites with DOR values lower than the average, supporting the compound's antioxidant potential. The exact same trend was observed for the metabolites that had higher concentrations under anaerobic conditions and when FA was present. However, the metabolites being at higher levels during anaerobic and without FA cultivations were more scattered around the average DOR, showing no clear trend that could be related to the oxidative status of the cell.

### Uncovering the complexity of the yeast network response evoked by an antioxidant

**Figure 2:**
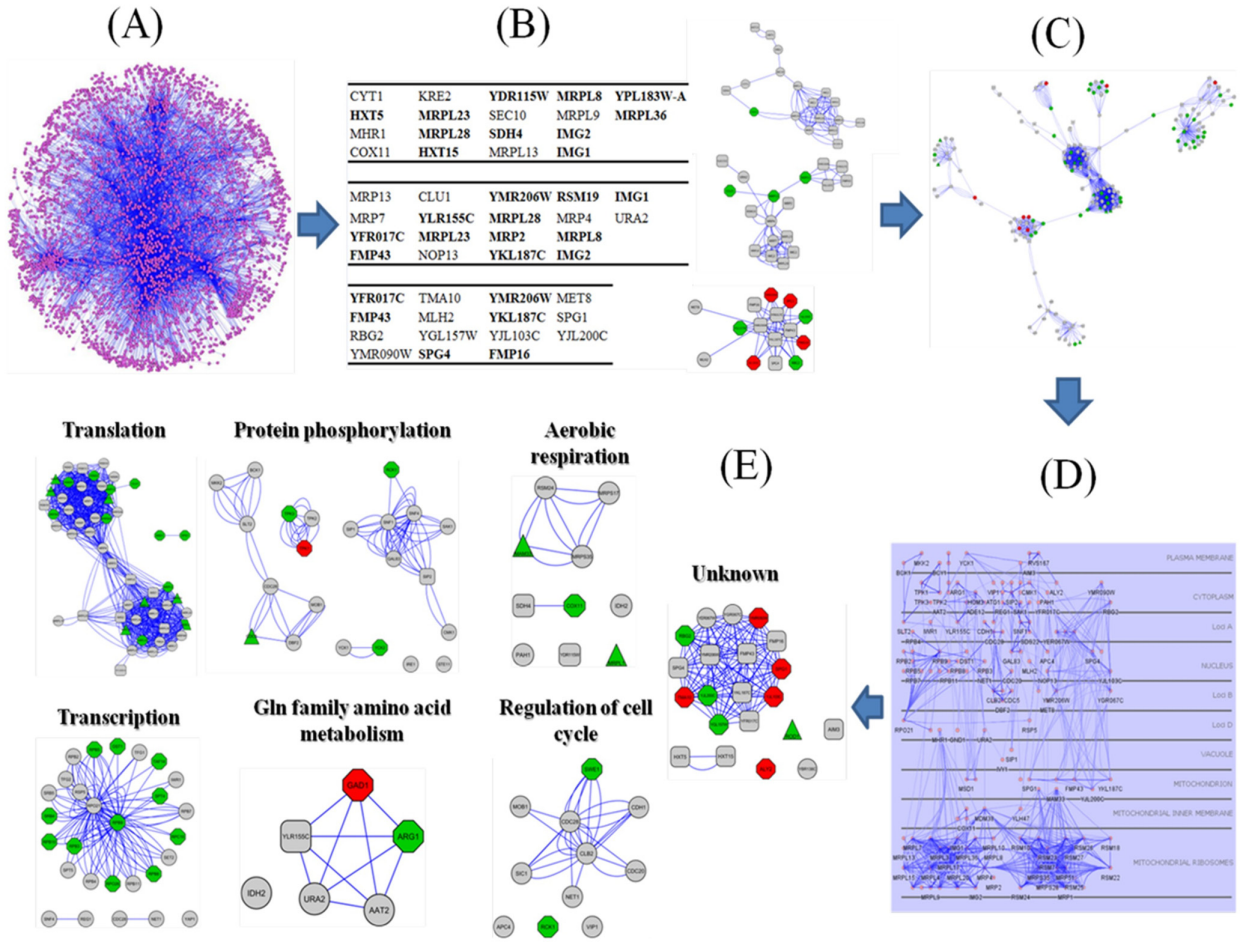
Understanding the dynamic programs that a yeast cell utilizes in response to the external stimulus of an antioxidant compound. (**A**) The primary FA-specific protein-protein interaction (PPI) network (3,251 nodes, 12,462 edges) constructed by the list of the 64 genes that significantly responded to the FA environmental perturbation, independently of the aeration level, (**B**) High-scoring active modules identified in the primary FA-specific PPI network. Bold font indicates genes that belong to the list of the 64 genes, (**C**) The ACTMOD network, consisting of 167 nodes and 1,651 edges, using the “organic layout” in Cytoscape, (**D**) Sub-cellular localization of the 167 nodes of the ACTMOD network, (**E**) Functional enrichment (biological process, BP) of the ACTMOD network. For some nodes no functional annotation could be retrieved. During visualization feature assignment, color and shape codes have been defined as follows. *Shape code*: OCTAGON was used to indicate genes with significant differential expression in the (+)FA(+)O_2_ vs. (+)FA(−)O_2_ t-test, TRIANGLE for genes with significant differential expression in the (+)FA(−)O_2_ vs. (−)FA(−)O_2_ t-test, ROUND RECTANGULAR for genes with significant differential expression in the ANOVA statistic, and PARALLELOGRAM for genes with significant differential expression in the (+)FA(+)O_2_ vs. (−)FA(+)O_2_ and (+)FA(−)O_2_ vs. (−)FA(−)O_2_ t-tests. *Color code*: GREEN indicates down-regulation, while RED indicates up-regulation.

By employing a strict cut off value (*p_adjusted_*<0.001), 106 genes were found to be involved in the cell response triggered by FA, independent of aerobic or anaerobic conditions (Table S2). The list was further reduced for subsequent analysis by removing genes that appear not to have a human ortholog −with the aim to propose conserved mechanisms used from yeast to humans. The remaining 73 genes were submitted to the SGD and STRING databases (Table S3) to retrieve high quality protein-protein interactions (PPIs). However, data could be obtained for only 64 of the 73 genes. The files were imported into the Cytoscape software and the resulted FA-specific network, consisting of 3,251 nodes and 12,462 edges, is displayed in Figure 2A.

To find densely connected regions in the molecular interaction network, MCODE, a density-based local search algorithm, was applied. The predicted protein complexes contained several known molecular complexes, but the majority of genes that were both functionally connected and present in the original gene list (64 genes) corresponded mainly to procedures, like translation, regulation of progression through the cell cycle, transcription, aerobic respiration and protein phosphorylation (Table S4).

To identify causative networks associated with the observed phenotypic response to the FA-stimulus, we integrated the PPI data with the expression data. Due to the fact that topologically interesting nodes tend to represent key regulatory elements of signaling cascades, while differentially expressed genes are likely to be found among target genes whose expression is regulated by these cascades, a combined analysis could provide new insights in the molecular mechanisms underlying the antioxidant activity of small molecules. We identified 3 highly active sub-networks (*Z*-scores ranging from 6.6 to 7.2, with scores higher than 3 considered significant), whose members are shown in Figure 2B. In the next analysis step, the two network concepts introduced by MCODE and jActiveModules algorithms were linked together by merging the corresponding algorithmic outputs in Cytoscape. The resulting network (174 nodes and 1,687 edges) consisted of four sub-networks: one large (149 nodes and 1,580 edges) and 3 smaller (12 nodes and 58 edges; 9 nodes and 33 edges; 4 nodes and 14 edges) sub-networks.

We further investigated the potential connectivity among these sub-networks. To do so, we extracted from the primary FA-specific network the first neighborhood of nodes, which appeared as significantly differentially expressed in the original gene list (64 genes), and then we imported the corresponding small networks in Cytoscape and checked their connectivity to the larger network. This procedure was followed separately for each sub-network. In this manner, we observed that the larger sub-network was interconnected to only one of the remaining three sub-networks, while the resulting network was named ACTMOD network (167 nodes and 1,651 edges) and is shown in Figure 2C.

Subsequently, the ACTMOD network was studied for node sub-cellular localization. As indicated in Figure 2D, nodes were localized in almost every cellular compartment, however, most of the nodes belonged to the yeast mitochondrion, where the majority of reactive oxygen species (ROS) species is generated. To understand the functional connectivity of the ACTMOD network, we determined the GO terms being significantly over-represented. The results were in agreement with those observed when the MCODE algorithm was applied to the primary FA-specific network, showing high confidence enrichment for translation (*p* = 5.51×10^−19^), protein amino acid phosphorylation (*p* = 1.22×10^−12^), aerobic respiration (*p* = 3.11×10^−03^), transcription (*p* = 1.48×10^−02^), amino acid metabolism (*p* = 1.61×10^−02^) and regulation of progression through the cell cycle (*p* = 4.80×10^−02^) (Figure 2E). It should be noted that functional annotation has not been assigned to all 167 nodes of the ACTMOD network, with the highly connected nodes of the FMP43-associated protein complex representing the majority of such nodes (Table S5).

### Transcriptional regulation leads to a novel gene-target of antioxidant components

**Figure 3:**
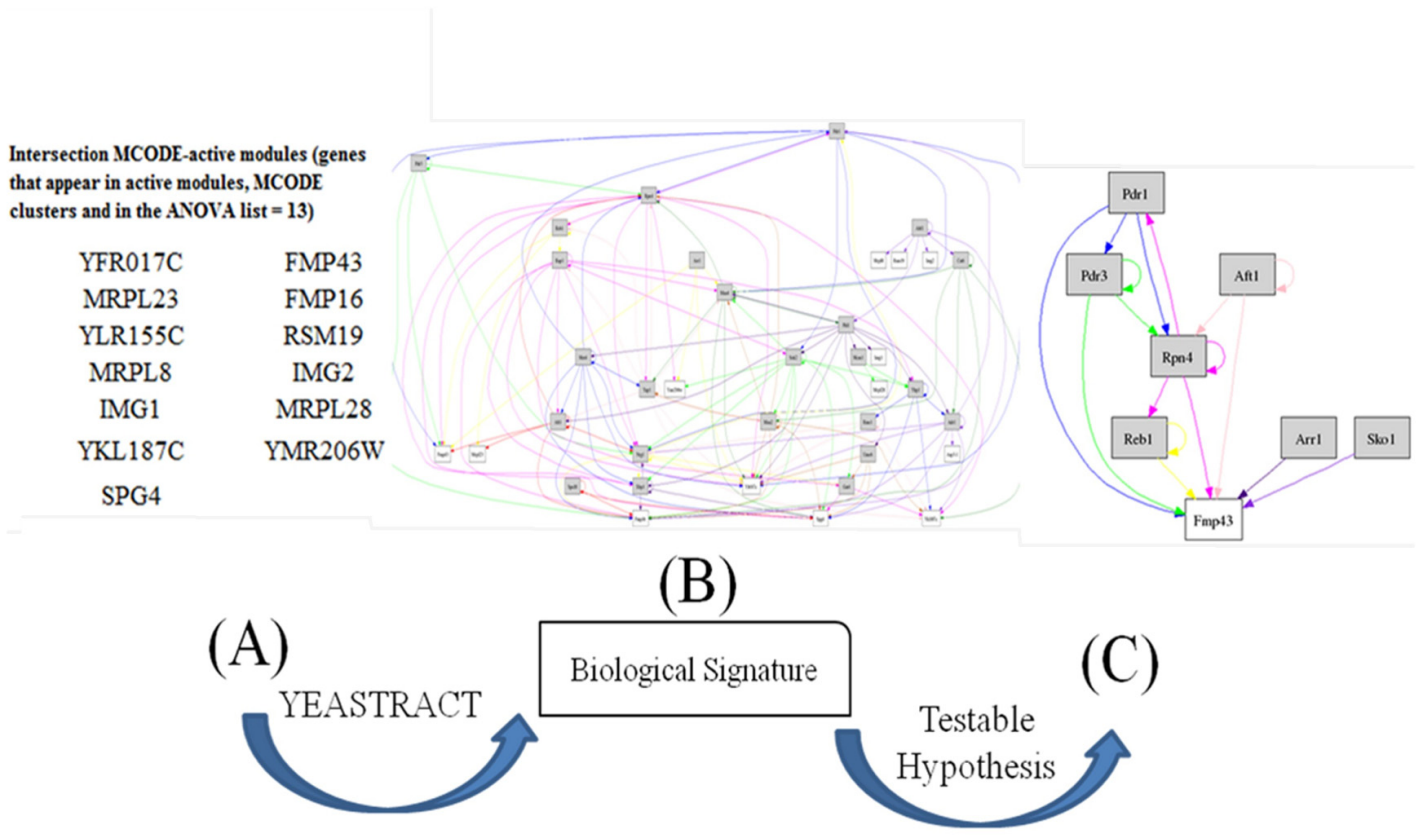
Integrating information from network connectivity and gene expression data. (**A**) The Table contains 13 genes that were present in our initial FA-specific gene list (ANOVA), and simultaneously in the significant MCODE clusters and the identified high-scoring active modules. (**B**) The transcriptional regulatory network (24 TFs) controlling the expression of the 13 genes. (**C**) A sub-network with the 7 TFs that regulate the expression of FMP43 protein. *Grey font*: Transcription factors, *White font*: Genes regulated by these TFs.

To be clinically relevant, results as those obtained above need to be translated and reduced to the level of a testable hypothesis about individual genes and proteins within the condition of interest. By integrating information from network connectivity and gene expression data, a list of 13 genes −present in the MCODE clusters, active modules and the ANOVA list of significant genes− was obtained (Figure 3A). We examined the transcriptional regulation of these set of genes, and as shown in Figure 3B, a very tight regulatory network controls the expression of all 13 genes with more than 20 transcription factors (TFs) being involved. A literature survey on these TFs revealed that a fraction of them has a crucial role in different stress responses in yeast (Msn4, Yap1, Xbr1, Msn2, and Gat4). A second set of transcriptional regulators is involved in the cell cycle progression (Rme1, Ume6, Sok2, Mcm1 and Yhp1), while the function of one TF (Adr1) is connected with the activation of genes involved in ethanol consumption, a phenotypic deficiency observed during our batch cultivations.

A third group of TFs was one of the main coordinators of the fine-tuning of the yeast response to FA (Figure 3C). Rpn4 encodes a transcription activator that induces the proteasome genes, and recent studies have led to a model in which the proteasome homeostasis is regulated by a negative feedback circuit, a mechanism that exists also in higher eukaryotes, including humans [34], [35]. In one of these studies, it was demonstrated that down-regulation of the proteasome genes, regulated by Rpn4, was able to reduce the active proteasome levels, a finding with potential clinical relevance in cancer cells [36]. Aft1 is another TF that controls the activation of 40% of the up-regulated genes in response to neurotoxicants [37]. Recent studies have elucidated the mechanisms of neural damage associated with these compounds and their linkage to the development of Parkinsonism symptoms [38]. Prd1p and Pdr3p together confer resistance to several drugs through transcriptional activation of ABC transporter genes and members of the major facilitator super- family of drug efflux pumps, resulting in the expulsion of various structurally unrelated molecules. Pdr1p directly binds to xenobiotics to activate genes, and interacts physically and functionally with the Gal11p/MED15 subunit of the Mediator [39]. The Mediator co-activator interacts with RNA polymerase II [40], which is in agreement with our observation that transcription (mediated mainly by RNApolII) is one of the most significant biological processes the ACTMOD network is significantly enriched for (Figure 2E).

The Yap family belongs to the bZIP super-family of TFs and the majority of its members are conserved from yeast to human. One of the family members, Yap8 (Arr1), appears in Figure 3C and shows accelerated evolution to the average of the genome [41], which is consistent with the hypothesis that Yap8 is under positive selection to fulfill a wider genetic program required to deal with new environmental stimuli. Reb1 protein is an essential, auto-regulated DNA-binding protein that binds to many sites in the *S. cerevisiae* genome. Van Slyke *et al*. [42] showed that Reb1p is likely to play an important role in the complex regulation of *CLB2* product −a protein with a central distinct role among the cyclins− which will in turn influence the timing or progression of the cell cycle, or the budding process. Consequently, identification of other proteins that affect this promoter and their interactions will allow us to understand not only the regulatory circuitry of *CLB2*, but also the mechanisms by which cell cycle is regulated. Sko1 is also a basic leucine zipper (bZIP) TF of the ATF/CREB family. In a recent breakthrough on our understanding on cell cycle control, Niu *et al* [43] observed that over-expression of the TF Sko1 arrest cells at G1 phase by activating the pheromone response pathway, supporting the notion that many genes may gain function upon over-expression [43].

Surprisingly, the 7 highly interconnected TFs described above, regulate the expression of a gene with unknown function, *FMP43* (Figure 3A and C), whose expression level was very high under all conditions tested in this study and significantly differentially expressed by FA addition. The identification of FMP43 as one of the primary contributors of the environmental and internal sensing mechanisms to achieve network dynamics attracted our attention. Subsequently, we further explored how the phenotypic biomarkers and the network robustness are affected by redundancy and degeneracy of this protein and its human ortholog.

### Functional screening and post-translation predictions highlight the essential role of the FMP43 protein

Interestingly, the FMP43 protein belongs to a complex of 16 proteins (Figure 2E) with 10 of them being putative proteins with unknown function (Table S6). In addition, BRP44, a human brain protein, is the ortholog of FMP43 protein. We further investigated the role of FMP43 using a variety of bioinformatics prediction servers. The ProtFun2.2 server identified FMP43 as a structural protein −supporting our hypothesis for its putative role in the regulation of essential cellular responses to internal or external signaling− involved in the energy metabolism. FMP43 does not contain any transmembrane segments, it is a non-secretory protein, and despite its small size, it has 5 phosphorylation sites (Table S7). In the significantly differentially expressed genes of the ANOVA list, there were only 2 protein kinases, the Gut1 −a glycerol kinase− and the Sip2 kinases. Sip2 is one of the three β-subunits of the Snf1 complex and its loss (*sip2*Δ) results in accelerated aging phenotype [44]. If the Sip2 kinase is involved in the phosphorylation of FMP43, it is possible that the unique (among the Snf1 β-subunits) effect of Sip2 on aging could be reproduced through alterations in the expression level of *FMP43*.

We summarized the significant effects that the *FMP43* deletion had on yeast physiology in Table 2. The maximum specific growth rate was 0.34 h^−1^(corresponding to doubling time of ∼122 min) for the reference strain (CON) and 0.39 h^−1^ (corresponding to doubling time of ∼106 min) for the strain Δ*fmp43* (FMP), which corresponds to an approximately 15% growth rate increase. Similarly, a 13% increase in the specific growth rate caused by the deletion of *FMP43* was also observed when the medium was supplemented with FA. Specifically, the CON strain had maximum specific growth rate of 0.23 h^−1^ (corresponding to doubling time of ∼180 min), and the FMP strain had maximum specific growth rate of 0.26 h^−1^ (corresponding to doubling time of ∼160 min). This presumably indicates that the effect of FA on the FMP43 protein is exhibited as the result of a direct interaction rather than through a signaling cascade. Additionally, we found that the biomass yield of the FMP strain was increased by 72% compared with the biomass yield of the CON strain, which demonstrates that *FMP43* deletion triggered changes in the central carbon metabolism in favor of biomass production. This result correlated well with the observed increase in both the CO_2_ and O_2_ yields for the FMP strain (Table 2). However, when FA was added in the medium, the yields of the different metabolic products on glucose were not significantly altered (Table 2), possibly because the toxic effect of the high FA concentration (Table 1) partially counter-affected the phenotype of the *FMP43* mutant.

**Table 2:** Specific growth rates and production/consumption yields (C-mole/C-mole glucose) in the presence and absence of ferulic acid (FA) of the *S. cerevisiae* control strain (CON), the *S. cerevisiae* FMP43 deletion mutant (FMP) and the *S. cerevisiae* mutant with FMP43 protein being replaced by the human BRP44 orthologue (HUM).

Strains	(−) FA	μ_max_ (h^−1^)	biomass	O_2_	CO_2_	Ethanol
CON		0,34	0,107	0,081	0,115	0,442
FMP		0,39	0,184	0,093	0,142	0,454
HUM		0,34	0,144	0,077	0,108	0,437

	(+) FA					
CON		0,23	0,085	0,026	0,09	0,427
FMP		0,26	0,084	0,029	0,09	0,409
HUM		0,26	0,153	0,042	0,13	0,374

All values were calculated in batch culture (biological triplicates) during exponential growth phase on glucose, and identified by the linear relationship between the natural logarithm of biomass and the culture time.

In actively dividing cells, cell size reflects the balance between growth and division. Environmental or genetic perturbations, such as addition of a compound in the culture medium or gene deletions, could shift the balance in favor of growth. Such shift could be either due to higher growth rate of the cells or a delay in division, and it inevitably results in increase of the cell size [45], [46]. These principles apply to the present study, where a gene deletion resulted in higher growth rate (with or without FA in the culture medium), and thus higher cell size and biomass yield for the constructed mutant compared with the wild type. This interpretation also implies that cell cycle progression could be affected. A recent study showed that the ability of yeast cells to grow changes during the cell cycle. Specifically, during the normal cell cycle, cellular growth is slower at the passage through the G1/S-phase boundary, while the ability of cells to grow is higher in anaphase- and G1-arrested cells than in any other cell cycle stage [47]. If cell cycle progression in the FMP mutant is indeed altered, then we hypothesize that FMP43 protein is related to and somehow affects key regulatory mechanisms governing the cell cycle, as for instance the G1/S-phase control point. Additional experiments, to detect any cell cycle dissimilarity of the FMP strain compared to the CON strain, are needed. Flow cytometry experiments −using the propidium iodide to stain nuclear DNA or a more sensitive DNA stain− would be suitable to this direction.

The genome-wide transcription response of the CON and FMP strains, when both were grown in triplicates, resulted in 985 genes with differential expression (*p_adjusted_*<0.01). According to the GO term finder of the SGD database, the products of differentially expressed genes in the FMP relative to the CON strain showed functional enrichment for metabolic process (*p* = 2.45×10^−05^) and cellular component organization (*p* = 9.68×10^−05^). For the same gene list, GENECODIS tool has reported co-occurrence annotation enrichment for cellular respiration (BP) and mitochondrial ribosomes (CC) (*p* = 8.70×10^−06^).

The transcriptional changes were superimposed on the metabolic network to identify metabolic units that changed in response to the *FMP43* deletion. By using an algorithm that detects metabolic modules based on biologically significant changes in gene expression [33], the ‘reporter metabolites’, i.e. the metabolites around which significant coordinated gene expression occurs, were identified. The top reporter metabolites are presented in Table 3.

**Table 3:** Reporter metabolites around which most significant gene expression changes occurred in response to *FMP43* deletion in *S. cerevisiae*.

Metabolite	Number of neighbors	P-value
1,3-beta-D-Glucan	7	0,02
IMP	10	0,02
S-Adenosylmethioninamine	2	0,03
Mixt	2	0,03
L-Glutamate 5-semialdehyde	2	0,03
N-(L-Arginino)succinate	2	0,04
O-Phospho-4-hydroxy-L-threonine	2	0,04
3-Phosphoserine	2	0,04
Adenylylsulfate	3	0,04
Peptide	2	0,04
Orthophosphate	57	0,05
(R)-Pantoate	2	0,05
L-Citrulline	2	0,05
4-Phospho-L-aspartate	2	0,05
1-Phosphatidyl-1D-myo-inositol 3-phosphate	3	0,05

We evaluated the significance of these metabolites as putative biomarkers in human clinical studies by retrieving relevant information from the Human Metabolome Database (HMDB). HMDB (www.hmdb.ca) is a database containing detailed data on small molecule metabolites found in the human body. It was very interesting that abnormal concentration of 4 of the 13 top reporter metabolites in Table 3 have been associated with prostate cancer. By surveying the HMDB for metabolites that are associated with the human prostate cancer, 360 hits were found (data downloaded in August 2010). As the performed search returned hits for both key words used (‘prostate’ and ‘cancer’), we then filtered the list retrieved. During the filtering procedure, only hits that showed well-proven association to prostate cancer were kept. These are metabolites for which abnormal quantitative data under disease state exist. Hits that referred to either key words only, or positive hits restricted to specific groups of men (e.g. men exposed to N-N-dimethylformamide), or hits commonly used for prostate cancer treatment and prevention purposes, were eliminated.

The filtering procedure indicated 44 metabolites, whose abnormal concentration level is closely linked to the prostate cancer development and progression. On the next step, we investigated the overlap between this metabolite list and the whole set of metabolites reported in the REPORTER FEATURE software. The overlap group consisted of 9 metabolites. The significance of our finding, that 4 out of the top 13 identified reporter metabolites are associated with prostate cancer, was evaluated by implementing a hypergeometric distribution test, where the calculated *p*-value (1.33×10^−04^) confirmed the statistical significance of our finding.

### The human brain protein BRP44 restores the wild type phenotype of S. cerevisiae

To investigate the functionality of the human ortholog of the FMP43 protein in *S. cerevisiae*, we inserted the human gene in the genetic background of the yeast strain Δ*fmp43* (HUM). Surprisingly, the replacement of the FMP43 by the BRP44 protein reinstated the specific growth rate at the same value as the yeast control strain (CON) (Table 2). In addition to the specific growth rate, the expression of the BRP44 protein restored the majority of metabolite yields on glucose (O_2_, CO_2_ and ethanol) with the exception of biomass yield, which was significantly higher for the HUM strain compared with the yield of the CON strain (Table 2). However, when FA was added in the medium the growth characteristics of the HUM strain were different from those of the CON strain (Table 2), supporting our previous hypothesis on direct interaction between this small molecule and the target protein, in this case the BRP44 protein (or, as previously suggested, the FMP43 protein).

We verified this hypothesis by performing protein-ligand binding site predictions and molecular docking studies for the BRP44 protein and FA. Firstly, we performed homology modeling and structure validation of the BRP44 protein. A total of 62 PDB templates with different E-value cut offs for amino acid sequence similarity with BRP44 were obtained from PDB advanced search interface. These templates were shortlisted based on resolution, sequence similarity and secondary structure similarity (SOPMA), covering the maximum range of the BRP44 sequence. A total of three templates were shortlisted and their secondary structure alignments are shown in Table S9.

Three homology model structures for BRP44 were built using three different combinations of templates. The ProSA-derived *Z*-scores for BRP44 Model 1, BRP44 Model 2, and BRP44 Model 3 were −1.91, −1.91 and −0.24, respectively. Even though the *Z*-scores of BRP44 Model 1 and BRP44 Model 2 are similar, some distortions were observed in BRP44 Model 2 (not shown here). Ramachandran plot summaries of BRP44 Model 1 and its respective templates (obtained from Procheck program) are shown in Table S9. Based on the analysis of 118 structures at resolution of at least 2.0 Angstroms and with the R-factor being no greater than 20%, over 90% of a good quality model would be expected in the most favored regions (Procheck program). RMS deviations of BRP44 Model 1 and its respective templates are shown in Table S9. The overall statistics on structure quality as well as the overall model quality (*Z*-score obtained from ProSA-web) are provided in File S9.

**Figure 4:**
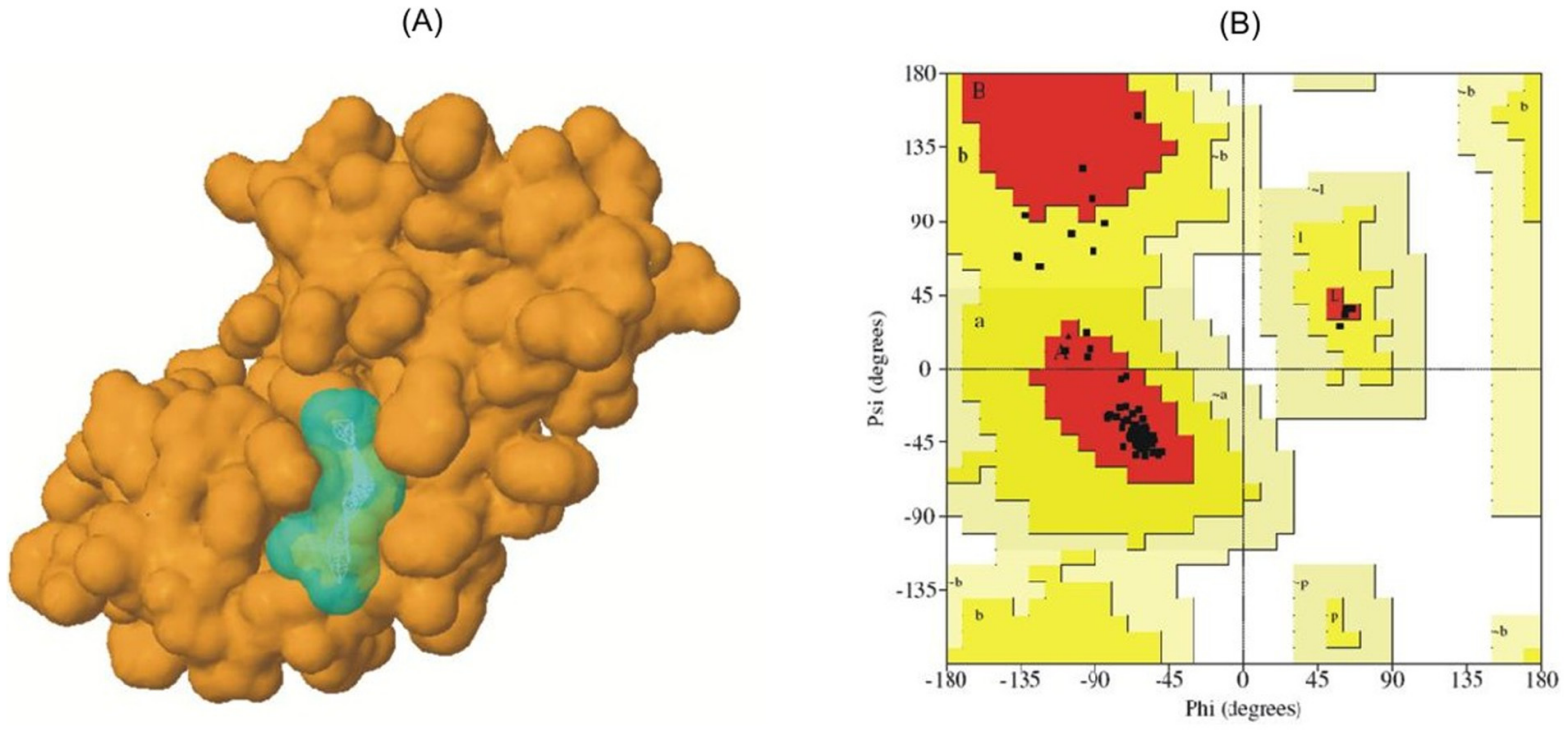
Homology modeling of the human brain protein BRP44 and ligand binding site prediction. (**A**) Molecular surface structure of BRP44 showing predicted binding site-1 in cyan. (**B**) Ramachandran plot of BRP44 structure obtained from Procheck program. None of the amino acid residues are in disallowed regions.

Two potential protein-ligand binding sites from a total of 10 predicted sites (using Q-SiteFinder) were selected for molecular docking studies. Predicted site-1 contained 13 amino acids surrounding the cavity and had a site volume of 201 Å^3^. Predicted site-2 contained 10 amino acids surrounding the cavity and had a site volume of 115 Å^3^. Molecular docking was performed against the BRP44 predicted binding site residues using FA as the ligand. Analysis of best ligand pose energies indicated that predicted site-1 had higher affinity for FA. The molecular surface structure of BRP44 with predicted binding site-1 is shown in Figure 4A along with the Ramachandran plot obtained from Procheck (Figure 4B).

Protein-ligand binding site prediction and docking studies were performed with AnFAEA (PDB Code: 1uwc). According to X-ray crystallography data from Katherine *et al*. [47], FA interacts with TYR80, LEU134, THR68 and SER133 in the binding site of feruloyl esterase (AnFAEA) from *Aspergillus niger*. LEU199 and ILE196 provide a hydrophobic environment in the binding pocket. The molecular docking study reveals that FA indeed has higher affinity for the binding site of BRP44 than the binding site of AnFAEA. The comparison of docking energies and the binding site residues are shown in File S9. According to X-ray crystallography data from Prates *et al*. [48], FA interacts with ASP980, TRP982, and ASN1023 in the binding site of feruloyl esterase module of xylanase 10B from *Clostridium thermocellum*. LEU958 provides a hydrophobic environment in the binding pocket.

Protein-ligand binding site prediction and docking studies were also performed with feruloyl esterase module of xylanase 10B (PDB Code: 1gkl). The molecular docking results when using 1 gkl and BRP44 reveal that in this case as well, FA has more affinity towards the binding site of BRP44. The comparison of docking energies and binding site residues are shown in Table S9.

The 3D structure of the FMP43 protein was determined and after applying the same computational approach as above, we observed that FA could bind in the two predicted sites of the FMP43 protein, while the structure of the FMP43 protein shows high similarity to 2BMX, a molecule of the *Mycobacterium tuberculosis* defense system that has a key role during oxidative stress (Table S9).

## Discussion

A plethora of computational approaches can be used to overcome the limitations of experimental techniques. Computational tools have become critical for the integration, representation and visualization of heterogeneous genomics, proteomics and biomedical data [49]. Experimental techniques, like yeast-two-hybrid, have enabled to pair-wisely screen protein-protein interactions [50]. Nevertheless, the study of protein complex data involving more than two partners is relatively restricted due to the limitations of the currently available high-throughput techniques. Computational approaches complement experimental methods for the detection of protein complexes using protein interaction data. The study of protein interaction networks is important not only from a theoretical point of view, but also has strong practical applications towards the development of new drugs, which could specifically interrupt or modulate protein interactions [51], [52].

Yeast has several features making it an ideal model to study, not only human disorders, but also the effect of nutraceuticals in the prevention or progress of a disease [53], [54]. Oxidative damage has long been considered as a primary threat for neurons, both in neurodegenerative disorders and aging. Free radicals, which among others are produced during normal metabolism, can trigger a series of events that disturb important aspects of the normal cellular function, including enzymatic activity, protein folding, transcription, ion channel activity, transporter function and other processes. Such damage may contribute to a broad range of diseases in the nervous system.

We presented a hypothesis-driven approach to elucidate the role of a small model antioxidant molecule and understand how the yeast cell tunes the flux of intermediates through metabolic routes and restructures the cellular transcriptome and proteome in the presence of such a compound. The phenome and metabolome data obtained from our well-controlled yeast cultivations clearly reflected the presence of an antioxidant compound −demonstrating once again that the systematic use of this simple eukaryotic organism can uncover important features of nutraceutical compounds.

By using this external stimulus and network biology tools, we identified a small, tightly connected sub-network reflecting the biological signature of the yeast cell during stress, and we identified the FMP43 protein −which has not been previously functionally characterized− as an important player in the network architecture. *In silico* analysis of *FMP43* transcriptional regulation and prediction of the post-translational modifications of the corresponding protein revealed a putative new cell cycle regulatory gene. This hypothesis was verified by the significant improvement of the specific growth rate of the yeast cell after deletion of *FMP43* and complements the recent finding about cell cycle delay phenotypes observed by over-expression of FMP31 [43], a similar protein with as well unknown function.

However, the linkage between antioxidant compounds and a growth-controlling gene (*FMP43*) needs further investigation. The ProtFun 2.2 server predicts FMP43 as a protein involved in oxidative energy metabolism, possibly due to its role in the metabolism of reactive oxygen species, which correlates well with the high metabolic necessity when time between cell division cycles is longer. This scenario could also explain the decrease in *FMP43* expression levels upon addition of the antioxidant molecule and the dual role of this protein in yeast.

Proteins can bind with many types of molecules using a wide variety of binding sites. For example, binding sites used by natural ligands or substrates, allosteric regulatory sites used by products or reversible/irreversible inhibitors, and ‘special’ binding sites at which an array of compounds −such as drugs and antioxidants− can bind [55]. Changes in the yeast phenotype stimulated by FA may be due to the disruption of an existing protein interaction, by changing the stability of the protein (or), by modulating the ability of the protein to interact with other molecules, (or) by initiating a series of signal transduction pathways upon binding to a particular protein. Following the complexity of protein-ligand interactions and fully characterizing by experimental means its effects on the protein-protein interactions is challenging.

To extend our findings to human cells and identify proteins that could serve as drug targets, we replaced the yeast FMP43 protein with its human ortholog BRP44 in the genetic background of the yeast strain Δ*fmp43*. The conservation of the two proteins was phenotypically evident, with BRP44 restoring the normal specific growth rate of the wild type, which was significantly increased by *FMP43* deletion. PPIs have been proven crucial for all biological processes. Hence, by performing PPI studies it is feasible to assign functions to uncharacterized proteins and understand the composition of protein complexes.

Taking into account the high potential of human PPIs for understanding disease mechanisms and signaling cascades, we investigated a representative part of the recently described human interactome [56]. We identified three interaction partners for the BRP44 protein, i.e. MAGED1, GABARAP and ACTC, all being disease-associated proteins according to the OMIM morbidmap (NCBI). Expression of members of several tumor-associated antigen families, as for instance of the MAGE family, is restricted to tumor cells and testes [57]. GABARAP is a GABA-A receptor-associated protein. Type-A receptors for the neurotransmitter GABA are ligand-gated chloride channels that mediate inhibitory neurotransmission. GABARAP expression has been detected in all tissues tested, namely heart, brain, placenta, lung, liver, skeletal muscle, kidney and pancreas, suggesting potential involvement of this protein in biological events other than interaction with GABA-A receptors [58]. Morgensen *et al.* [59] stated that *ACTC1* was the first sarcomeric gene described to cause two different cardiomyopathies when being mutated, and hypothesized that *ACTC1* mutations affecting sarcomere to the surrounding syncytium lead to dilated cardiomyopathy. In addition, the Human Protein Reference database indicates one more very interesting interaction of BRP44 with the ribosomal P1 protein. In the recent study of Martinez-Azorin *et al.* [60], the role of the ribosomal stalk P proteins modulating ribosomal activity was investigated in human cells using RNA interference. The loss of P1 protein produced a decrease in the growth rate of the cells −although the details of this association are not yet understood− which is in agreement with the growth effects observed in our study when BRP44, an interaction partner of P1 protein, was functionally expressed in yeast cells.

Molecular docking methods have been used for decades to determine the affinity of ligands/substrates towards the receptor/protein/enzyme, and thus have acquired a great importance in modern structure-based drug design [61]–[64]. A basic prerequisite for docking studies is the 3D structure of the proteins under study. Many proteins targeted for drug design do not have an experimentally determined structure, which makes the scope of docking studies limited. Homology modeling has been used to generate structural models of proteins, which can still be used as docking targets [65]–[67]. We predicted the 3D structure of the yeast FMP43 protein, which is transcriptionally regulated after FA addition. Homology modeling was also applied to predict the structure of the human BRP44 protein, which restored the normal phenotype in the FMP43 deletion yeast strain.

The second prerequisite for docking studies is to know the location of the ligand's binding site. The information related to binding sites can be obtained experimentally through co-crystallization of the protein-ligand complex. An alternative approach is to identify structural or sequence similarity with a known binding site, or use a computational tool to predict binding sites on the protein of interest [68]. The binding sites in homology models of the FMP43 and BRP44 proteins were computationally predicted, and further docking studies were performed using FA as the ligand. The results showed the affinity of FA towards both proteins, which is in line with our phenotypic experimental observations.

Comparing the protein-protein and protein-ligand interactions of simple cellular systems, for instance the yeast with the human system, promises much for the future −even though it limits the resolution of the results. The platform presented here strongly suggested anti-oxidant therapeutic targets −as demonstrated through identification and characterization of yeast and human orthrolog protein-protein interaction networks− but requires further *in vivo* validation. Similar to pharmacological therapeutic discovery, nutraceutical target identification and screening −while often limiting and resource-intensive− may be enhanced through the approach of yeast physiological and network biology analysis demonstrated here.

## Supporting Information

Table S1 Contains two Tables, in the first one there is a detailed description for the calculation of the Degree of Reduction (DOR) per C-mole of the metabolites identified during S. cerevisiae cultivations. The second Table contains the comparison of the intracellular and extracellular metabolite levels identified in S. cerevisiae cultivations in the presence and absence of ferulic acid (+FA and -FA) under aerobic (+O2) and anaerobic conditions (-O2). The values represent ratios while the last column indicates the DOR of each metabolite.
(0.16 MB PDF)

Table S2 Summary of the significant effects of FA on gene expression levels of S. cerevisiae. The list provided here specifies the FA-specific genes and contains genes with significant differential regulation when comparing the S. cerevisiae growth in the presence and absence of FA under aerobic and anaerobic conditions.
(0.12 MB PDF)

Table S3 Special protein-protein interaction considerations taken during the construction of the FA-specific primary network.
(0.05 MB PDF)

Table S4 Statistically significant protein complexes of the primary FA-specific network predicted by the MCODE clustering algorithm.
(0.18 MB PDF)

Table S5 Sub-cellular localization (Cerebral) and functional enrichment (BiNGO) analyses of the ACTMOD network.
(0.52 MB PDF)

Table S6 List of proteins participating in the FMP43 complex and their human orthology.
(0.07 MB PDF)

Table S7 Functional and structural characterization of the FMP43 protein using diverse bioinformatics tools and prediction servers (ProtFun, TMHMM, NetNES, SignalP, TargetP, NetPhosYeast, NetAcet and YinOYang servers).
(0.03 MB PDF)

Table S8 The gene deletion and over-expression strategies followed for the construction of the relevant yeast strains used in the present study, namely CEN.PK 113-5D/Î¿fmp43 and CEN.PK 113-5D/Î¿fmp43-pRS426-BRP44.
(0.61 MB PDF)

Table S9 Detailed file with the homology modelling, structure validation, protein-ligand binding site prediction and molecular docking studies of the BRP44 and FMP43 proteins.
(0.56 MB PDF)
