# Supplementary material for: Yeast Biological Networks Unfold the Interplay of Antioxidants, Genome and Phenotype, and Reveal a Novel Regulator of the Oxidative Stress Response
Source: PLoS One. 2010 Oct 25;5(10):e13606. doi: 10.1371/journal.pone.0013606 (PMC2963615; doi:10.1371/journal.pone.0013606)

Supplementary Material 1

Table 1: Calculation of the Degree Of Reduction (DOR) per C-mole of the metabolites identified during *S. cerevisiae* cultivations

Metabolite	MF	MW (g/mole)	MW (g/C-moles)	C	H	O	N	P	S	DOR (Redox Equivalents/C-mol)	SCALED DOR
NADP/NADPH	C21H30N7O17P3	745	372,50	2	1	17	7	3	0	2,00	0,32
alanine	C3H7NO2	89	29,67	3	7	2	1	0	0	5,67	0,89
glycine	C2H5NO2	75	37,50	2	5	2	1	0	0	5,50	0,87
2-amino butyric acid	C4H9NO2	103	34,33	3	9	2	1	0	0	6,33	1,00
2-isopropymalic acid	C7H12O5	176	25,14	7	12	5	0	0	0	5,00	0,79
valine	C5H11NO2	117	23,40	5	11	2	1	0	0	5,80	0,92
glyceric acid	C3H6O4	106	35,33	3	6	4	0	0	0	5,00	0,79
2-oxoglutaric acid	C5H6O5	146	29,20	5	6	5	0	0	0	4,20	0,66
leucine	C6H13NO2	131	21,83	6	13	2	1	0	0	5,83	0,92
isoleucine	C6H13NO2	131	21,83	6	13	2	1	0	0	5,83	0,92
3-oxoadipic acid	C6H8O5	160	26,67	6	8	5	0	0	0	4,50	0,71
2-oxoadipic acid	C6H8O5	160	26,67	6	8	5	0	0	0	4,50	0,71
phosphoenolpyruvic acid 2	C3H5O6P	167	55,67	3	5	6	0	1	0	3,67	0,58
4-amino-n-butyric acid (GABA)	C4H9NO2	103	25,75	4	9	2	1	0	0	5,75	0,91
proline	C5H9NO2	115	23,00	5	9	2	1	0	0	5,40	0,85
5-hydroxymethyl-2-furaldehyde 2	C6H6O3	126	21,00	6	6	3	0	0	0	4,83	0,76
Cis-Aconitic Acid	C6H6O6	174	29,00	6	6	6	0	0	0	3,83	0,61
threonine	C4H9NO3	119	29,75	4	9	3	1	0	0	5,25	0,83
serine	C3H7NO3	105	35,00	3	7	3	1	0	0	5,00	0,79
pyroglutamic acid	C5H7NO3	129	25,80	5	7	3	1	0	0	4,60	0,73
citric acid	C6H8O7	192	32,00	6	8	7	0	0	0	3,83	0,61
aspartic acid	C4H7NO4	133	33,25	4	7	4	1	0	0	4,25	0,67
asparagine	C4H8N2O3	132	33,00	4	8	0	2	0	0	5,75	0,91
myristic acid	C14H28O2	228	16,29	14	28	2	0	0	0	6,07	0,96
glutamic acid	C5H9NO4	147	29,40	5	9	4	1	0	0	4,60	0,73
N-acetyl-L-glutamate	C7H11NO5	189	27,00	7	11	5	1	0	0	4,43	0,70
D-2-amino adipic acid	C6H11NO4	161	26,83	6	11	4	1	0	0	4,83	0,76
phenylalanine	C9H11NO2	165	18,33	9	11	2	1	0	0	5,00	0,79
4-aminobenzoic acid	C7H7NO2	137	19,57	7	7	2	1	0	0	4,71	0,74
ornithine	C5H12N2O2	132	26,40	5	12	2	2	0	0	5,40	0,85
glutamine	C5H10N2O3	146	29,20	5	10	3	2	0	0	4,60	0,73
lysine	C6H14N2O2	146	24,33	6	14	2	2	0	0	5,50	0,87
histidine	C6H9N3O2	155	25,83	6	9	2	3	0	0	4,17	0,66
tyrosine	C9H11NO3	181	20,11	9	11	3	1	0	0	4,78	0,75
tryptophan	C11H12N2O2	204	18,55	11	12	2	2	0	0	4,64	0,73
Cystathionine	C7H14N2O4S	222	31,71	7	14	4	2	0	1	5,57	0,88
pyruvate	C3H4O3	88	29,33	3	4	3	0	0	0	5,00	0,79
malonic acid	C3H4O4	104	34,67	3	4	4	0	0	0	4,33	0,68
fumaric acid	C4H4O4	116	29,00	4	4	4	0	0	0	4,25	0,67
succinic acid	C4H6O4	118	29,50	4	6	4	0	0	0	4,75	0,75
lactic acid	C3H6O3	90	30,00	3	6	3	0	0	0	5,67	0,89
malic acid	C4H6O5	134	33,50	4	6	5	0	0	0	4,25	0,67
citraconate	C5H6O4	130	26,00	5	6	4	0	0	0	4,60	0,73
citramalate	C5H8O5	148	29,60	5	8	5	0	0	0	4,60	0,73
glutaric acid	C5H8O4	132	26,40	5	8	4	0	0	0	5,00	0,79
										**MEAN**	**0,77**

Table 2: Comparison of the intracellular and extracellular metabolite levels identified in *S. cerevisiae* cultivations in the presence (+FA) and absence (-FA) of ferulic acid under aerobic (+O_2) and anaerobic conditions (-O_2). The values represent ratios while the last column indicates the DOR of each metabolite

	INTRACELLULAR		EXTRACELLULAR		
**METABOLITES**	(+) O_2, (+) FA / (+) O_2, (-) FA	(-) O_2, (+) FA / (-) O_2, (-) FA	(+) O_2, (+) FA / (+) O_2, (-) FA	(-) O_2, (+) FA / (-) O_2, (-) FA	**DOR**
NADP/NADPH	0,412	0,641	5726	1,000	0,32
alanine	1,591	1,332	5,447	1,756	0,89
glycine	1,540	1,668	78519	1,983	0,87
2-amino butyric acid	1,550	1,000	113250	1,000	1,00
2-isopropymalic acid	0,665	1,295	3,305	32432	0,79
valine	3,206	1,641	11,049	1,278	0,92
glyceric acid	1,071	1,000	11919	1,000	0,79
2-oxoglutaric acid	1,150	0,000	2,815	1,744	0,66
leucine	1,736	1,566	8,844	1,000	0,92
isoleucine	2,300	0,824	23,371	1,778	0,92
3-oxoadipic acid	1,000	1,000	1184	1,000	0,71
2-oxoadipic acid	1,000	1,000	3496	1,000	0,71
phosphoenolpyruvic acid 2	1,016	1,000	6398	1,000	0,58
4-amino-n-butyric acid (GABA)	54308	1,000	54168	1,000	0,91
proline	1,024	0,451	5,027	1,218	0,85
5-hydroxymethyl-2-furaldehyde 2	1,000	1,000	1,553	1,000	0,76
Cis-Aconitic Acid	0,404	0,484	44815	17522	0,61
threonine	2,192	1,148	6,292	1,510	0,83
serine	0,641	0,902	7,128	1,000	0,79
pyroglutamic acid	0,845	1,000	3,825	1,000	0,73
citric acid	0,580	0,415	20,715	2,152	0,61
aspartic acid	1,473	1,030	5,000	1,860	0,67
asparagine	1,374	1,383	7,975	1,000	0,91
myristic acid	11265	0,000	0,000	2,220	0,96
glutamic acid	1,492	0,699	6,133	1,136	0,73
N-acetyl-L-glutamate	0,696	0,916	17174	1,000	0,70
D-2-amino adipic acid	0,449	1,244	44375	1,000	0,76
phenylalanine	0,600	0,568	10,466	2,296	0,79
4-aminobenzoic acid	0,000	1,000	0,000	1,000	0,74
ornithine	0,903	0,622	21,628	192505	0,85
glutamine	0,288	1,000	1,000	1,000	0,73
lysine	1,413	0,000	494706	1,000	0,87
histidine	0,958	0,305	27,480	1,000	0,66
tyrosine	1,124	0,329	35,681	59168	0,75
tryptophan	49994	1,000	49994	1,000	0,73
Cystathionine	1,520	0,835	23429	1,000	0,88
pyruvate	1,000	1,770	1,000	1,666	0,79
malonic acid	1,000	1,000	5,730	1,000	0,68
fumaric acid	0,923	0,000	26169	0,000	0,67
succinic acid	2,310	0,415	6,147	11,368	0,75
lactic acid	1,000	0,836	0,000	11,868	0,89
malic acid	1,050	1,000	26169	0,000	0,67
citraconate	4,546	17429	0,721	5,497	0,73
citramalate	3,317	4,075	1,721	3,425	0,73
glutaric acid	1,838	1,000	55896	1,000	0,79

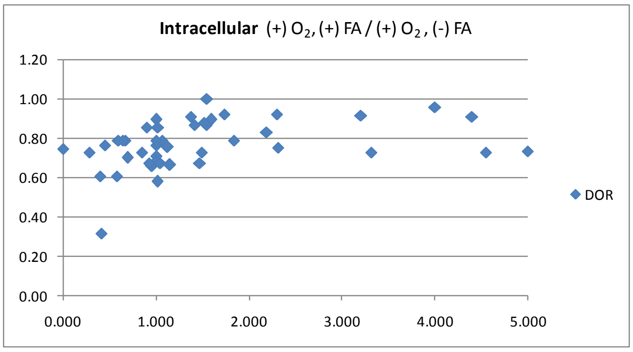

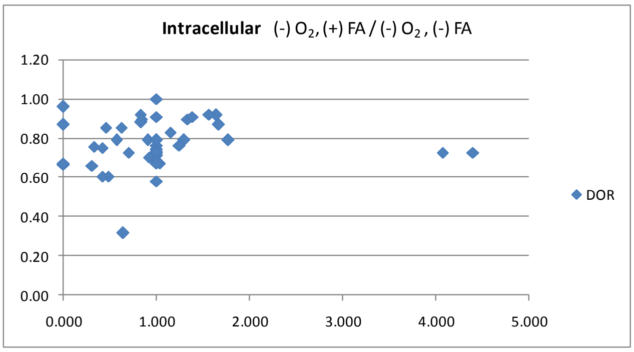

Supplement: Table S1 — Contains two Tables, in the first one there is a detailed description for the calculation of the Degree of Reduction (DOR) per C-mole of the metabolites identified during S. cerevisiae cultivations. The second Table contains the comparison of the intracellular and extracellular metabolite levels identified in S. cerevisiae cultivations in the presence and absence of ferulic acid (+FA and -FA) under aerobic (+O2) and anaerobic conditions (-O2). The values represent ratios while the last column indicates the DOR of each metabolite. (0.16 MB PDF) [file pone.0013606.s001.pdf]
