# Supplementary material for: Yeast Biological Networks Unfold the Interplay of Antioxidants, Genome and Phenotype, and Reveal a Novel Regulator of the Oxidative Stress Response
Source: PLoS One. 2010 Oct 25;5(10):e13606. doi: 10.1371/journal.pone.0013606 (PMC2963615; doi:10.1371/journal.pone.0013606)
Supplement: Table S2 — Summary of the significant effects of FA on gene expression levels of S. cerevisiae. The list provided here specifies the FA-specific genes and contains genes with significant differential regulation when comparing the S. cerevisiae growth in the presence and absence of FA under aerobic and anaerobic conditions. (0.12 MB PDF) [file pone.0013606.s002.pdf]

| | ID | LOGFC FAAERvsAER | LOGFC FAANAvsANA | F | P.Value | adj.P.Val | COMMON NAME | ALIAS | CHROMOSOME | DESCRIPTION |
|---|---|---|---|---|---|---|---|---|---|---|
| 2171 | YDR380W | 3,691699526 | 5,448953054 | 686,2142 | 2,41E-11 | 2,63388E-07 | ARO10 | | chromosome 4 | Phenylpyruvate decarboxylase, catalyzes decarboxylation of phenylpyruvate to phenylacetaldehyde, which is the first specific step in the Ehrlich pathway |
| 8081 | YLR155C | 0,237613669 | -3,062852394 | 518,025 | 9,58E-11 | 4,82E-07 | ASP3-1 | ASP3 | chromosome 12 | Cell-wall L-asparaginase II, involved in asparagine catabolism; expression is induced during nitrogen starvation; four copies of ASP3 are present in the genome reference strain S288C |
| 6040 | YHR137W | 1,969873805 | 4,535503522 | 275,9719 | 2,08E-09 | 4,54072E-06 | ARO9 | | chromosome 8 | Aromatic aminotransferase II, catalyzes the first step of tryptophan, phenylalanine, and tyrosine catabolism |
| 3176 | YAL062W | 1,982577323 | -2,021585801 | 200,1853 | 9,85E-09 | 9,78645E-06 | GDH3 | FUN51 | chromosome 1 | NADP(+)-dependent glutamate dehydrogenase, synthesizes glutamate from ammonia and alpha-ketoglutarate; rate of alpha-ketoglutarate utilization differs from Gdh1p; expression regulated by nitrogen and carbon sources |
| 4020 | YFR017C | 0,861060799 | -1,42998535 | 167,6529 | 2,32E-08 | 1,40595E-05 | | | chromosome 6 | Putative protein of unknown function; green fluorescent protein (GFP)-fusion protein localizes to the cytoplasm and is induced in response to the DNA-damaging agent MMS; YFR017C is not an essential gene |
| 4608 | YMR175W | 0,563315997 | -2,374722639 | 170,4855 | 2,14E-08 | 1,40595E-05 | SIP18 | | chromosome 13 | Protein of unknown function whose expression is induced by osmotic stress |
| 3020 | YIL057C | 0,434795359 | -4,208965106 | 153,6247 | 3,52E-08 | 1,49279E-05 | | | chromosome 9 | Putative protein of unknown function; expression induced under carbon limitation and repressed under high glucose |
| 3610 | YOL151W | 0,691289473 | 2,142860999 | 128,7509 | 8,2E-08 | 1,82848E-05 | GRE2 | | chromosome 15 | NADPH-dependent methylglyoxal reductase (D-lactaldehyde dehydrogenase); stress induced (osmotic, ionic, oxidative, heat shock and heavy metals); regulated by the HOG pathway |
| 5609 | YLR157W-C | 0,991408408 | -2,618706548 | 121,9559 | 1,06E-07 | 2,07609E-05 | | | | |
| 4936 | YOL052C-A | 0,258962408 | -1,387854354 | 102,1059 | 2,46E-07 | 3,32361E-05 | DDR2 | YOL053C-A\|DDRA2 | chromosome 15 | Multistress response protein, expression is activated by a variety of xenobiotic agents and environmental or physiological stresses |
| 644 | YDL085W | 0,742303863 | -1,531377468 | 101,023 | 2,59E-07 | 3,34708E-05 | NDE2 | NDH2 | chromosome 4 | Mitochondrial external NADH dehydrogenase, catalyzes the oxidation of cytosolic NADH; Nde1p and Nde2p are involved in providing the cytosolic NADH to the mitochondrial respiratory chain |
| 9295 | YFL051C | 0,334265652 | 1,202781879 | 95,62288 | 3,36E-07 | 3,66768E-05 | | | chromosome 6 | Putative protein of unknown function; YFL051C is not an essential gene |
| 9197 | YNR002C | 0,295128933 | -2,270554253 | 94,03457 | 3,63E-07 | 3,85281E-05 | ATO2 | FUN34 | chromosome 14 | Putative transmembrane protein involved in export of ammonia, a starvation signal that promotes cell death in aging colonies; phosphorylated in mitochondria; member of the TC 9.B.33 YaaH family; homolog of Ady2p and Y. lipolytica Gpr1p |
| 3313 | YJL163C | 0,472507699 | -1,1725994 | 90,41345 | 4,37E-07 | 4,1499E-05 | | | chromosome 10 | Putative protein of unknown function |
| 8873 | YLR136C | 0,792793982 | 1,802084962 | 88,78019 | 4,76E-07 | 4,22639E-05 | TIS11 | CTH2 | chromosome 12 | mRNA-binding protein expressed during iron starvation; binds to a sequence element in the 3'-untranslated regions of specific mRNAs to mediate their degradation; involved in iron homeostasis |
| 5835 | YPL230W | 0,51245363 | -1,371643745 | 88,27924 | 4,88E-07 | 4,27056E-05 | USV1 | | chromosome 16 | Putative transcription factor containing a C2H2 zinc finger; mutation affects transcriptional regulation of genes involved in protein folding, ATP binding, and cell wall biosynthesis |

| | | | | | | | | | | | |
|---|---|---|---|---|---|---|---|---|---|---|---|
| 5306 | YBR241C | 0,991470524 | -1,051856273 | 87,75309 | 5,02E-07 | 4,35026E-05 | | | | chromosome 2 | Putative transporter, member of the sugar porter family; green fluorescent protein (GFP)-fusion protein localizes to the vacuolar membrane; YBR241C is not an essential gene |
| 150 | YJL185C | 0,626878539 | -1,075800531 | 85,6314 | 5,63E-07 | 4,60943E-05 | | | | chromosome 10 | Putative protein of unknown function; mRNA is weakly cell cycle regulated, peaking in G2 phase; YJL185C is a non-essential gene |
| 7359 | YCL026C-B | -0,635951281 | 1,067686362 | 81,46436 | 7,11E-07 | 5,47388E-05 | | HBN1 | YCL027C-A | chromosome 3 | Putative protein of unknown function; similar to bacterial nitroreductases; green fluorescent protein (GFP)-fusion protein localizes to the cytoplasm and nucleus |
| 2546 | YNL160W | 0,441562251 | 1,345045298 | 76,04413 | 9,8E-07 | 6,45261E-05 | | YGP1 | | chromosome 14 | Cell wall-related secretory glycoprotein; induced by nutrient deprivation-associated growth arrest and upon entry into stationary phase; may be involved in adaptation prior to stationary phase entry; has similarity to Sps100p |
| 6704 | YMR206W | 1,19108045 | -2,476292294 | 74,87942 | 1,05E-06 | 6,63464E-05 | | | | chromosome 13 | Putative protein of unknown function; YMR206W is not an essential gene |
| 8375 | YCR046C | -0,127534031 | -1,001400408 | 73,75401 | 1,13E-06 | 6,89689E-05 | | IMG1 | | chromosome 3 | Mitochondrial ribosomal protein of the large subunit, required for respiration and for maintenance of the mitochondrial genome |
| 34 | YIL160C | 1,221672926 | -1,510417521 | 73,43749 | 1,15E-06 | 6,95811E-05 | | POT1 | POX3\|FOX3 | chromosome 9 | 3-ketoacyl-CoA thiolase with broad chain length specificity, cleaves 3-ketoacyl-CoA into acyl-CoA and acetyl-CoA during beta-oxidation of fatty acids |
| 3938 | YBR037C | -0,675432791 | -1,204215042 | 72,63683 | 1,21E-06 | 7,16258E-05 | | SCO1 | PET161 | chromosome 2 | Copper-binding protein of the mitochondrial inner membrane, required for cytochrome c oxidase activity and respiration; may function to deliver copper to cytochrome c oxidase; has similarity to thioredoxins |
| 2996 | YLR108C | -0,256710445 | 1,365846585 | 70,54477 | 1,39E-06 | 7,6164E-05 | | | | chromosome 12 | Protein of unknown function; green fluorescent protein (GFP)-fusion protein localizes to the nucleus; YLR108C is not an esssential gene |
| 7959 | YMR174C | 0,578824956 | -1,500515542 | 70,48739 | 1,39E-06 | 7,6164E-05 | | PAI3 | | chromosome 13 | Cytoplasmic proteinase A (Pep4p) inhibitor, dependent on Pbs2p and Hog1p protein kinases for osmotic induction; intrinsically unstructured, N-terminal half becomes ordered in the active site of proteinase A upon contact |
| 6556 | YLR156W | 0,424516657 | -3,569347115 | 68,72508 | 1,57E-06 | 8,11099E-05 | | | | chromosome 12 | Putative protein of unknown function; exhibits a two-hybrid interaction with Jsn1p in a large-scale analysis |
| 4822 | YMR006C | -0,543453968 | -1,754282398 | 68,02251 | 1,64E-06 | 8,23548E-05 | | PLB2 | | chromosome 13 | Phospholipase B (lysophospholipase) involved in phospholipid metabolism; displays transacylase activity in vitro; overproduction confers resistance to lysophosphatidylcholine |
| 5191 | YNL200C | 0,74675745 | -1,071309276 | 66,54574 | 1,82E-06 | 8,67312E-05 | | | | chromosome 14 | Putative protein of unknown function; the authentic, non-tagged protein is detected in highly purified mitochondria in high-throughput studies |
| 8358 | YGL248W | 1,048186996 | 1,03560511 | 65,02978 | 2,02E-06 | 9,0964E-05 | | PDE1 | | chromosome 7 | Low-affinity cyclic AMP phosphodiesterase, controls glucose and intracellular acidification-induced cAMP signaling, target of the cAMP-protein kinase A (PKA) pathway; glucose induces transcription and inhibits translation |
| 5388 | YHR091C | 0,043513693 | -1,222795653 | 63,44653 | 2,26E-06 | 9,66138E-05 | | MSR1 | | chromosome 8 | Mitochondrial arginyl-tRNA synthetase |
| 1450 | YLR237W | -0,952169169 | -1,062528234 | 62,88637 | 2,36E-06 | 9,68457E-05 | | THI7 | THI10 | chromosome 12 | Plasma membrane transporter responsible for the uptake of thiamine, member of the major facilitator superfamily of transporters; mutation of human ortholog causes thiamine-responsive megaloblastic anemia |

| | | | | | | | | | | |
|---|---|---|---|---|---|---|---|---|---|---|
| 10131 | YBR056W-A | 1,977021072 | 1,238193457 | 62,46365 | 2,43E-06 | 9,80463E-05 | | | chromosome 2 | Dubious open reading frame unlikely to encode a protein, based on available experimental and comparative sequence data; partially overlaps the dubious ORF YBR056C-B |
| 8076 | YGR243W | 0,66343632 | -1,420382773 | 61,74148 | 2,56E-06 | 0,000101175 | | | chromosome 7 | Putative protein of unknown function; the authentic, non-tagged protein is detected in highly purified mitochondria in high-throughput studies |
| 9312 | YIL072W | -0,962688696 | 1,10603186 | 61,77215 | 2,56E-06 | 0,000101175 | HOP1 | | chromosome 9 | Meiosis-specific DNA binding protein that displays Red1p dependent localization to the unsynapsed axial-lateral elements of the synaptonemal complex; required for homologous chromosome synapsis and chiasma formation |
| 5124 | YIL113W | -0,388606869 | -1,797618667 | 60,58305 | 2,8E-06 | 0,000104758 | SDP1 | | chromosome 9 | Stress-inducible dual-specificity MAP kinase phosphatase, negatively regulates Slt2p MAP kinase by direct dephosphorylation, diffuse localization under normal conditions shifts to punctate localization after heat shock |
| 309 | YJL047C-A | -0,480398434 | -1,052971589 | 58,16713 | 3,37E-06 | 0,000117946 | | | chromosome 10 | Putative protein of unknown function |
| 7259 | YOR226C | -0,256224656 | 1,294505653 | 56,31139 | 3,9E-06 | 0,0001297 | ISU2 | NUA2 | chromosome 15 | Conserved protein of the mitochondrial matrix, required for synthesis of mitochondrial and cytosolic iron-sulfur proteins, performs a scaffolding function in mitochondria during Fe/S cluster assembly; isu1 isu2 double mutant is inviable |
| 10540 | YGR260W | 0,144332067 | -1,016908731 | 55,97259 | 4,01E-06 | 0,000131462 | TNA1 | | chromosome 7 | High affinity nicotinic acid plasma membrane permease, responsible for uptake of low levels of nicotinic acid; expression of the gene increases in the absence of extracellular nicotinic acid or para-aminobenzoate (PABA) |
| 10334 | YLR121C | 1,700836194 | 1,287091851 | 55,37763 | 4,21E-06 | 0,000136232 | YPS3 | YPS4 | chromosome 12 | Aspartic protease, attached to the plasma membrane via a glycosylphosphatidylinositol (GPI) anchor |
| 2568 | YLR204W | -0,619522369 | -1,142267928 | 55,37648 | 4,21E-06 | 0,000136232 | QRI5 | COX24 | chromosome 12 | Mitochondrial inner membrane protein, required for accumulation of spliced COX1 mRNA; may have an additional role in translation of COX1 mRNA |
| 800 | YOR178C | 1,299123279 | -1,397295311 | 54,9755 | 4,36E-06 | 0,000138349 | GAC1 | | chromosome 15 | Regulatory subunit for Glc7p type-1 protein phosphatase (PP1), tethers Glc7p to Gsy2p glycogen synthase, binds Hsf1p heat shock transcription factor, required for induction of some HSF-regulated genes under heat shock |
| 1673 | YJL063C | -0,618428633 | -1,275850457 | 53,2597 | 5,03E-06 | 0,000150351 | MRPL8 | HRD238 | chromosome 10 | Mitochondrial ribosomal protein of the large subunit |
| 1290 | YPL134C | -0,894879907 | -1,442333289 | 52,61851 | 5,31E-06 | 0,000155106 | ODC1 | | chromosome 16 | Mitochondrial inner membrane transporter, exports 2-oxoadipate and 2-oxoglutarate from the mitochondrial matrix to the cytosol for lysine and glutamate biosynthesis and lysine catabolism; suppresses, in multicopy, an fmc1 null mutation |
| 1704 | YDL222C | 0,999977536 | -1,157806175 | 51,65126 | 5,78E-06 | 0,00016513 | FMP45 | | chromosome 4 | Integral membrane protein localized to mitochondria (untagged protein) and eisosomes, immobile patches at the cortex associated with endocytosis; sporulation and sphingolipid content are altered in mutants; has homologs SUR7 and YNL194C |
| 10553 | YLR162W-A | -0,684589265 | -2,524525327 | 51,40162 | 5,9E-06 | 0,000166732 | | | chromosome 12 | Putative protein of unknown function identified by fungal homology comparisons and RT-PCR |
| 8198 | YIL101C | 0,266147181 | -1,291140017 | 48,91882 | 7,38E-06 | 0,000192585 | XBP1 | | chromosome 9 | Transcriptional repressor that binds to promoter sequences of the cyclin genes, CYS3, and SMF2; expression is induced by stress or starvation during mitosis, and late in meiosis; member of the Swi4p/Mbp1p family; potential Cdc28p substrate |

| | | | | | | | | | | |
|---|---|---|---|---|---|---|---|---|---|---|
| 10101 | YDR033W | 0,857403403 | 1,994951887 | 47,87161 | 8,13E-06 | 0,00019577 | MRH1 | | chromosome 4 | Protein that localizes primarily to the plasma membrane, also found at the nuclear envelope; the authentic, non-tagged protein is detected in mitochondria in a phosphorylated state; has similarity to Hsp30p and Yro2p |
| 10140 | YDR115W | -0,948040079 | -1,155569779 | 48,11422 | 7,95E-06 | 0,00019577 | | | chromosome 4 | Putative mitochondrial ribosomal protein of the large subunit, has similarity to E. coli L34 ribosomal protein; required for respiratory growth, as are most mitochondrial ribosomal proteins |
| 7311 | YMR081C | 0,148726091 | -1,779377717 | 47,72831 | 8,24E-06 | 0,00019672 | ISF1 | MBR3 | chromosome 13 | Serine-rich, hydrophilic protein with similarity to Mbr1p; overexpression suppresses growth defects of hap2, hap3, and hap4 mutants; expression is under glucose control; cotranscribed with NAM7 in a cyp1 mutant |
| 9830 | YER018C | -0,176785218 | -1,002376908 | 47,62762 | 8,32E-06 | 0,000198127 | SPC25 | | chromosome 5 | Component of the evolutionarily conserved kinetochore-associated Ndc80 complex (Ndc80p-Nuf2p-Spc24p-Spc25p); involved in chromosome segregation, spindle checkpoint activity and kinetochore clustering |
| 494 | YOL071W | -0,123434201 | -1,069197648 | 47,52634 | 8,4E-06 | 0,000199592 | EMI5 | | chromosome 15 | Non-essential protein of unknown function required for transcriptional induction of the early meiotic-specific transcription factor IME1, also required for sporulation |
| 5105 | YNR019W | 0,000797378 | 1,436377265 | 47,46635 | 8,45E-06 | 0,000200194 | ARE2 | SAT1 | chromosome 14 | Acyl-CoA:sterol acyltransferase, isozyme of Are1p; endoplasmic reticulum enzyme that contributes the major sterol esterification activity in the presence of oxygen |
| 7469 | YGR289C | 0,545899246 | -1,754695349 | 46,97888 | 8,85E-06 | 0,000206193 | MAL11 | MALT\|AGT1 | chromosome 7 | Maltose permease, inducible high-affinity maltose transporter (alpha-glucoside transporter); encoded in the MAL1 complex locus; member of the 12 transmembrane domain superfamily of sugar transporters |
| 2411 | YNR037C | -0,191322446 | -1,024889598 | 46,71883 | 9,07E-06 | 0,000208273 | RSM19 | | chromosome 14 | Mitochondrial ribosomal protein of the small subunit, has similarity to E. coli S19 ribosomal protein |
| 6110 | YKL137W | -0,772481827 | -1,115141093 | 46,1889 | 9,55E-06 | 0,000214227 | | | chromosome 11 | Putative protein of unknown function; mutation results in growth defect on a non-fermentable (respiratory) carbon source |
| 6687 | YGL208W | -0,030809618 | -1,228156281 | 44,82489 | 1,09E-05 | 0,000232491 | SIP2 | SPM2 | chromosome 7 | One of three beta subunits of the Snf1 serine/threonine protein kinase complex involved in the response to glucose starvation; null mutants exhibit accelerated aging; N-myristoylprotein localized to the cytoplasm and the plasma membrane |
| 683 | YLR346C | 2,563230679 | -1,611802633 | 44,74877 | 1,1E-05 | 0,000233349 | | | chromosome 12 | Putative protein of unknown function found in mitochondria; expression is regulated by transcription factors involved in pleiotropic drug resistance, Pdr1p and Yrr1p; YLR346C is not an essential gene |
| 1541 | YKL187C | 0,284030256 | -1,102935029 | 43,43426 | 1,26E-05 | 0,000253244 | | | chromosome 11 | Putative protein of unknown function; the authentic, non-tagged protein is detected in a phosphorylated state in highly purified mitochondria in high-throughput studies |
| 6824 | YJL077W-B | 0,234769188 | -1,247060342 | 43,11579 | 1,3E-05 | 0,000258697 | | | chromosome 10 | Putative protein of unknown function; identified by gene-trapping, microarray-based expression analysis, and genome-wide homology searching |
| 5809 | YOR150W | -0,668736173 | -1,023951945 | 41,84954 | 1,48E-05 | 0,000282184 | MRPL23 | | chromosome 15 | Mitochondrial ribosomal protein of the large subunit |
| 9158 | YHR096C | 0,668319063 | -1,446647844 | 40,93909 | 1,63E-05 | 0,000298239 | HXT5 | | chromosome 8 | Hexose transporter with moderate affinity for glucose, induced in the presence of non-fermentable carbon sources, induced by a decrease in growth rate, contains an extended N-terminal domain relative to other HXTs |
| 5832 | YDR462W | -0,462764686 | -1,06236729 | 40,56862 | 1,7E-05 | 0,000302591 | MRPL28 | | chromosome 4 | Mitochondrial ribosomal protein of the large subunit |
| 5064 | YJL066C | 0,425787866 | -1,108353493 | 40,30457 | 1,75E-05 | 0,000308184 | MPM1 | | chromosome 10 | Mitochondrial membrane protein of unknown function, contains no hydrophobic stretches |
| 5880 | YDL045W-A | -0,902648753 | -1,025077847 | 39,74283 | 1,86E-05 | 0,000319593 | MRP10 | | chromosome 4 | Mitochondrial ribosomal protein of the small subunit |

| | | | | | | | | | | | |
|---|---|---|---|---|---|---|---|---|---|---|---|
| 4036 | YML129C | -0,401022462 | -1,201818838 | 39,41606 | 1,93E-05 | 0,000328026 | | COX14 | | chromosome 13 | Mitochondrial membrane protein, involved in translational regulation of Cox1p and assembly of cytochrome c oxidase (complex IV); associates with complex IV assembly intermediates and complex III/complex IV supercomplexes |
| 6717 | YKL218C | -0,57220883 | 1,684099916 | 39,32853 | 1,95E-05 | 0,000329955 | | SRY1 | | chromosome 11 | 3-hydroxyaspartate dehydratase, deaminates L-threo-3-hydroxyaspartate to form oxaloacetate and ammonia; required for survival in the presence of hydroxyaspartate |
| 4149 | YMR107W | 0,503919356 | -1,33212074 | 39,20789 | 1,97E-05 | 0,000331916 | | SPG4 | | chromosome 13 | Protein required for survival at high temperature during stationary phase; not required for growth on nonfermentable carbon sources |
| 41 | YOR072W-B | -0,975766744 | -2,046968283 | 38,76725 | 2,07E-05 | 0,000342487 | | | | chromosome 15 | Putative protein of unknown function; identified by expression profiling and mass spectrometry |
| 10037 | YDR070C | 0,424031438 | -1,049324131 | 38,62257 | 2,11E-05 | 0,000343369 | | | | chromosome 4 | Putative protein of unknown function; the authentic, non-tagged protein is detected in highly purified mitochondria in high-throughput studies |
| 7964 | YIR014W | -0,836759216 | -1,354319038 | 38,6124 | 2,11E-05 | 0,000343369 | | | | chromosome 9 | Putative protein of unknown function; green fluorescent protein (GFP)-fusion protein localizes to the vacuole; expression directly regulated by the metabolic and meiotic transcriptional regulator Ume6p; YIR014W is a non-essential gene |
| 3403 | YBR262C | -0,662724807 | -1,283220658 | 38,06574 | 2,25E-05 | 0,000358059 | | | FMP51 | chromosome 2 | Protein of unknown function; non-tagged protein is detected in purified mitochondria in high-throughput studies; null mutant displays decreased frequency of mitochondrial genome loss and reduced growth rate in minimal glycerol media |
| 3292 | YPR166C | -0,971570973 | -1,121675871 | 37,80299 | 2,32E-05 | 0,000365537 | | MRP2 | | chromosome 16 | Mitochondrial ribosomal protein of the small subunit |
| 8448 | YGR138C | 1,492150583 | 2,511727451 | 37,14198 | 2,5E-05 | 0,000384662 | | TPO2 | | chromosome 7 | Polyamine transport protein specific for spermine; localizes to the plasma membrane; transcription of TPO2 is regulated by Haa1p; member of the major facilitator superfamily |
| 7292 | YKL107W | 1,336720098 | -1,044993617 | 37,04461 | 2,53E-05 | 0,000387463 | | | | chromosome 11 | Putative protein of unknown function |
| 8222 | YPL265W | 3,156170538 | -1,10029184 | 36,64164 | 2,66E-05 | 0,000400239 | | DIP5 | | chromosome 16 | Dicarboxylic amino acid permease, mediates high-affinity and high-capacity transport of L-glutamate and L-aspartate; also a transporter for Gln, Asn, Ser, Ala, and Gly |
| 9204 | YDR041W | -0,844564884 | -1,242928772 | 36,21302 | 2,8E-05 | 0,000413876 | | RSM10 | | chromosome 4 | Mitochondrial ribosomal protein of the small subunit, has similarity to E. coli S10 ribosomal protein; essential for viability, unlike most other mitoribosomal proteins |
| 10275 | YOR255W | 0,301967639 | -1,239936626 | 35,97024 | 2,88E-05 | 0,000422171 | | OSW1 | | chromosome 15 | Protein involved in sporulation; required for the construction of the outer spore wall layers; required for proper localization of Spo14p |
| 2852 | YDR178W | 0,022569365 | -1,343412676 | 35,75763 | 2,95E-05 | 0,000428028 | | SDH4 | ACN18 | chromosome 4 | Membrane anchor subunit of succinate dehydrogenase (Sdh1p, Sdh2p, Sdh3p, Sdh4p), which couples the oxidation of succinate to the transfer of electrons to ubiquinone |
| 1053 | YBR108W | 0,948149872 | 1,138028966 | 35,72593 | 2,96E-05 | 0,000428545 | | | | chromosome 2 | Protein interacting with Rsv167p; null mutant displays decreased frequency of mitochondrial genome loss (petite formation) and severe growth defect in minimal glycerol media |
| 3747 | YPL054W | 0,295411155 | -1,247986538 | 35,37188 | 3,1E-05 | 0,00044108 | | LEE1 | | chromosome 16 | Zinc-finger protein of unknown function |
| 1717 | YER093C-A | -0,78844114 | -1,075163328 | 35,31485 | 3,12E-05 | 0,000443026 | | | | chromosome 5 | Putative protein of unknown function; YER093C-A contains an intron; null mutant displays increased frequency of mitochondrial genome loss (petite formation) |

| | | | | | | | | | | |
|---|---|---|---|---|---|---|---|---|---|---|
| 10052 | YDR059C | 0,383975937 | -1,046798716 | 35,18918 | 3,17E-05 | 0,000446451 | UBC5 | | chromosome 4 | Ubiquitin-conjugating enzyme that mediates selective degradation of short-lived and abnormal proteins, central component of the cellular stress response; expression is heat inducible |
| 5282 | YKR080W | -0,755675452 | 1,054934058 | 34,61646 | 3,4E-05 | 0,000470862 | MTD1 | | chromosome 11 | NAD-dependent 5,10-methylenetetrahydrafolate dehydrogenase, plays a catalytic role in oxidation of cytoplasmic one-carbon units; expression is regulated by Bas1p and Bas2p, repressed by adenine, and may be induced by inositol and choline |
| 4029 | YER182W | -0,642826982 | -1,499878965 | 34,57353 | 3,42E-05 | 0,0004728 | | | chromosome 5 | Putative protein of unknown function; the authentic, non-tagged protein is detected in highly purified mitochondria in high-throughput studies |
| 8632 | YMR157C | -0,684940401 | -1,052915216 | 33,80707 | 3,77E-05 | 0,000503713 | | FMP39 | chromosome 13 | Protein of unknown function; non-tagged protein is detected in purified mitochondria in high-throughput studies; null mutant displays increased frequency of mitochondrial genome loss and reduced growth rate in minimal glycerol media |
| 4589 | YDL245C | -0,129733279 | -2,605647179 | 33,38695 | 3,97E-05 | 0,000521424 | HXT15 | | chromosome 4 | Protein of unknown function with similarity to hexose transporter family members, expression is induced by low levels of glucose and repressed by high levels of glucose |
| 9986 | YMR316W | 2,234465229 | 1,418335416 | 33,1878 | 4,08E-05 | 0,000529323 | DIA1 | | chromosome 13 | Protein of unknown function, involved in invasive and pseudohyphal growth; green fluorescent protein (GFP)-fusion protein localizes to the cytoplasm in a punctate pattern |
| 531 | YHL032C | 0,582879803 | -1,063563918 | 32,43339 | 4,5E-05 | 0,000564246 | GUT1 | | chromosome 8 | Glycerol kinase, converts glycerol to glycerol-3-phosphate; glucose repression of expression is mediated by Adr1p and Ino2p-Ino4p; derepression of expression on non-fermentable carbon sources is mediated by Opi1p and Rsf1p |
| 10391 | YER053C-A | 0,767829679 | 1,756784271 | 32,15568 | 4,67E-05 | 0,000580139 | | | chromosome 5 | Putative protein of unknown function; green fluorescent protein (GFP)-fusion protein localizes to the endoplasmic reticulum |
| 1742 | YJR150C | 0,044285157 | -5,301331726 | 31,51821 | 5,09E-05 | 0,000613962 | DAN1 | CCW13 | chromosome 10 | Cell wall mannoprotein with similarity to Tir1p, Tir2p, Tir3p, and Tir4p; expressed under anaerobic conditions, completely repressed during aerobic growth |
| 1205 | YJL100W | -0,171686643 | 1,334670757 | 31,02679 | 5,44E-05 | 0,000640354 | LSB6 | | chromosome 10 | Phosphatidylinositol 4-kinase that binds Las17p, which is a homolog of human Wiskott-Aldrich Syndrome protein involved in actin patch assembly and actin polymerization |
| 2825 | YKR071C | 0,655252545 | 1,174317462 | 30,57973 | 5,79E-05 | 0,000669002 | DRE2 | | chromosome 11 | Protein of unknown function; mutation displays synthetic lethal interaction with the pol3-13 allele of CDC2 |
| 1155 | YMR280C | 0,71852095 | -1,604813072 | 30,5399 | 5,82E-05 | 0,000672016 | CAT8 | MSP8\|DIL1 | chromosome 13 | Zinc cluster transcriptional activator necessary for derepression of a variety of genes under non-fermentative growth conditions, active after diauxic shift, binds carbon source responsive elements |
| 4358 | YDL194W | 0,377199463 | -1,016693127 | 30,10173 | 6,19E-05 | 0,000702037 | SNF3 | | chromosome 4 | Plasma membrane glucose sensor that regulates glucose transport; has 12 predicted transmembrane segments; long cytoplasmic C-terminal tail is required for low glucose induction of hexose transporter genes HXT2 and HXT4 |
| 7450 | YBL059C-A | -0,697829553 | -1,241449302 | 29,89443 | 6,38E-05 | 0,000711177 | | | chromosome 2 | Putative protein of unknown function; contains twin Cx9C motifs that can form coiled coil-helix-coiled-coil helix fold; green fluorescent protein (GFP)-fusion protein localizes to the cytoplasm and nucleus |
| 27 | YKL138C | -0,54709679 | -1,273527803 | 29,88701 | 6,38E-05 | 0,000711177 | MRPL31 | | chromosome 11 | Mitochondrial ribosomal protein of the large subunit |

| | | | | | | | | | | | |
|---|---|---|---|---|---|---|---|---|---|---|---|
| 2378 | YNL270C | 0,705802905 | -1,870004397 | 29,60351 | 6,65E-05 | 0,000729163 | | ALP1 | APL1 | chromosome 14 | Arginine transporter; expression is normally very low and it is unclear what conditions would induce significant expression |
| 6589 | YMR070W | -0,353791933 | 1,154311571 | 29,49447 | 6,75E-05 | 0,000734978 | | MOT3 | ROX7 | chromosome 13 | Nuclear transcription factor with two Cys2-His2 zinc fingers; involved in repression of a subset of hypoxic genes by Rox1p, repression of several DAN/TIR genes during aerobic growth, and repression of ergosterol biosynthetic genes |
| 10382 | YHL044W | -0,969358782 | -1,363235747 | 29,34504 | 6,9E-05 | 0,000745032 | | | | chromosome 8 | Putative integral membrane protein, member of DUP240 gene family; green fluorescent protein (GFP)-fusion protein localizes to the plasma membrane in a punctate pattern |
| 7299 | YDL039C | 2,140691692 | 2,096266523 | 28,51534 | 7,79E-05 | 0,000816842 | | PRM7 | | chromosome 4 | Pheromone-regulated protein, predicted to have one transmembrane segment; promoter contains Gcn4p binding elements |
| 5473 | YCR071C | -0,533342908 | -1,000733464 | 28,09063 | 8,3E-05 | 0,000856982 | | IMG2 | | chromosome 3 | Mitochondrial ribosomal protein of the small subunit |
| 3774 | YJL079C | -0,20902113 | -1,061304492 | 27,99771 | 8,41E-05 | 0,00086049 | | PRY1 | | chromosome 10 | Protein of unknown function, has similarity to Pry2p and Pry3p and to the plant PR-1 class of pathogen related proteins |
| 1533 | YDR381C-A | -0,955418163 | -1,337873633 | 27,87519 | 8,57E-05 | 0,000872826 | | | | chromosome 4 | Protein of unknown function, localized to the mitochondrial outer membrane |
| 4038 | YPL183W-A | -0,653386727 | -1,01368538 | 27,09863 | 9,65E-05 | 0,000951981 | | | GON5 | chromosome 16 | Homolog of the prokaryotic ribosomal protein L36, likely to be a mitochondrial ribosomal protein coded in the nuclear genome |
| 5640 | YPL201C | 0,735767866 | -1,927819251 | 27,03631 | 9,74E-05 | 0,000959116 | | YIG1 | | chromosome 16 | Protein that interacts with glycerol 3-phosphatase and plays a role in anaerobic glycerol production; localizes to the nucleus and cytosol |
