# Supplementary material for: Yeast Biological Networks Unfold the Interplay of Antioxidants, Genome and Phenotype, and Reveal a Novel Regulator of the Oxidative Stress Response
Source: PLoS One. 2010 Oct 25;5(10):e13606. doi: 10.1371/journal.pone.0013606 (PMC2963615; doi:10.1371/journal.pone.0013606)
Supplement: Table S3 — Special protein-protein interaction considerations taken during the construction of the FA-specific primary network. (0.05 MB PDF) [file pone.0013606.s003.pdf]

FA-specific primary network construction - Violation of the protein-protein interaction (PPI) confidence threshold

During the construction of the FA-specific primary network, a strict threshold on the confidence of PPIs to be included in subsequent steps of network analysis has been chosen; only PPIs with confidence equal to or higher than 0.6 have been considered. However, for few cases, exceptions have been made and the threshold mentioned above was violated if the excepted proteins show particularly interesting interactions (e.g., interaction with proteins involved in stress responses), or interact with a protein encoded by a significantly differentially expressed gene present in the ANOVA-derived gene list (106 genes), or their reported interaction scores are slightly lower than 0.6.

For instance, YLR346C had only one interaction satisfying the defined threshold. However, all the rest interactions were kept due to their high interest (e.g., involvement in multidrug resistance, participation of the human homologue protein in bile transport processes etc). Also, these interactions have a score close to the defined threshold (approximately 0.55). Moreover, five genes encoding for members of the UPF0041 protein family (FMP51, FMP10, FMP39, FMP41 and FMP43) appeared in the initial list of 106 significantly differentially expressed genes. This is an interesting group of proteins, as their function is still unknown and thus worth of investigating further, while *FMP43* gene has a human orthologue encoding for the brain protein 44 (BRP44). The corresponding interaction scores for this group of proteins, except one (0.655), were acceptable (equal to 0.584). Hence, all interactions were considered. The same was true for the YNL200C protein, where all interactions were considered, even though just 2 out of the 3 reported interactions fulfilled the above mentioned threshold. The third interactor of YNL200C protein was GAD1, which interacts with high confidence with the *YLR155C*-encoded product which is involved in oxidative stress. The latter information was relevant to the present work where FA-induced stress has been studied.
