# Supplementary material for: Yeast Biological Networks Unfold the Interplay of Antioxidants, Genome and Phenotype, and Reveal a Novel Regulator of the Oxidative Stress Response
Source: PLoS One. 2010 Oct 25;5(10):e13606. doi: 10.1371/journal.pone.0013606 (PMC2963615; doi:10.1371/journal.pone.0013606)
Supplement: Table S4 — Statistically significant protein complexes of the primary FA-specific network predicted by the MCODE clustering algorithm. (0.18 MB PDF) [file pone.0013606.s004.pdf]

Complex Rank	Score	No. Nodes	No. Edges	Cluster Members	Functional Enrichment (GO Annotation)	Total No. Nodes reported in GO Annotation	Corrected *p*-value
1	21,094	53	1118	RSM27, TPK1, TPK2, BCY1, TPK3, MRPL24, MRPL35, MRPL36, MRPL16, MRPL17, MRPL15, MRPL13, MRPL20, **IMG2**, **IMG1**, **MRPL28**, NAM9, MRP21, MRP1, **RSM19**, MRP13, RSM18, RSM26, RSM10, RSM22, RSM24, RSM25, RSM23, MRP51, MRPS8, MRPS5, EHD3, MRPS9, MAM33, MRP4, MRP7, YML6, MRP49, MHR1, MRPL3, MRPL1, MRPL4, MRPL7, **MRPL8**, MRPL9, MDM38, MRPS35, MRPS16, MRPS18, MRPS17, MRPS28, PET123, RSM7	**A)** Translation<br>**B)** Aerobic respiration<br>**C)** Ras protein signal transduction	50/53 (53)	1,3885 E-42<br>3,1554 E-3<br>6,7577 E-3
2	5,824	34	198	DBF2, RBG2, YGL157W, YMR090W, YJL200C, **YKL187C**, **FMP43**, SPG1, YJL103C, **YMR206W**, SPG4, TMA10, YER067W, CMK1, **YFR017C**, FMP16, NOP13, YGR067C, MOB1, SIC1, SWE1, CDH1, CDC6, CDC5, CDC20, CLB2, YBR138C, CDC28, MKK2, BCK1, SLT2, STE11, HSP82, NET1	**A)** Protein amino acid phosphorylation<br>**B)** Regulation of progression through the cell cycle	15/31 (34)	2,6232 E-7<br>3,9116 E-5
3	4,833	12	58	RCK1, ROD1, SDS22, APC4, SAK1, REG1, RAD30, **SIP2**, SIP1, GAL83, SNF4, SNF1	**A)** Protein amino acid phosphorylation<br>**B)** Regulation of biological process	9/12 (12)	1,4614 E-8<br>5,9611 E-5
4	4,833	6	29	ACT1, LSB3, SLA2, YSC84, SLA1, LAS17			
5	4,7	10	47	SUR4, GSF2, IFA38, SUR2, FEN1, GAS3, YET1, PHO86, PHO88, CHS7			
6	4,615	39	180	**MRPL23**, IVY1, GND1, RPO26, IWR1, ASR1, RPC10, TAF14, SRB5, SRB4, RPB5, RPB4, RPB7, RPB2, RPB11, RPB3, RPB10, RPB8, SPT5, SPT4, DST1, SET2, TFG1, RPB9, TFG2, RPO21, SSD1, RVS161, **AIM3**, IRE1, PAH1, YCK2, VIP1, YCK1, ALY2, RVS167, RSP5, YLH47, MRPL10	**A)** Transcription<br>**B)** Macromolecule metabolic process<br>**C)** Membrane organization and biogenesis	32/37 (39)	3,3904 E-13<br>1,5491 E-4<br>1,2779 E-2
7	4,5	10	45	URA2, AAT1, ADE12, **YLR155C**, AAT2, DPS1, ARG1, GAD1, HOM3, MSD1	**A)** Amino acid metabolic process<br>**B)** Biosynthetic process	7/10 (10)	1,9699 E-7<br>8,2161 E-5
8	3,667	9	33	GUT2, UGA2, UGA1, DCS2, MDH2, APE3, **PLB2**, YHR033W, YMR086W	Dicarboxylic acid metabolic process	2/9 (9)	8,4803 E-3
9	3,5	4	14	**POT1**, PEX18, PEX13, PEX7	Protein import into peroxisome matrix	3/4 (4)	2,0175 E-6
10	3,2	5	16	FUS3, STE5, RAD53, RAD9, MNR2			
11	3,133	15	47	SNG1, YLL056C, AZR1, PDR3, YIM1, YBT1, QDR2, MNT3, YMR102C, PDR1, YRR1, YPL088W, YOR1, TAP42, TOR2			
12	3	2	6	MEK1, RED1			
13	2,75	4	11	PCL10, MCK1, GSY2, GLC7			
14	2,667	12	32	SPC1, MST28, AGP1, CAN1, CSG2, SRP102, YPC1, GPI8, ALG1, PPA1, COS8, SNL1			
15	2,667	3	8	DCP2, LSM3, PAT1			
16	2,5	2	5	PHO81, PHO80			
17	2,45	20	49	AME1, NCP1, SAP185, SHS1, ISR1, RPG1, GTS1, ROM2, CDC19, KCC4, YNR047W, KSP1, SFB3, BCP1, NPL3, PAM1, PAN3, PCL1, UGP1, PHO85			

Statistically significant protein complexes of the primary FA-specific network predicted by the MCODE algorithm (score >2 and at least 4 edges). Proteins indicated by *bold* and *higher font letters* are members of the original gene list, while when these are highlighted in *light blue*, there exists no functional annotation for the corresponding genes. MCODE complexes marked by *light blue* include proteins encoded by genes being present in the original gene list, while complexes in *light grey* do not contain such proteins. For the subsequent analysis steps, only MCODE complexes 1-3 and 6-9 (*light blue* highlighted) have been used to derive the so-called ACTMOD network. The functional annotation of the identified MCODE complexes has been studied in terms of biological process (BP) over-representation by applying the BiNGO plug-in of Cytoscape. The annotation significance for these complexes is reported by means of corrected *p*-values, while this analysis was performed only for complexes 1-3 and 6-9. The 7th column of the table shown above indicates how many proteins (nodes) are included in the reported functional enrichment for a given complex, while the total number of complex members is shown in parenthesis (same number as in the 3rd column). BiNGO could not recognize some protein names in two cases (complex 2 and 6), hence at the 7th column the number of nodes written in the parenthesis is higher than the one written directly after the “/” symbol.
