# Supplementary material for: Yeast Biological Networks Unfold the Interplay of Antioxidants, Genome and Phenotype, and Reveal a Novel Regulator of the Oxidative Stress Response
Source: PLoS One. 2010 Oct 25;5(10):e13606. doi: 10.1371/journal.pone.0013606 (PMC2963615; doi:10.1371/journal.pone.0013606)
Supplement: Table S5 — Sub-cellular localization (Cerebral) and functional enrichment (BiNGO) analyses of the ACTMOD network. (0.52 MB PDF) [file pone.0013606.s005.pdf]

Application of the Cerebral and BiNGO plug-ins for the ACTMOD network

In order to create a more pathway-like view of the ACTMOD network, the Cerebral plug-in of Cytoscape has been used. This plug-in needs node subcellular localization information to be imported in Cytoscape by the user, hence in the present study, such information for the ACTMOD network was collected by using the GENECODIS functional annotation tool and the SGD database. GENECODIS has been chosen for this purpose, as it reports cellular component (CC) enrichment classes by taking into consideration the co-occurrence probability of a protein group at a given cellular compartment. However, in the GENECODIS result sheet (*see* Table 1), not all 167 nodes of the ACTMOD network were included. Therefore, in order to assign a CC class to every ACTMOD node, relevant information for these 25 nodes was retrieved from the SGD database (*see* Table 2).

In general, the localization results included five common cellular compartments, while 4 localization groups (loci A, B, C and D) have been created, each representing nodes with more than one assigned CC classes (2 or 3). Each node of this kind was included in A, B, C or D group, if the dual or triple CC assignment was identical or closely related with that of another node already belonging to one of the four defined groups. In detail, these groups were defined as follows: Loci A, proteins found in either cytoplasm or nucleus; Loci B, proteins being translocated between budle neck and another cellular compartment (like the spindle pole body, nucleus or plasma membrane); Loci C, proteins that can be found in cytoplasm and two other components (cell wall or plasma membrane and nucleus or mitochondrion); Loci D, proteins localized at mitochondrion and cytoplasm or mitochondrion. A summary of the CC information imported in Cytoscape to create the Cerebral view of the ACTMOD network is given in Figure 1, while the Cerebral view itself is shown in Figure 2.

Mor3eover, the functional annotation enrichment of the ACTMOD network has been studied in terms of biological process (BP) over-representation by applying the BiNGO plug-in of Cytoscape. The annotation significance for the identified classes was reported by means of corrected *p*-values, while the analysis was performed without dissecting the network into smaller interconnecting topological groups (for instance, as those result by applying the “circular layout” in Cytoscape) (*see* Table 3).

No. Nodes of the ACTMOD Network	Reference	*p* -value	Proteins localized in a given Cellular Component (CC)	GENECODIS: Cellular Component (CC) Co-occurrence Enrichment
26 (167)	34 (6194)	3,25E-35	RSM10, RSM24, MRPS28, MRP1, MRP21, MRPS9, MRPS5, RSM23, MRP13, MRPS35, RSM27, RSM26, RSM7, RSM22, PET123, RSM25, MRPS8, MRPS17, MRPS18, NAM9, RSM19, MRP51, MRPS16, MRP2, MRP4, RSM18	**mitochondrial small ribosomal subunit**
24 (167)	43 (6194)	2,09E-27	IMG1, IMG2, MRPL15, MRPL4, YDR115W, MRPL1, MRPL7, MRPL35, MRPL28, MRPL16, MRPL36, MRPL9, MRPL8, MRP49, MRPL13, MRPL20, MRPL23, YML6, MRPL3, MRPL24, MRPL10, MRPL17, MRP7, YPL183W-A	**mitochondrial large ribosomal subunit**
12 (167)	13 (6194)	1,26E-18	**RPO21**, RPB7, **RPB5**, RPB9, **RPB4**, RPB11, RPB2, **RPB10**, **RPB8**, RPB3, **RPO26**, **RPC10**	**DNA-directed RNA polymerase II, core complex**
5 (167)	5 (6194)	1,34E-08	**RPB5**, **RPB10**, **RPB8**, **RPO26**, **RPC10**	**DNA-directed RNA polymerase II, core complex | DNA-directed RNA polymerase III complex | DNA-directed RNA polymerase I complex**
3 (167)	3 (6194)	1,93E-06	**TPK1**, **TPK3**, **TPK2**	**cytoplasm | cAMP-dependent protein kinase complex**
3 (167)	3 (6194)	1,93E-05	TFG2, TFG1, **TAF14**	**transcription factor TFIIF complex**
3 (167)	8 (6194)	9,76E-04	**DBF2**, **MOB1**, **CLB2**	**spindle pole body | bud neck**
3 (167)	11 (6194)	2,71E-03	**SWE1**, **CDC5**, **CLB2**	**nucleus | bud neck**
3 (167)	16 (6194)	8,32E-03	APC4, CDC20, **CDH1**	**anaphase-promoting complex**
3 (167)	17 (6194)	9,91E-03	**SIP2**, **SNF4**, **RSP5**	**cytoplasm | plasma membrane**
3 (167)	20 (6194)	1,57E-02	SRB5, **TAF14**, SRB4	**mediator complex**
6 (167)	88 (6194)	3,09E-02	**DBF2**, **SWE1**, **MOB1**, **CDC5**, **YCK2**, **CLB2**	**bud neck**
41 (167)	1302 (6194)	1,50E-01	CMK1, YFR017C, DPS1, AAT2, **SIC1**, **TMA10**, STE11, VIP1, **IWR1**, REG1, TCP1, SSD1, **SNF1**, YBR138C, **CDC28**, **SIP2**, **YGL157W**, **SNF4**, **CDH1**, RBG2, **TPK1**, **RPB4**, **URA2**, **SDS22**, **TPK3**, **BCY1**, **YAP1**, CLU1, YMR090W, GAD1, ADE12, HSP82, **TPK2**, **ASR1**, **CLB2**, **SLT2**, **GND1**, HOM3, **YER067W**, **RSP5**, SAK1	**cytoplasm**
8 (167)	199 (6194)	1,68E-01	HXT15, **SIP2**, **SNF4**, ROD1, **YCK2**, HXT5, YCK1, **RSP5**	**plasma membrane**
4 (167)	100 (6194)	2,84E-01	**MDM38**, CYT1, COX11, **YLH47**	**mitochondrial inner membrane**
16 (167)	507 (6194)	2,91E-01	**SIC1**, **TMA10**, **IWR1**, **SNF1**, **CDC28**, **YGL157W**, **SNF4**, **CDH1**, **RPB4**, **SDS22**, **BCY1**, **YAP1**, **ASR1**, **CLB2**, **SLT2**, **YER067W**	**nucleus | cytoplasm**
3 (167)	71 (6194)	3,00E-01	**MHR1**, **SNF1**, **SPT5**	**nucleus | mitochondrion**
4 (167)	114 (6194)	3,70E-01	**SNF1**, **URA2**, **GND1**, **RSP5**	**cytoplasm | mitochondrion**
18 (167)	668 (6194)	5,39E-01	**RPO21**, EHD3, FMP16, **MHR1**, **SNF1**, SPG1, FMP43, YJL200C, **URA2**, YKL187C, AAT1, **MDM38**, IDH2, **SPT5**, MSD1, **YLH47**, **GND1**, **RSP5**	**mitochondrion**
4 (167)	193 (6194)	7,71E-01	SEC53, ATG1, ARG1, ARC35	**cytosol**
24 (167)	1120 (6194)	9,17E-01	MLH2, **SIC1**, **TMA10**, **IWR1**, **MHR1**, **SNF1**, **CDC28**, **YGL157W**, **SNF4**, **CDH1**, SPT4, **SWE1**, SET2, **RPB4**, **SDS22**, **BCY1**, **SPT5**, **YAP1**, **CDC5**, **ASR1**, **CLB2**, **SLT2**, GAL83, **YER067W**	**nucleus**

Table 1. Cellular component (CC) co-occurrence enrichment of the ACTMOD network, as this resulted by applying the GENECODIS tool. Nodes highlighted in *bold* represent proteins that belong to more than one CC classes.

ACTMOD Network Nodes	SGD: Cellular Component (CC)
SDH4	**mitochondrial inner membrane**
KRE2	**cytoplasm**
SEC10	**cytoplasm**
ALY2	**cytoplasm**
SIP1	**vacuole**
IVY1	**vacuole**
RVS161	**plasma membrane**
RVS167	**plasma membrane**
CDC6	**nucleus**
IRE1	**nucleus**
NET1	**nucleus**
RAD30	**nucleus**
DST1	**nucleus**
NOP13	**nucleus**
BCK1	**plasma membrane**
MKK2	**plasma membrane**
YLR155C	**cell wall-cytoplasm-nucleus**
AIM3	**unknown**
YL103C	**unknown**
MET8	**unknown**
YMR206W	**unknown**
YGR067C	**unknown**
RCK1	**unknown**
SPG4	**unknown**
MAM33	**mitochondrion**

Table 2. Subcellular localization of some ACTMOD network nodes according to SGD database.

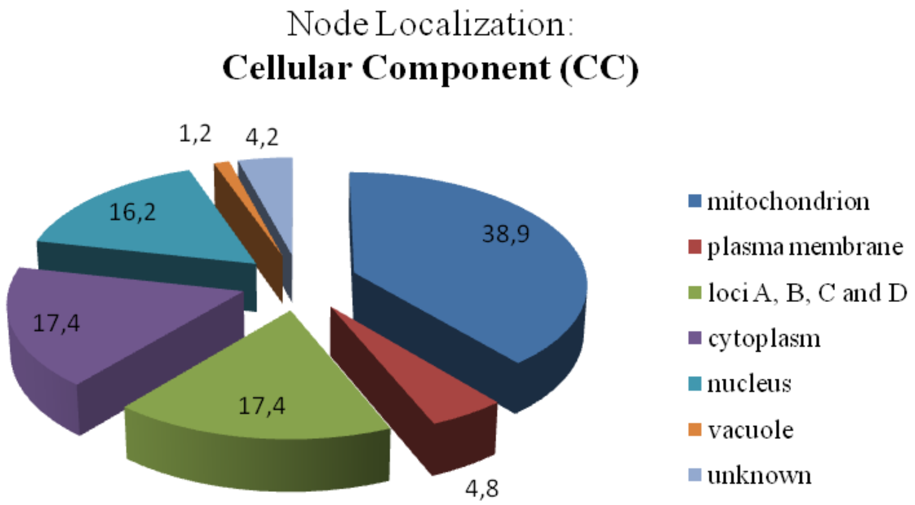

Figure 1. Summary of the ACTMOD node subcellular localization. For each component, the corresponding percentage of ACTMOD nodes is provided.

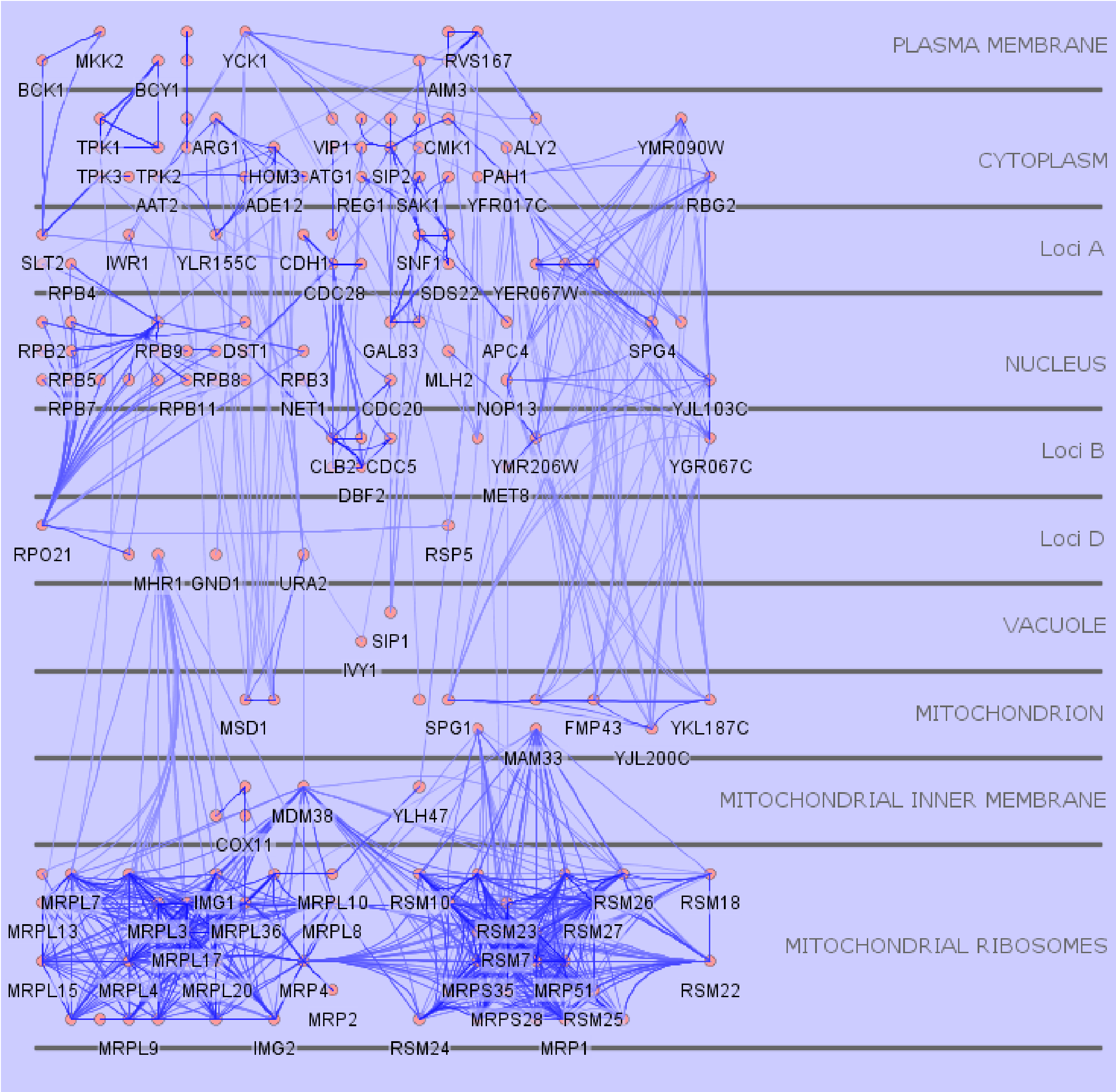

Figure 2. Cerebral view of the ACTMOD network created by combining GENECODIS- and SGD-derived subcellular localization information.

Protein Groups Enriched for a given Biological Process (BP)	Functional Enrichment: Biological Process (BP)	Total No. Nodes reported in GO Annotation	Corrected *p* -value
MRPL17, MRPL16, MRPL10, MRP21, RSM18, CLU1, MRP51, MRPS35, MRPL4, PET123, MRPS5, RSM25, MRPL35, MRPL23, MRP4, MRPS17, MRPS16, RSM23, MRPL15, MRPL24, MRPS18, IMG2, MRPL7, MRPS8, MRPL13, MRPL1, MRPL8, MRPS28, MRPL9, RSM27, RSM7, MRPS9, RSM10, MRP2, MRP7, MRPL28, MRP49, MRPL36, NAM9, RSM19, YML6, MRP13, MRPL20, RSM24, RSM26, MSD1, RSM22, DPS1, YPL183W-A, MRP1, MRPL3, IMG1	**Translation**	52/162 (167)	5,51E-19
CDC5, MOB1, YCK1, SNF1, SIP2, TPK1, CMK1, CDC28, MKK2, SAK1, RCK1, SNF4, GAL83, STE11, DBF2, SIP1, YCK2, BCK1, SLT2, TPK3, TPK2, IRE1	**Protein Amino Acid Phosphorylation**	22/162 (167)	1,22E-12
SDH4, MAM33, MRPS17, YDR115W, IDH2, PAH1, COX11, MRPL1, MRPS35, RSM24	**Aerobic Respiration**	10/162 (167)	3,11E-03
NET1, YAP1, RPC10, RPB7, IWR1, SPT4, SRB5, RPB10, RPB5, RPB2, SPT5, REG1, TFG2, RPB11, RPO21, CDC28, RPB9, RPB3, SNF4, RPO26, RPB4, SET2, TFG1, SRB4, DST1, TAF14, RSP5, RPB8	**Transcription**	28/162 (167)	1,48E-02
GAD1, IDH2, YLR155C, URA2, AAT2, ARG1	**Glutamine Family Amino Acid Metabolic Process**	6/162 (167)	1,61E-02
CLB2, SIC1, MOB1, SWE1, APC4, NET1, CDH1, CDC28, RCK1, VIP1, CDC20	**Regulation of Progression through the Cell Cycle**	11/162 (167)	4,80E-02
FMP43, FMP16, YMR090W, YJL200C, YJL103C, RBG2, YFR017C, YER067W, YGL157W, SPG4, YMR206W, YGR067C, TMA10, SPG1, YKL187C	**Unknown (FMP43-Complex)**	15/167	NA
ALY2, HXT5, AIM3, YBR138C, HXT15, ROD1	**Unknown (single or paired nodes)**	6/167	NA

Table 3. Functional annotation of the ACTMOD network, according to the BiNGO plug-in of Cytoscape. The 1st column indicates the members of each assigned biological process (BP) enrichment, the 2nd column shows the actual BP class, the 3rd column indicates how many proteins (nodes) are included in each class, while the total number of this class members is shown in parenthesis, and the 4th column of the table shown above reports the significance of each assigned BP enrichment by means of corrected p-values. BiNGO could not recognize some protein names, hence at the 3rd column, the number of nodes written in the parenthesis is higher than the one written directly after the “/” symbol. Also, there were 21 nodes that were not included in any reported BP enrichment, and these nodes could be topologically defined in two groups: one consisting of 15 nodes and representing the FMP43-related protein complex, and another consisting of topologically single or coupled nodes of the ACTMOD network.
