# Supplementary material for: Yeast Biological Networks Unfold the Interplay of Antioxidants, Genome and Phenotype, and Reveal a Novel Regulator of the Oxidative Stress Response
Source: PLoS One. 2010 Oct 25;5(10):e13606. doi: 10.1371/journal.pone.0013606 (PMC2963615; doi:10.1371/journal.pone.0013606)
Supplement: Table S6 — List of proteins participating in the FMP43 complex and their human orthology. (0.07 MB PDF) [file pone.0013606.s006.pdf]

**Yeast gene coding for a member of FMP43 complex**	**Human gene (orthologue)**	**Protein Description/UniProt Entry**
YER067W	No	NA
TMA10	No	NA
YJL200C	*ACO2*	Aconitase (Q71UF1)
NOP13	No	NA
YJL103C	No	NA
YMR206W	No	NA
YMR090W	No	NA
SPG4	No	NA
YFR017C	No	NA
FMP16	No	NA
YGL157W	No	NA
RBG2	*DRG2*	Developmentally-regulated GTP-binding protein 2 (P55039)
SPG1	No	NA
YGR067C	No	NA
YKL187C	No	NA

Proteins participating in the FMP43-related complex	SGD Description
YER067W (RGI1)	Putative protein of unknown function; green fluorescent protein (GFP)-fusion protein localizes to the cytoplasm and nucleus; YER067W is not an essential gene; protein abundance is increased upon intracellular iron depletion
TMA10	Protein of unknown function that associates with ribosomes
YJL200C (ACO2)	Putative mitochondrial aconitase isozyme; similarity to Aco1p, an aconitase required for the TCA cycle; expression induced during growth on glucose, by amino acid starvation via Gcn4p, and repressed on ethanol
NOP13	Nucleolar protein found in preribosomal complexes; contains an RNA recognition motif (RRM)
YJL103C (GSM1)	Putative zinc cluster protein of unknown function; proposed to be involved in the regulation of energy metabolism, based on patterns of expression and sequence analysis
YMR206W	Putative protein of unknown function; YMR206W is not an essential gene
YMR090W	Putative protein of unknown function with similarity to DTDP-glucose 4,6-dehydratases; GFP-fusion protein localizes to the cytoplasm; up-regulated in response to the fungicide mancozeb; not essential for viability
SPG4	Protein required for survival at high temperature during stationary phase; not required for growth on nonfermentable carbon sources
YFR017C	Putative protein of unknown function; green fluorescent protein (GFP)-fusion protein localizes to the cytoplasm and is induced in response to the DNA-damaging agent MMS; YFR017C is not an essential gene
FMP16	Putative protein of unknown function; proposed to be involved in responding to conditions of stress; the authentic, non-tagged protein is detected in highly purified mitochondria in high-throughput studies
YGL157W	Oxidoreductase, catalyzes NADPH-dependent reduction of the bicyclic diketone bicyclo[2.2.2]octane-2,6-dione (BCO2,6D) to the chiral ketoalcohol (1R,4S,6S)-6-hydroxybicyclo[2.2.2]octane-2-one (BCO2one6ol)
RBG2 (GIR1)	Protein with similarity to mammalian developmentally regulated GTP-binding protein
SPG1	Protein required for survival at high temperature during stationary phase; not required for growth on nonfermentable carbon sources; the authentic, non-tagged protein is detected in highly purified mitochondria in high-throughput studies
YGR067C	Putative protein of unknown function; contains a zinc finger motif similar to that of Adr1p
YKL187C	Putative protein of unknown function; the authentic, non-tagged protein is detected in a phosphorylated state in highly purified mitochondria in high-throughput studies
