# Supplementary material for: Yeast Biological Networks Unfold the Interplay of Antioxidants, Genome and Phenotype, and Reveal a Novel Regulator of the Oxidative Stress Response
Source: PLoS One. 2010 Oct 25;5(10):e13606. doi: 10.1371/journal.pone.0013606 (PMC2963615; doi:10.1371/journal.pone.0013606)
Supplement: Table S7 — Functional and structural characterization of the FMP43 protein using diverse bioinformatics tools and prediction servers (ProtFun, TMHMM, NetNES, SignalP, TargetP, NetPhosYeast, NetAcet and YinOYang servers). (0.03 MB PDF) [file pone.0013606.s007.pdf]

ProtFun 2.2

		Prob	Odds
**Functional category**	Energy metabolism	0,349	3,882
**Gene Ontology category**	Structural protein	0,117	4,193

The ProtFun 2.2 server produces *ab initio* predictions of protein function from sequence. The method queries a large number of other feature prediction servers to obtain information on various post-translational and localizational aspects of the protein, which are integrated into final predictions of the cellular role, enzyme class (if any), and selected Gene Ontology categories of the submitted sequence.

NetNES 1.1

No

NetNES 1.1 server predicts leucine-rich nuclear export signals (NES) in eukaryotic proteins using a combination of neural networks and hidden Markov models.

SignalP 3.0

No -- Non-secretory protein

SignalP 3.0 server predicts the presence and location of signal peptide cleavage sites in amino acid sequences from different organisms: Gram-positive prokaryotes, Gram-negative prokaryotes, and eukaryotes. The method incorporates a prediction of cleavage sites and a signal peptide/non-signal peptide prediction based on a combination of several artificial neural networks and hidden Markov models.

NetPhosYeast 1.0

# Sequence	# x	Context	Score	Kinase	Answer
# YGR243W	2 S	---MSASAF	0.833	main	YES
# YGR243W	4 S	-MSASAFNF	0.777	main	YES
# YGR243W	16 S	RFWNSETGP	0.355	main	.
# YGR243W	18 T	WNSETGPKT	0.219	main	.
# YGR243W	22 T	TGPKTVHFW	0.119	main	.
# YGR243W	29 T	FWAPTLKWG	0.053	main	.
# YGR243W	50 S	VEKVSGAQN	0.466	main	.
# YGR243W	56 S	AQNLSLLAT	0.459	main	.
# YGR243W	60 T	SLLATALIW	0.183	main	.
# YGR243W	65 T	ALIWTRWSF	0.071	main	.
# YGR243W	68 S	WTRWSFVIK	0.387	main	.
# YGR243W	80 S	YLLASVNFF	0.115	main	.
# YGR243W	88 T	FLGCTAGYH	0.081	main	.
# YGR243W	94 T	GYHLTRIAN	0.042	main	.
# YGR243W	106 S	RNGDSFKQV	0.616	main	YES
# YGR243W	119 T	IKGETPAAV	0.852	main	YES
# YGR243W	128 T	AAKQTASTS	0.244	main	.
# YGR243W	130 S	KQTASTSMN	0.551	main	YES
# YGR243W	131 T	QTASTSMNK	0.377	main	.
# YGR243W	132 S	TASTSMNKG	0.488	main	.
# YGR243W	140 T	GVIGTNPPI	0.200	main	.
# YGR243W	145 T	NPPITH---	0.365	main	.

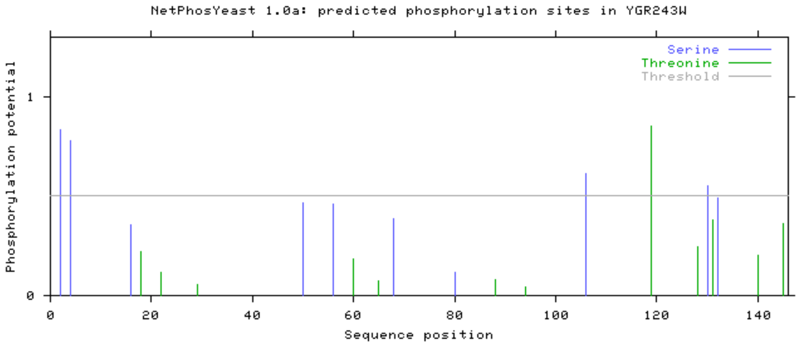

NetPhosYeast 1.0 server predicts serine and threonine phosphorylation sites in yeast proteins.

NetAcet 1.0

# Sequence	#	Context	Score	Acetylation
# ---				
YGR243W	2 S	--MSASA	0.515	YES
YGR243W	3 A	-MSASAF	0.478	.

NetAcet 1.0 server predicts substrates of N-acetyltransferase A (NatA). The method was trained on yeast data but, as mentioned in the article describing the method, it obtains similar performance values on mammalian substrates acetylated by NatA orthologs.

TargetP 1.1

No

TargetP 1.1 predicts the subcellular location of eukaryotic proteins. The location assignment is based on the predicted presence of any of the N-terminal presequences: chloroplast transit peptide (**cTP**), mitochondrial targeting peptide (**mTP**) or secretory pathway signal peptide (**SP**).

For the sequences predicted to contain an N-terminal presequence a potential cleavage site can also be predicted.

TMHMM 2.0

Not a TM protein

YGR243W Number of predicted TMHs: 0

YGR243W Exp number of AAs in TMHs: **14.48846**

YGR243W Exp number, first 60 AAs: 2.27829

YGR243W Total prob of N-in: 0.17677

YGR243W TMHMM2.0 outside 1 146

Should be **>18** to be a TM protein

Prediction of transmembrane helices in proteins

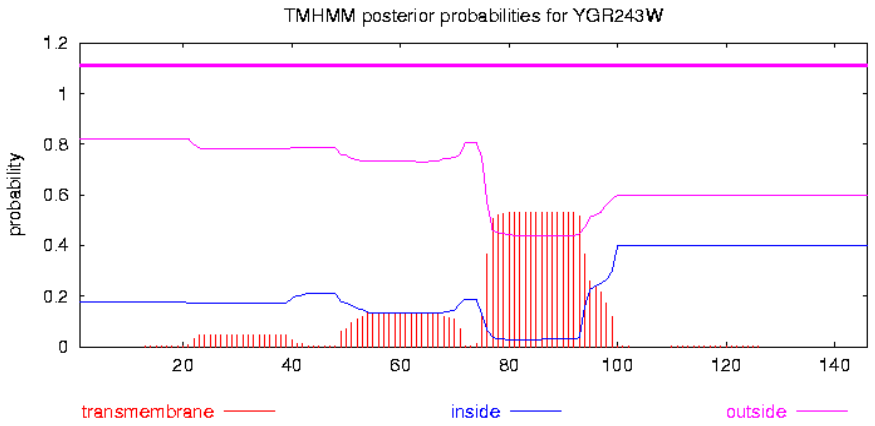

YinOYang 1.2

5 O-GlcNAc sites

SeqName	Residue	O-GlcNAc result	Potential	Thresh. (1)	Thresh. (2)
YGR243W	2 S	+	0.4231	0.3681	0.4465
YGR243W	50 S	+++	0.5631	0.3628	0.4394
YGR243W	128 T	+++	0.5697	0.3698	0.4489
YGR243W	131 T	+	0.4682	0.3863	0.4711
YGR243W	145 T	+++	0.5671	0.3477	0.4190

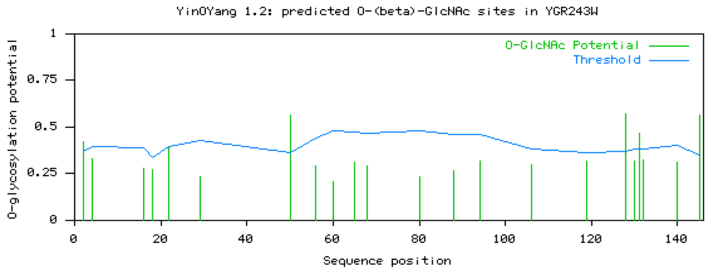

The YinOYang WWW server produces neural network predictions for O-ß-GlcNAc attachment sites in eukaryotic protein sequences. This server can also use NetPhos, to mark possible phosphorylated sites and hence identify "Yin-Yang" sites.

**Prediction Server**		**FMP43 Protein Predictions**
**ProtFun 2.2**	**Protein Function+Structure**	Energy Metabolism, Structural Protein
**TMHMM 2.0**		Not a TM protein

Prediction Server		FMP43 Protein Predictions
**NetNES 1.1**	**Protein Sorting**	No leucine-rich nuclear export signals (NES)
**SignalP 3.0**		Non-secretory protein
**TargetP 1.1**		No N-terminal presequence for subcellular localization

Prediction Server		FMP43 Protein Predictions
**NetPhosYeast 1.0**	**Post-translational Modifications**	5 phosphorylation sites (4 Ser, 1Thr)
**NetAcet 1.0**		1 acetylation site
**YinOYang 1.2**		5 O-GlcNAc sites
