# Supplementary material for: Yeast Biological Networks Unfold the Interplay of Antioxidants, Genome and Phenotype, and Reveal a Novel Regulator of the Oxidative Stress Response
Source: PLoS One. 2010 Oct 25;5(10):e13606. doi: 10.1371/journal.pone.0013606 (PMC2963615; doi:10.1371/journal.pone.0013606)
Supplement: Table S8 — The gene deletion and over-expression strategies followed for the construction of the relevant yeast strains used in the present study, namely CEN.PK 113-5D/Î¿fmp43 and CEN.PK 113-5D/Î¿fmp43-pRS426-BRP44. (0.61 MB PDF) [file pone.0013606.s008.pdf]

A. Gene deletion strategy

The advanced cloning-free PCR-based allele replacement strategy (Erdeniz *et al.*, 1997) has been followed in the present study. The principle of this strategy is graphically depicted in *Figure 1a*. In the gene targeting experiment performed in the present study, the uracil auxotrophy (Ura$^-$) of CEN.PK 113-5D strain has been exploited. Thus, cells where the gene targeting substrate integrated correctly (*see lower left part of Figure 1b*) were easily detected, as these cells had obtained a new selectable feature, i.e., *URA3* gene (*URA3*; uracil gene from another yeast species, *K. lactis*), and were selected by plating on uracil deficient synthetic complete media (SC-Ura) in a positive selection procedure.

A complex gene targeting substrate has been used for the deletion of the *FMP43* gene, as after positive selection, the *URA3* marker has been stably integrated at the target site, and the strain couldn't thus be re-targeted by using the *URA*3 gene as a marker. Therefore, the *URA3* marker was flanked by a direct repeat in the complex gene targeting substrate (*see lower part of Figure 1a*). *In vivo* recombination between these two direct repeat individual units could eliminate the *URA3* gene from the genome (*see right part of Figure 1b*). After this event, called pop-out recombination**,** the strain becomes Ura$^-$ again, and can thus be further manipulated by a new round of *URA3*-based targeting.

Gene targeting substrates have been constructed based on PCR methods. In total, five individual pieces of DNA need to be fused to create the substrate. The fusions required to make the complete gene targeting substrates require PCR and adaptamer technology as well as *in vivo* homologous recombination (*see Figure 1*). The core part of the substrate, the *URA3* gene flanked by a direct repeat, is constant in all experiments. For that reason, a plasmid, pWJ1042 has been used that contains the central three-partite part of the gene targeting substrate. To construct the complete gene targeting substrate, the sequence upstream of the target site needs to be fused to one end of the core targeting substrate, and the sequence downstream of the target site needs to be fused to the other end of the core substrate.

(a)

(b)

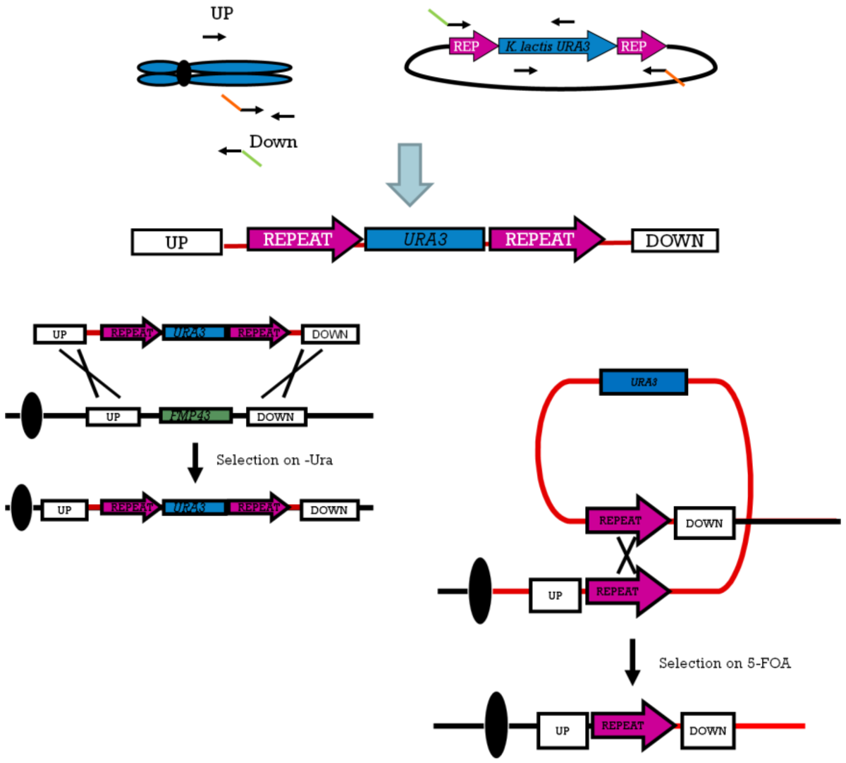

Figure 1. (a) The principle behind the cloning-free PCR-based allele replacement strategy; adaptamer technology applied to the two different DNA templates is depicted in the *upper part*, while the structure of the gene targeting substrate is shown in the *lower part*. (b) Homologous recombination in yeast cells during the gene targeting experiment; on the *left side* of the graph, the substrate integration and *FMP43* gene replacement is shown, while on the right side the pop-out recombination event is explained. The whole process constitutes the so-called two-step gene disruption method.

Adaptamer technology is a method to fuse two PCR DNA fragments by an additional round of PCR. Thus, large quantities of composite DNA fragments can be generated without any need for *E. coli* based cloning steps. An adaptamer is a primer that contains a short addition of a specific sequence at its 5’-OH end. Hence, if two different PCR fragments to be fused are generated by adaptamers that contain a complementary sequence addition in their 5’-OH ends, these fragments will have a common sequence at their ends, and eventually fusion in a subsequent round of PCR will be enabled.

Recombination between direct repeats is a rare event, and selection is required to identify the direct repeat recombinants. Negative selection was applied by using the 5-FOA (5-fluorouracil-6-carboxylic acid monohydrate; 5-fluororotic acid) chemical, which kills cells that contain a wild-type copy of *URA3*. As a result, *ura3* cells can grow in the presence of 5-FOA, whereas *URA3* cells die (*see Figure 2*).

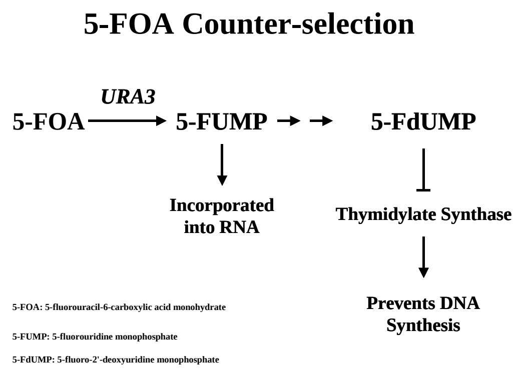

Figure 2. Selection of *ura3* cells mediated by 5-fluorouracil-6-carboxylic acid monohydrate (5-FOA). These cells manage to survive in the presence of this chemical, while *URA3* cells die. This happens because the *URA3* gene product, orotidine-5'-phosphate decarboxylase, converts 5-FOA molecule into the toxic compound 5-FUMP, and further metabolism turns this compound into the even more toxic 5-FdUMP. In contrast, *ura3* cells do not contain orotidine-5'-phosphate decarboxylase activity and they do not convert 5-FOA into any toxic substances.

During *FMP43* knockout mutation induction, several PCR fragments have been generated. Two fragments represent the upstream and downstream regions of *FMP43*, respectively, and two fragments represent the 5' 2/3 of *K. lactis URA3* and the 3' 2/3 of *k. lactis URA3*. The primer sets each consisted of one regular primer and one adaptamer. The PCR fragments flanking *FMP43* have been generated by using the reference strain CEN.PK 113-5D genomic DNA as a template. The *K. lactis URA3* fragments have been in both cases generated by using the plasmid pWJ1042 as template (laboratory stock). The sequences of the plasmid WJ1042, the genomic regions flanking *FMP43* gene, and the actual *FMP43* gene sequence together with the corresponding designed deletion primers are given below. Note that *K. lactis URA3* in pWJ1042 is flanked by a direct repeat, which as mentioned above, allows iterative gene targeting. Phusion™ High-Fidelity DNA Polymerase has been used for all PCR reactions performed during the gene deletion protocol.

Step 1: Creation of first set of PCR fragments

PCR Reaction 1	
DNA template	*S.cerevisiae FMP43* structural gene and flanking sequences
PCR Product Size	**350 bp**

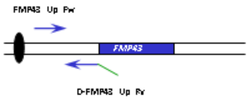

Amplification of the *FMP43* upstream region

PCR Reaction 2	
DNA template	*S.cerevisiae FMP43* structural gene and flanking sequences
PCR Product Size	**130 bp**

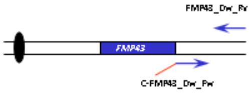

Amplification of the *FMP43* downstream region

PCR Reaction 3	
DNA template	*K.lactis URA3* structural gene and flanking sequences
PCR Product Size	**1265 bp**

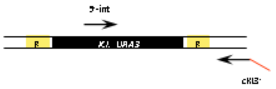

Amplification of the *URA3* downstream region

PCR Reaction 4	
DNA template	*K.lactis URA3* structural gene and flanking sequences
PCR Product Size	**884 bp**

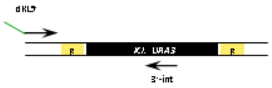

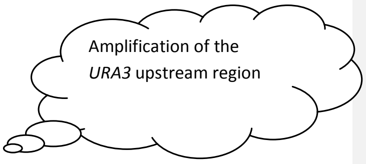

A sample PCR reaction for the creation of this first set of PCR products is given below.

5 μl	Sense primer (20 μM)
5 μl	Antisense primer (20 μM)
10 μl	Phusion HF Buffer
5 μl	dNTP mix (2.5 mM)
0.3 μl	Phusion Taq Polymerase (1u/μl)
1 μl	genomic DNA (0.5 μg/μl)
23.7 μl	H_2O
50 μl	

The PCR programs for all the reactions performed in this step are given below. The program shown on the left side was employed for PCR reactions 1 and 2, while the program displayed on the right side refers to PCR reactions 3 and 4. It should be noted that PCR product yield optimization and condition standardization have been performed separately for each designed PCR reaction through minor temperature and time interval changes.

98º C	30’’	
98º C	10’’	30 Cycles
61º C	10’’	30 Cycles
72º C	1’	30 Cycles
72º C	10’	
12º C	Pause	

98º C	30’’	
98º C	10’’	30 Cycles
65º C	10’’	30 Cycles
72º C	1’	30 Cycles
72º C	10’	
12º C	Pause	

Step 2: Agarose gel electrophoresis and purification of PCR fragments

Agarose electrophoresis gels were run (*see Appendix 3*) to purify desired DNA fragments for subsequent PCR reactions (5 and 6). After running the gel, the different bands were visualized and cut in pieces in order to collect the desired bands with a scalpel. This has been performed several times by placing the gel on a hand held low intensity UV-lamp, and avoid in this manner any DNA damage (pyrimidine dimers and strand breaks) induced in cases where the UV-light boxes are used instead. The cut agarose fragments were then transferred in an eppendorf tube, and the agarose mass was eventually determined in an appropriate balance.

The DNA gel purification steps followed during the entire gene deletion procedure contributed to a higher DNA quality of the final fragments transformed in yeast. The separation of DNA from agarose was enabled by using the QIAEX II Gel Extraction Kit™ from Qiagen**.** The kit contains a chemical that dissolves the agarose without harming the DNA and a matrix to which DNA can bind in the presence of high salt concentration. After DNA has been adsorbed to the matrix, it can be washed to remove agarose, EtBr and other contaminants. Finally, the DNA is eluted in a low-salt buffer. The gel band extraction protocol provided in the kit has been applied.

Step 3: Creation of second set of PCR fragments

PCR Reaction 5	
DNA template	PCR products 1 and 4
PCR Product Size	**1234 bp**

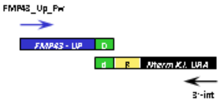

Fusion of the *FMP43* upstream region to the upstream *URA3* fragment

PCR Reaction 6	
DNA template	PCR products 2 and 3
PCR Product Size	**1395 bp**

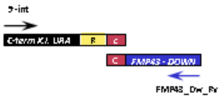

Fusion of the *FMP43* downstream region to the downstream *URA3* fragment

A sample PCR reaction for the creation of this second set of PCR products is given below. PCR products produced in previous steps (1,2,3,4) were combined in pairs: 1 with 4, and 2 with 3.

5 µl	Sense primer (20 µM)
5 µl	Antisense primer (20 µM)
10 µl	Phusion HF Buffer
5 µl	dNTP mix (2.5 mM)
0.3 µl	Phusion Taq Polymerase (1u/µl)
5 µl	PCR fragment from reaction 1 or 2
5 µl	PCR fragment from reaction 4 or 3
14.7 µl	H_2O
50 µl	

Temperature and time interval changes have been made until PCR product yield and conditions were optimal for either reaction. The core program for all fusion PCR reactions performed in this step was as follows.

98º C	30	
98º C	10’’	
52º C	5’	5
72º C	2’ 30’’	
98º C	10’’	
52º C	45’’	30
72º C	2’ 30’’	
72º C	5’	
12º	Pause	

Step 4: Agarose gel electrophoresis and purification of PCR fragments (as described above).

Step 5: Yeast transformation with the gene targeting substrate fragments

S. cerevisiae strain CEN.PK113-5D was co-transformed with the two created gene targeting substrate fragments. Cells were made competent for DNA uptake using a traditional lithium acetate treatment. A total amount of $2x10^7$cells/ml was transformed with an equimolar amount of the PCR products 5 and 6. The appropriate amount of fragments (approximately 500ng of each product) has been determined after product quantification in an appropriate gel visualization and analysis software provided by BioRad Laboratories, called QuantityOne. An appropriate transformation master mix was prepared, and in total, three reactions were designed per experiment. Positive and negative transformation controls have been included (2 out of 3 total reactions). The competency of the CEN.PK 113-5D cells was determined by transforming the cells with 1 μg pRS416 DNA provided by New England Biolabs Inc., whereas a mix with only MQ water (no DNA) has been prepared as a negative control of the transformation experiments. Transformants were selected for on synthetic dextrose agar plates without uracil (SC-Ura), while cross-selection on 5-FOA plates, as described before, was also needed.

Primer design using VectorNTI software and special considerations

The efficiency of gene targeting highly depends on the length of homologous sequence between the DNA to be inserted and the genomic target site. This parameter varies from organism to organism, but in yeast the efficiency of gene targeting is very low if the regions of homology are lower than 100 base pairs. In contrast, maximum efficiency is achieved when the regions of homology are ≥ 500 base pairs. Primers for amplification of the *FMP43* upstream region have been designed based on this fact. Initially, the genome position 500bp upstream the *FMP43* coding sequence (CDS) has been targeted for primer design. However, any suitable primer with desirable properties could be identified in this region, and eventually a primer targeting 299bp upstream of *FMP43* CDS has been chosen. For amplification of the *FMP43* downstream region, an overlap between the gene under study and *ARS733* gene had to be taken into consideration in the design process (*see Figure 3*). Autonomously replicating sequences (ARSs) function as chromosomal replication origins in *S. cerevisiae*, and play an essential role in chromosome maintenance (*www.yeastgenome.org/*). Therefore, in PCR reaction 2, only a small region at the 3' end of *FMP43* has been amplified –not the actual downstream region as indicated by the method- because of this gene overlap between *FMP43* and *ARS733,* and the need to keep intact the *ARS733* CDS. In case of *K. lactis URA3* gene, the corresponding primers for up- and downstream region amplification have been ordered from Sigma-Aldrich. A comprehensive list of primers used is given below. It should be noted that stop codon sequences have been flanked between the adaptamer and *FMP43* gene sequence that served as template for primer hybridization, in order to avoid any frame disruption potentially caused by the adaptamer sequences.

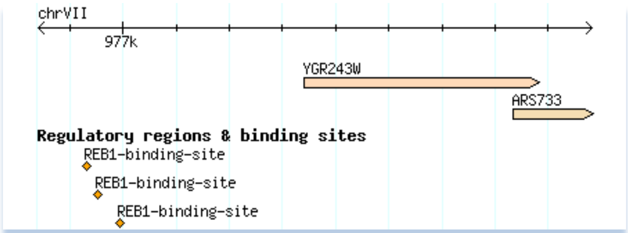

Figure 3. Overlapping between the *YGR243W/FMP43* and *ARS733* genes in *S. cerevisiae* genome.

The design of all PCR primers has been carried out in VectorNTI© Advance 10.3.0 software program from Invitrogen. SGD database was used for *FMP43* CDS and flanking (up- and downstream) region sequence retrieval. The following critical primer design settings have been established: melting temperature of the hybridizing regions between 58°C and 65°C (length set to 18 to 26bp), and GC-content 40-60%. The first three nucleotides from the 3'-end of each primer were set to be G or C, in order to induce efficient annealing of this end, and thereby ensure good priming for the DNA polymerase. The melting temperature was set to be of the highest importance '10'.

List of all primers used for *FMP43* gene deletion

Color code:

Primer part in **black** and adaptamer tag in **red**.

Stop codon sequence in light blue.

Deletion primers for *FMP43*:

FMP43_Up_Fw: **5'-CCAATCGGCTATTAACGGCTTTAC-3'**

D-FMP43_Up_Rv: **5'-**

GCAGGGATGCGGCCGCTGACTCAGGCAAAATTAAAAGCTGATGCTGAC-3'

C-FMP43_Dw_Fw: **5'-**

CCGCTGCTAGGCGCGCCGTGTGATTGCTAACTTTAGGATACGGAACGG-3'

FMP43_Dw_Rv: **5'- TGTGGATGCAGTTTGCTTTGCT-3'**

***K. lactis URA3* Primers:**

5'-int: **5'-CTTGACGTTCGTTCGACTGATGAGC-3'**

3'-int: **5'-GAGCAATGAACCCAATAACGAAATC-3'**

cKL3': **5'-CACGGCGCGCCTAGCAGCGGTAACGCCAGGGTTTTCCCAGTCAC-3'**

dKL5': **5'-GTCAGCGGCCGCATCCCTGCTTCGGCTTCATGGCAATTCCCG-3'**

Eventually, by using the group of primers presented above, the final size of the *FMP43* CDS that has been deleted is **259bp** in a total *FMP43* CDS size of **441 bp**. An overview of the primer design procedure followed is graphically provided in *Figure 4*.

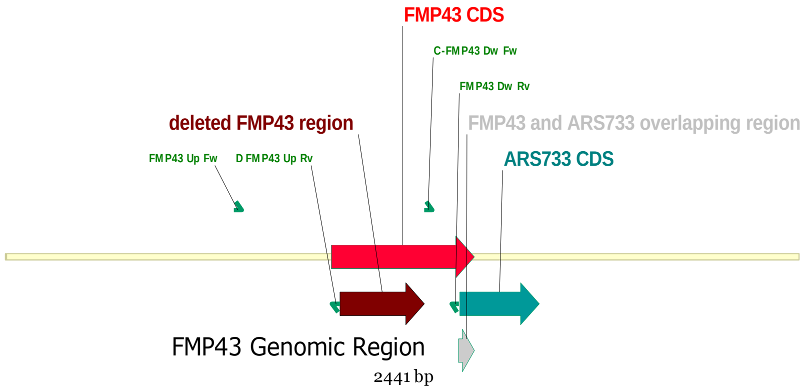

Figure 4. Overview of the primer design performed for *FMP43* gene deletion. The primers employed, the coding sequence (CDS) of *ARS733*, and the corresponding *FMP43* region deleted are illustrated by different colors for better inspection.

DNA sequences for *FMP43* targeting substrate construction

I) **<u>*YGR243W/FMP43*: 1 kb up- and downstream of the coding sequence (CDS)</u>**

**Color code**	
Black sequence	*FMP43* genomic sequences that flank the *FMP43* structural gene in *S. cerevisiae* genome
Red sequence	*FMP43* (structural gene)
Blue sequence	*ARS733* (structural gene)
**<u>Black sequence</u>**	Overlapping sequences of *FMP43* and *ARS733*

ATGGGAATAGTGCCTTAGCGTGACATCTTTTTCGCCTTGTTGTATCATCAGATGCAACACAATC
AATGCCTTATAAACAATGGTCCAGGCTGTATCAGACAACCTTATATCCAATGCGTGGGTAATCT
CTTGCAATCCTCTTGCGCTCGAAGTGCCCGAGAGAATAGGGTCCACATATTTTTGCTTTGGAGG
GGCCATCTTTATTTTGGTCGCCCCTTTAACCAACTTAGTATATAATGAAGACATTGTTTCACAAA
CACGCCTCTATATCTATTATAGGGGTAGCCGAGACAGTCGCGCTTGTATGCTTGATATTGTTACT
CTCGTAGATGTTAGCAAGCGTAGCAAATTGGTACTCCTGCTACAGATTTATTTTTTCTCCTTCTA
AAATTATAACTCAAGCAAGAAGCCCTAATAACAAGTTAACCAGACTAATAAGTCGACTTTTTCC
TCGAATGTAGAAAAGAAACTTTTCTACGCAGCTGCAAACTTAATCTATCCCTCCACCAAAGCAA
AATGAAAACAAAGCCATACTGGGAAAAATCTGAAAAAAAAAAATGGTAGGAGTAAAAGAAAAG
AAAAAATAAAGGTTACCCTGCAGTTTGGATAGTCGGGTAACATTTGGCCCTTTTCCTCCTTGATT
GGATATTATTACCCCGATTACCCCTCATCTTGGGAGTGCCCCGCTTTTATTTCTCCCGCCAATCG
GCTATTAACGGCTTTACGTCATTCCGTGGGCGGGTCAAGCGAGCCGCTCCCTGGTTTGGTCACG
CAAAACCGAAAGGCTCAAACAAAACTAAGGCCATCATATATATATATGCGGCTGCGTGCGTGT
ATTCTCCCGGATAATATGGTGCGTTGCAATTGGAGTATTGGAGAAAATTTTCTTTTCCCTTTCAT
TACGGCGGAAATACTTCATATAAAAAAAAAGAATACAATCAGTCTTTAAGACTATACGCATAA
GCATTCAAGACACATAGAAACACAAACCTATATTTTTA**ATG**TCAGCATCAGCTTTTAATTTTGC
CTTTAGAAGATTTTGGAATAGTGAAACAGGCCCTAAAACAGTACACTTCTGGGCCCCAACTTTG
AAGTGGGGGCTGGTCTTCGCAGGGCTAAATGATATTAAGAGGCCTGTTGAGAAGGTATCAGGA
GCACAAAATTTATCTTTATTAGCGACGGCACTGATTTGGACGCGTTGGTCGTTTGTCATCAAGC
CCAAGAACTATCTGTTAGCTTCCGTCAATTTTTTCCTGGGTTGCACTGCAGGCTACCATCTAACA
AGAATTGCTAACTTTAGGATACGGAACGGTGATTCTTTTAAACAGGTTATTCACTACATAATAA
AAGGGGAGACTCCTGCAGCCGTCGCAGCAAAGCAAACTGCATCCAC**ATCGATGAACAAAGGT**
GTGATCGGTACTAATCCGCCAATAACGCACTGATTACGTAAACGATAATATGTTCCTGAACT
CGCATTTTTTTAATGATTTTTTATGACCTCTTATATATTCTTTCATTATATAACCTCTATATTACA
TCAAAAGATGGAAAAATATAAAAAAATTAAAAAAAAAAAAAAGAAATTTTTTCCAAAAGAGT
ATATTTATATGTATGTATACATGTAGGGAAAA**TAA**GAAACTTTATTAATAGTAAAAAAGCATAT
ATACTTTATTATTAACTCTTTTGTTTTTCTCGAGAAGCTTAATTTTGGGTCAATTCAACGACCTTT
TTAGCAGCAGGATCTAATTCATCAAACGAATAAATCTTCACGCCTGACTTATTGATAATGTCGC
GGCCTTCTTCTACTTTGGTACCTTGCAAACGTGCCACAATGGGGACCCTAACTTCTAGTTCTCTG
GCGGCTTCTACCAGCCCCAGGGCAACATAGTCACATCTTACGATACCGCCGAAAATATTGACGA
AAATTGCATCTACGTTCTTATTGGATAAGATCAATTCGAAACCTTGTTTGATGGTCTCAGGGGT
GGCACCACCACCACAATCCAAAAAGTTCGCAGGATCGCCTCCATTTAATTTGATGACATCCATA
GTAGCCATAGCCAAACCAGCACCATTGACTAAACATCCAATGTTACCCTTCAACTTAACAAAAT
TCAAATCATACTTCTTTGCCTTAACTTCATCAGGATCTTCTTGTGATAAGTCCCTCCAGGAATAT
ATCTTTTCCTGTCTGAATGATGCGTTATCATCAAACCCAAATTTGGCGTCTGTACACATGATTTT

Comment [M1]: FMP43_Up_Fw:
5'CCAATCGGCTATTAACGGCTTTAC 3'

Comment [M2]: D-FMP43_Up_Rv:
5'GCAGGGATGCGGCCGCTGACTCAGGCAAA ATTAAAAGCTGATGCTGAC 3'

Comment [M3]: C-FMP43_Dw_Fw:
5'CCGCTGCTAGGCGCGCCGTGTGA TTGCTAACTTTAGGATACGGAACGG 3'

Comment [M4]: FMP43_Dw_Rv:
5' TGTGGATGCAGTTTGCTTTGCT 3'

GTGGGTTGGATCATGTTCAATTTCACTCAAAGGGTTAATCTCCACTTGTGTAGCATCCCTTTCCA
TGAATATTTTATACAAATTGGAAACAGCTTTTGCTGCCTCGTCTTGTGCATCGGGACTGAAACC
GAGACTCTTGGCAACATCCTTGGCCATTTGTGGGCTCAATCCCTTTGAAGTTTCAATTGAAAATT
TCTT

II) <u>pWJ1042/*K. lactis URA3* locus</u>

**Color code**	
Green sequence	Direct repeats that flank *URA3*
Red sequence	*URA3* (structural gene)
Blue sequence	Primer target sites
Black sequence	Plasmid backbone

GACGAAAGGGCCTCGTGATACGCCTATTTTTATAGGTTAATGTCATGATAATAATGGTTTCTTA
GGACGGATCGCTTGCCTGTAACTTACACGCGCCTCGTATCTTTTAATGATGGAATAATTTGGGA
ATTTACTCTGTGTTTATTTATTTTTATGTTTTGTATTTGGATTTTAGAAAGTAAATAAAGAAGGT
AGAAGAGTTACGGAATGAAGAAAAAAAAATAAACAAAGGTTTAAAAAATTTCAACAAAAAGC
GTACTTTACATATATATTTATTAGACAAGAAAAGCAGATTAAATAGATATACATTCGATTAACG
ATAAGTAAAATGTAAAATCACAGGATTTTCGTGTGTGGTCTTCTACACAGACAAGATGAAACA
ATTCGGCATTAATACCTGAGAGCAGGAAGAGCAAGATAAAAGGTAGTATTTGTTGGCGATCCC
CCTAGAGTCTTTTACATCTTCGGAAAACAAAAACTATTTTTTCTTTAATTTCTTTTTTTACTTTCT
ATTTTTAATTTATATATTTATATTAAAAAATTTAAATTATAATTATTTTTATAGCACGTGATGAA
AAGGACCCAGGTGGCACTTTTCGGGGAAATGTGCGCGGAACCCCTATTTGTTTATTTTTCTAAA
TACATTCAAATATGTATCCGCTCATGAGACAATAACCCTGATAAATGCTTCAATAATATTGAAA
AAGGAAGAGTATGAGTATTCAACATTTCCGTGTCGCCCTTATTCCCTTTTTTGCGGCATTTTGCC
TTCCTGTTTTTGCTCACCCAGAAACGCTGGTGAAAGTAAAAGATGCTGAAGATCAGTTGGGTGC
ACGAGTGGGTTACATCGAACTGGATCTCAACAGCGGTAAGATCCTTGAGAGTTTTCGCCCCGAA

GAACGTTTTCCAATGATGAGCACTTTTAAAGTTCTGCTATGTGGCGCGGTATTATCCCGTATTGA
CGCCGGGCAAGAGCAACTCGGTCGCCGCATACACTATTCTCAGAATGACTTGGTTGAGTACTCA
CCAGTCACAGAAAAGCATCTTACGGATGGCATGACAGTAAGAGAATTATGCAGTGCTGCCATA
ACCATGAGTGATAACACTGCGGCCAACTTACTTCTGACAACGATCGGAGGACCGAAGGAGCTA
ACCGCTTTTTTGCACAACATGGGGGATCATGTAACTCGCCTTGATCGTTGGGAACCGGAGCTGA
ATGAAGCCATACCAAACGACGAGCGTGACACCACGATGCCTGTAGCAATGGCAACAACGTTGC
GCAAACTATTAACTGGCGAACTACTTACTCTAGCTTCCCGGCAACAATTAATAGACTGGATGGA
GGCGGATAAAGTTGCAGGACCACTTCTGCGCTCGGCCCTTCCGGCTGGCTGGTTTATTGCTGAT
AAATCTGGAGCCGGTGAGCGTGGGTCTCGCGGTATCATTGCAGCACTGGGGCCAGATGGTAAG
CCCTCCCGTATCGTAGTTATCTACACGACGGGGAGTCAGGCAACTATGGATGAACGAAATAGA
CAGATCGCTGAGATAGGTGCCTCACTGATTAAGCATTGGTAACTGTCAGACCAAGTTTACTCAT
ATATACTTTAGATTGATTTAAAACTTCATTTTTAATTTAAAAGGATCTAGGTGAAGATCCTTTTT
GATAATCTCATGACCAAAATCCCTTAACGTGAGTTTTCGTTCCACTGAGCGTCAGACCCCGTAG
AAAAGATCAAAGGATCTTCTTGAGATCCTTTTTTTCTGCGCGTAATCTGCTGCTTGCAAACAAA
AAAACCACCGCTACCAGCGGTGGTTTGTTTGCCGGATCAAGAGCTACCAACTCTTTTTCCGAAG
GTAACTGGCTTCAGCAGAGCGCAGATACCAAATACTGTCCTTCTAGTGTAGCCGTAGTTAGGCC
ACCACTTCAAGAACTCTGTAGCACCGCCTACATACCTCGCTCTGCTAATCCTGTTACCAGTGGCT
GCTGCCAGTGGCGATAAGTCGTGTCTTACCGGGTTGGACTCAAGACGATAGTTACCGGATAAG
GCGCAGCGGTCGGGCTGAACGGGGGGTTCGTGCACACAGCCCAGCTTGGAGCGAACGACCTAC
ACCGAACTGAGATACCTACAGCGTGAGCTATGAGAAAGCGCCACGCTTCCCGAAGGGAGAAAG
GCGGACAGGTATCCGGTAAGCGGCAGGGTCGGAACAGGAGAGCGCACGAGGGAGCTTCCAGG
GGGAAACGCCTGGTATCTTTATAGTCCTGTCGGGTTTCGCCACCTCTGACTTGAGCGTCGATTTT
TGTGATGCTCGTCAGGGGGGCGGAGCCTATGGAAAAACGCCAGCAACGCGGCCTTTTTACGGT
TCCTGGCCTTTTGCTGGCCTTTTGCTCACATGTTCTTTCCTGCGTTATCCCCTGATTCTGTGGATA
ACCGTATTACCGCCTTTGAGTGAGCTGATACCGCTCGCCGCAGCCGAACGACCGAGCGCAGCG
AGTCAGTGAGCGAGGAAGCGGAAGAGCGCCCAATACGCAAACCGCCTCTCCCCGCGCGTTGGC
CGATTCATTAATGCAGCTGGCACGACAGGTTTCCCGACTGGAAAGCGGGCAGTGAGCGCAACG
CAATTAATGTGAGTTAGCTCACTCATTAGGCACCCCAGGCTTTACACTTTATGCTTCCGGCTCGT
ATGTTGTGTGGAATTGTGAGCGGATAACAATTTCACACAGGAAACAGCTATGACCATGATTACG
CCAAGCGCGCAATTAACCCTCACTAAAGGGAACAAAAGCTGGAGCTCCACCGCGGTGGCGGCC
GCTCTAGAACTAGTGGATCCCCCCTAGATGCATGCTCGAGCGGCCGCCAGTGTGATGGATATCT
GCAGAATTCGGCTTCATGGCAATTCCCGGGGATCGTGATTCTGGGTAGAAGATCGGTCTGCATT
GGATGGTGGTAACGCATTTTTTTACACACATTACTTGCCTCGAGCATCAAATGGTGGTTATTCGT
GGATCTATATCACGTGATTTGCTTAAGAATTGTCGTTCATGGTGACACTTTTAGCTTTGACATGA
TTAAGCTCATCTCAATTGATGTTATCTAAAGTCATTTCAACTATCTAAGATGTGGTTGTGATTGG

Comment [um5]: dKL5' target site: **5'GTCAGCGGCCGCATCCCTGCTTCGGCTTCA TGGCAATTCCCG 3'**

GCCATTTTGTGAAAGCCAGTACGCCAGCGTCAATACACTCCCGTCAATTAGTTGCACC**ATG**TCC
ACAAAATCATATACCAGTAGAGCTGAGACTCATGCAAGTCCGGTTGCATCGAAACTTTTACGTT
TAATGGATGAAAAGAAGACCAATTTGTGTGCTTCTCTTGACGTTCGTTCGACTGATGAGCTATT
GAAACTTGTTGAAACGTTGGGTCCATACATTTGCCTTTTGAAAACACACGTTGATATCTTGGAT
GATTTCAGTTATGAGGGTACTGTCGTTCCATTGAAAGCATTGGCAGAGAAATACAAGTTCTTGA
TATTTGAGGACAGAAAATTCGCCGATATCGGTAACACAGTCAAATTACAATATACATCGGGCGT
TTACCGTATCGCAGAATGGTCTGATATCACCAACGCCCACGGGGTTACTGGTGCTGGTATTGTT
GCTGGCTTGAAACAAGGTGCGCAAGAGGTCACCAAAGAACCAAGGGGATTATTGATGCTTGCT
GAATTGTCTTCCAAGGGTTCTCTAGCACACGGTGAATATACTAAGGGTACCGTTGATATTGCAA
AGAGTGATAAAGATTTCGTTATTGGGTTCATTGCTCAGAACGATATGGGAGGAAGAGAAGAAG
GGTTTGATTGGCTAATCATGACCCCAGGTGTAGGTTTAGACGACAAAGGCGATGCATTGGGTCA
GCAGTACAGAACCGTCGACGAAGTTGTAAGTGGTGGATCAGATATCATCATTGTTGGCAGAGG
ACTTTTCGCCAAGGGTAGAGATCCTAAGGTTGAAGGTGAAAGATACAGAAATGCTGGATGGGA
AGCGTACCAAAAGAGAATCAGCGCTCCCCAT**TAA**TTATACAGGAAACTTAATAGAACAAATCA
CATATTTAATCTAATAGCCACCTGCATTGGCACGGTGCAACACTACTTCAACTTCATCTTACAA
AAAGATCACGTGATCTGTTGTATTGAACTGAAAATTTTTTGTTTGCTTCTCTCTCTCTCTCTTTCA
TTATGTGAGATTTAAAAACCAGAAACTACATCATCGAATTCCAGCTGACCACCATGAGCCGAAT
TCCAGCACACTGGCGGCCGTTACTAGTGGATCCGAGCTCGGTACCAAGCTGGGCTGCAGGAATT
CGATATCAAGCTTATCGATGTGATTCTGGGTAGAAGATCGGTCTGCATTGGATGGTGGTAACGC
ATTTTTTTACACACATTACTTGCCTCGAGCATCAAATGGTGGTTATTCGTGGATCTATATCACGT
GATTTGCTTAAGAATTGTCGTTCATGGTGACACGGGCCCGGTACCCAATTCGCCCTATAGTGAG
TCGTATTACGCGCGCTCACTGGCCGTCGTTTTACAACGTCGTGACTGGGAAAACCCTGGCGTTA
CCCAACTTAATCGCCTTGCAGCACATCCCCCTTTCGCCAGCTGGCGTAATAGCGAAGAGGCCCG
CACCGATCGCCCTTCCCAACAGTTGCGCAGCCTGAATGGCGAATGGACGCGCCCTGTAGCGGC
GCATTAAGCGCGGCGGGTGTGGTGGTTACGCGCAGCGTGACCGCTACACTTGCCAGCGCCCTA
GCGCCCGCTCCTTTCGCTTTCTTCCCTTCCTTTCTCGCCACGTTCGCCGGCTTTCCCCGTCAAGCT
CTAAATTCGGGGGCTCCCTTTAGGGTTCCGATTTAGTGCTTTACGGCACCTCGACCCCAAAAAA
CTTGATTAGGGTGATGGTTCACGTAGTGGGCCATCGCCCTGATAGACGGTTTTTCGCCCTTTGAC
GTTGGAGTCCACGTTCTTTAATAGTGGACTCTTGTTCCAAACTGGAACAACACTCAACCCTATCT
CGGTCTATTCTTTTGATTTATAAGGGATTTTGCCGATTTCGGCCTATTGGTTAAAAAATGAGCTG
ATTTAACAAAAATTTAACGCGAATTTTAACAAAATATTAACGTTTACAATTTCCTGATGCGGTA
TTTTCTCCTTACGCATCTGTGCGGTATTTCACACCGCATAGGCAAGTGCACAAACAATACTTAA
ATAAATACTACTCAGTAATAACCTATTTCTTAGCATTTTTGACGAAATTTGCTATTTTGTTAGAG
TCTTTTACACCATTTGTCTCCACACCTCCGCTTACATCAACACCAATAACGCCATTTAATCTAAG
CGCATCACCAACATTTTCTGGCGTCAGTCCACCAGCTAACATAAAATGTAAGCTTTCGGGGCTC

Comment [um6]: 5’-int target site.
5'CTTGACGTTCGTTCGACTGATGAGC 3'

Comment [um7]: 3’-int target site:
5'GAGCAATGAACCCAATAACGAAATC 3'

Comment [um8]: cKL3' target site:
5'CACGGCGCGCCTAGCAGCGGTAACGCCAGGGTTTTCCCAGTCAC 3'

TCTTGCCTTCCAACCCAGTCAGAAATCGAGTTCCAATCCAAAAGTTCACCTGTCCCACCTGCTTC
TGAATCAAACAAGGGAATAAACGAATGAGGTTTCTGTGAAGCTGCACTGAGTAGTATGTTGCA
GTCTTTTGGAAATACGAGTCTTTTAATAACTGGCAAACCGAGGAACTCTTGGTATTCTTGCCAC
GACTCATCTCCATGCAGTTGGACGATATCAATGCCGTAATCATTGACCAGAGCCAAAACATCCT
CCTTAGGTTGATTACGAAACACGCCAACCAAGTATTTCGGAGTGCCTGAACTATTTTTATATGC
TTTTACAAGACTTGAAATTTTCCTTGCAATAACCGGGTCAATTGTTCTCTTTCTATTGGGCACAC
ATATAATACCCAGCAAGTCAGCATCGGAATCTAGAGCACATTCTGCGGCCTCTGTGCTCTGCAA
GCCGCAAACTTTCACCAATGGACCAGAACTACCTGTGAAATTAATAACAGACATACTCCAAGCT
GCCTTTGTGTGCTTAATCACGTATACTCACGTGCTCAATAGTCACCAATGCCCTCCCTCTTGGCC
CTCTCCTTTTCTTTTTTCGACCGAATTAATTCTTAATCGGCAAAAAAAGAAAAGCTCCGGATCAA
GATTGTACGTAAGGTGACAAGCTATTTTTCAATAAAGAATATCTTCCACTACTGCCATCTGGCG
TCATAACTGCAAAGTACACATATATTACGATGCTGTCTATTAAATGCTTCCTATATTATATATAT
AGTAATGTCGTTTATGGTGCACTCTCAGTACAATCTGCTCTGATGCCGCATAGTTAAGCCAGCC
CCGACACCCGCCAACACCCGCTGACGCGCCCTGACGGGCTTGTCTGCTCCCGGCATCCGCTTAC
AGACAAGCTGTGACCGTCTCCGGGAGCTGCATGTGTCAGAGGTTTTCACCGTCATCACCGAAAC
GCGCGA

B. Gene over-expression strategy

For the purposes of the present work, the human orthologue of the selected as the potentially most significant gene in yeast FA-specific response has been over-expressed in *S. cerevisiae* (lacking the native gene) in an attempt to check the applicability of this model organism for nutraceutical development, and simultaneously gain some insight of the affected or altered yeast pathways, and eventually transfer this knowledge to humans. The actual protocols employed for this mutant construction were either standard protocols provided in the used kits or modified protocols from different sources.

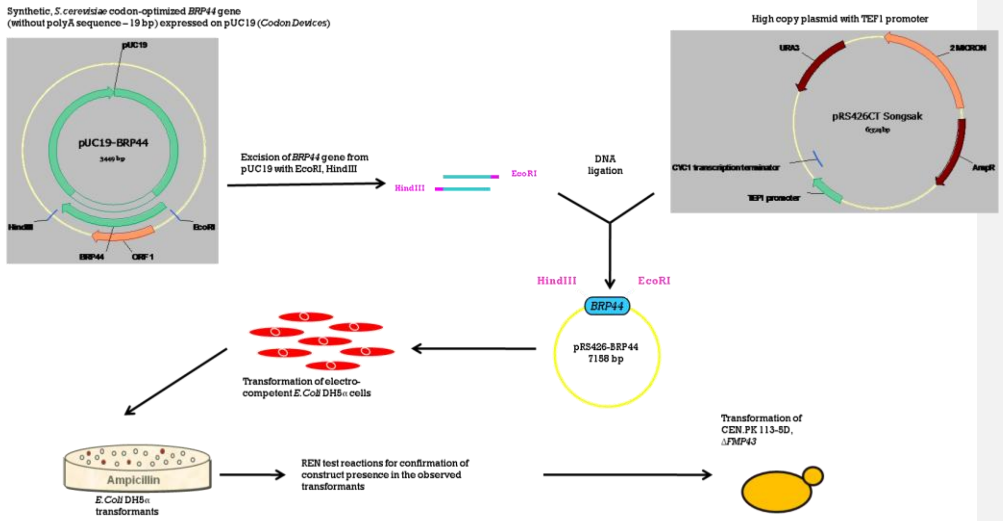

Figure 5. Overview of the steps followed during the attempt to over-express the *FMP43* human orthologue gene (*BRP44*) in *S. cerevisiae*.

Molecular Cloning for *FMP43* gene over-expression

The frame for insertion of the digested *BRP44* fragment (extracted from pUC19 provided by Codon Devices, The Constructive Biology Company™) was a plasmid kindly provided in *E. coli* DH5α cells by Ph.D. student Songsak Wattanachaisaereekul, namely pRS426. This plasmid was constructed as an over-expression vector by inserting the strong constitutive *TEF1* promoter (from the gene encoding translation-elongation factor 1α), and the *CYC1* transcription terminator into pRS426. This original backbone plasmid is a 5726 bp yeast episomal plasmid (YEp)-type shuttle vector with a high copy number of about 20 per cell. The plasmid

contains, among other features, the 2 µm ori and pUC ori for independent episomal replication in *S. cerevisiae* and *E. coli*, respectively, and *URA3* and *ampR* (*bla*, beta-lactamase) genes conferring uracil prototrophy and ampicillin resistance on the host, respectively. In this manner, cloning, amplification, and selection in *E. coli* DH5α cells prior to yeast transformation is enabled.

Figure 6 graphically illustrates the molecular structure of the pRS426-*BRP44* construct.

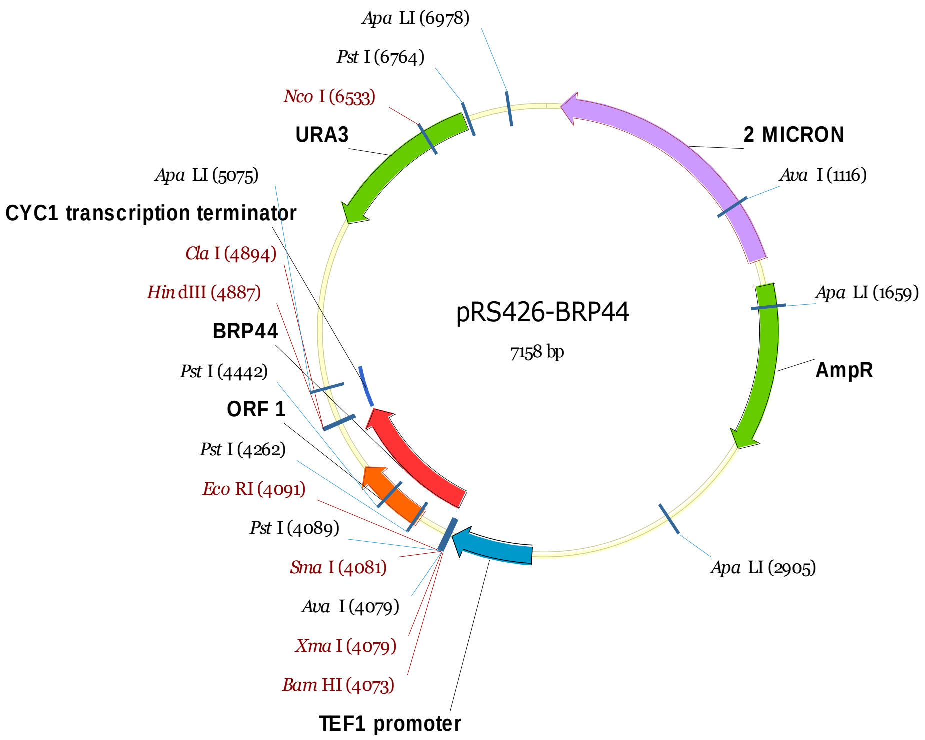

Figure 6. Molecular structure of the pRS426-*BRP44* construct used for yeast transformation during the *BRP44* over-expression mutant construction. The restriction map and the different structural parts of pRS426 together with *BRP44* gene are shown in *blue*, *green*, *purple*, and *red* color, respectively.
