# Supplementary material for: Yeast Biological Networks Unfold the Interplay of Antioxidants, Genome and Phenotype, and Reveal a Novel Regulator of the Oxidative Stress Response
Source: PLoS One. 2010 Oct 25;5(10):e13606. doi: 10.1371/journal.pone.0013606 (PMC2963615; doi:10.1371/journal.pone.0013606)
Supplement: Table S9 — Detailed file with the homology modelling, structure validation, protein-ligand binding site prediction and molecular docking studies of the BRP44 and FMP43 proteins. (0.56 MB PDF) [file pone.0013606.s009.pdf]

1.1. Homology modeling and Structure validation of BRP44:

From PDB Advanced Search Interface, a total of 62 PDB templates were obtained with different E Cut values for amino acid sequence similarity with BRP44. These templates were shortlisted based on resolution, sequence similarity and secondary structure similarity (SOPMA) covering the maximum range of BRP44 sequence. A total of three templates were shortlisted and their secondary structure alignments were shown in Figure 1. Three homology model structures for BRP44 were built using three different combinations of templates.

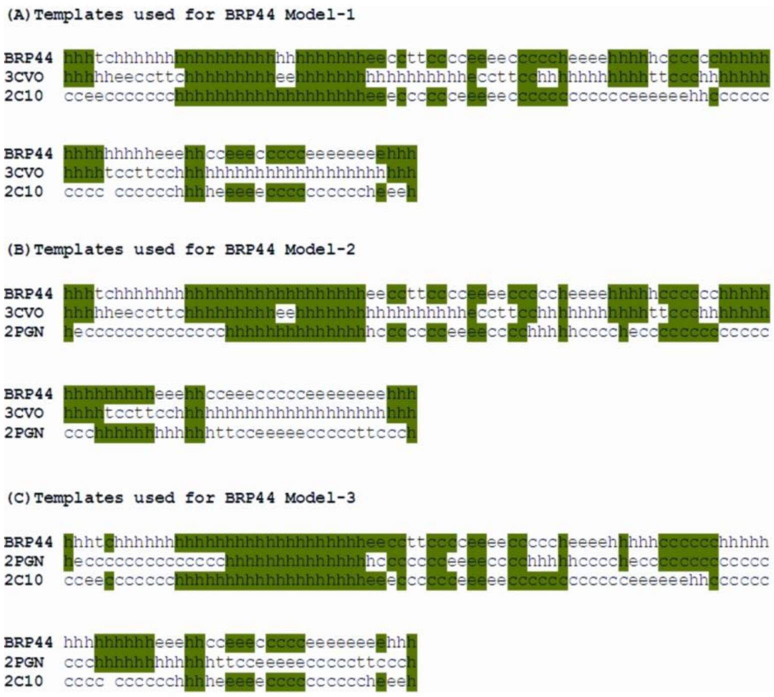

Figure 1: Secondary structure alignment of template groups used for homology modeling of BRP44. The amino acid sequence region from 488-592, 120-224 and 547-650 of 3CV0, 2PGN and 2C10 was used for secondary structure alignments respectively. Template 3CV0 is a Peroxisomal targeting Signal 1 binding domain [15]. Template 2PGN is a FAD and ThDP-dependent Cyclohexane-1,2-dione Hydrolase [16]. Template 2C10 is a Human Semicarbazide-Sensitive Amine Oxidase [17]. The meanings of secondary structure symbols are (h): Alpha helix, (b): Beta bridge, (e): Extended strand, (t): Beta turn, (c): Random coil.

The homology model of BRP44 built using 3CV0 and 2c10 as templates showed good quality factors when compared with other two models. The ProSA Z-Score for BRP44 Model 1, BRP44 Model 2, and BRP44 Model 3 are -1.91, -1.91 and -0.24 respectively. Even though the z-scores of BRP44 Model 1 and BRP44 Model 2 are similar, some distortions were observed in BRP44 Model 2 (not shown here). Ramachandran plot summaries of BRP44 Model-1 and its respective templates obtained from Procheck program were shown in Table 1A. Based on an analysis of 118 structures of resolution of at least 2.0 Angstroms and R-factor no greater than 20%, a good quality model would be expected to have over 90% in the most favoured regions (Procheck). RMS deviations of BRP44 Model-1 and its respective templates were shown in Table 1B. The overall statistics of structure quality factors were shown in Table 1C. The overall model quality *Z*-Score from ProSA-web was shown in Figure 2.

Table 1A: Ramachandran Plot Summary from Procheck

	Most favoured regions	Additionally allowed regions	Generously allowed regions	Disallowed regions
BRP44 (Model 1)	89.9%	10.1%	0.0%	0.0%
3CV0 (488 - 592)	91.6%	8.4%	0.0%	0.0%
2C10 (547 - 650)	92.1%	7.9%	0.0%	0.0%

Table 1B: RMS deviations of BRP44 Model-1 and its respective templates

	Number of close contacts (within 2.2 Å)	RMS deviation for bond angles	RMS deviation for bond lengths
BRP44 (Model 1)	0	1.1 °	0.010 Å
3CV0 (488 - 592)	0	1.3 °	0.013 Å
2C10 (547 - 650)	0	1.3 °	0.010 Å

Table 1C: Structure Quality Factors - overall statistics

Protein structure validation software suite	Verify3D		ProsaII (-ve)		Procheck G-factor (phi / psi only)		Procheck G-factor (all dihedral angles)	
	Raw score	Z-score	Raw score	Z-score	Raw score	Z-score	Raw score	Z-score
BRP44 (Model 1)	0.09	-5.94	-0.45	-4.55	0.29	1.46	0.40	2.37
3CV0 (488 - 592)	0.31	-2.41	0.65	0.00	0.52	2.36	0.34	2.01
2C10 (547 - 650)	0.04	-6.74	-0.10	-3.10	-0.50	-1.65	-0.39	-2.31

Tables 1A, 1B and 1C: Structure validation results of BRP44 Model-1 and its templates. (For explanation of scores and overall statistics, the reader is referred to visit respective references cited in Materials & Methods section)

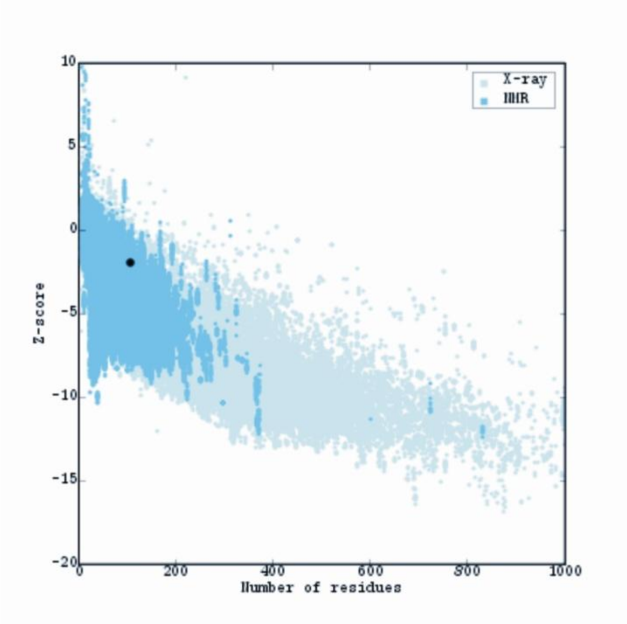

Figure 2: BRP44 (Model 1) ProSA Overall model quality *Z*-Score is -1.91

1.2. Protein-ligand binding site prediction and molecular docking studies –BRP44:

Out of ten protein-ligand binding sites that were predicted using Q-SiteFinder, two potential sites were selected for molecular docking studies. Predicted site-1 contains thirteen amino acids surrounding the cavity with a site volume of 201 Cubic Angstroms. Predicted site-2 contains 10 amino acids surrounding the cavity with a site volume of 115 Cubic Angstroms. Molecular Docking was performed against BRP44 predicted binding site residues using Ferulic acid as ligand. Analysis of best ligand pose energies showed that predicted site-1 has more affinity for ferulic acid. The molecular surface structure of BRP44 with predicted binding site-1 was shown in Figure 3A along with the Ramachandran plot obtained from Procheck program in Figure 3B.

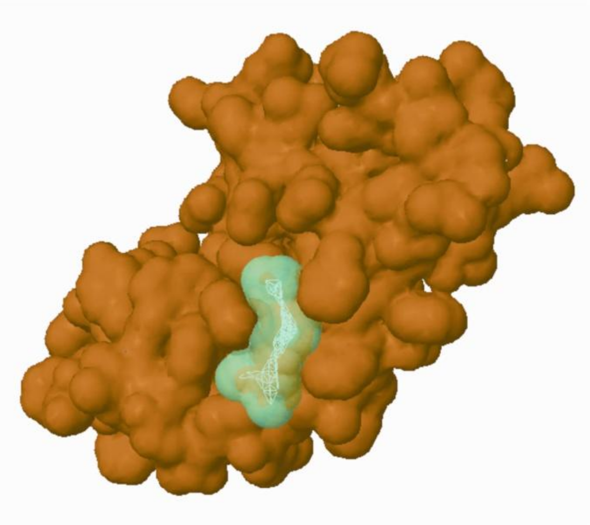

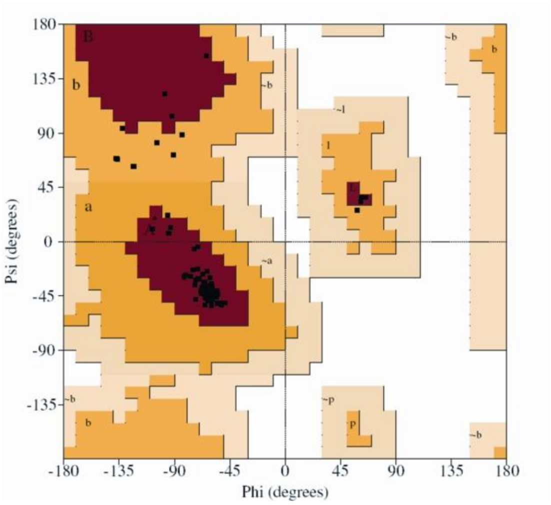

Figure 3A: Molecular surface structure of BRP44 showing predicted binding site-1 in cyan.

Figure 3B: Ramachandran plot of BRP44 structure obtained from Procheck program. None of the amino acid residues are in disallowed regions

According to X-ray crystallography data of Katherine et al (2004), ferulic acid interacts with TYR80, LEU134, THR68 and SER133 in the binding site of Feruloyl esterase (AnFAEA) from *Aspergillus niger*. LEU199 and ILE196 provide a hydrophobic environment in the binding pocket. Protein-ligand binding site prediction and docking studies were performed with AnFAEA (PDB Code: 1uwc). The molecular docking study reveals that ferulic acid indeed has more affinity towards the binding site of BRP44 than the binding site of 1uwc. Comparison of docking energies and binding site residues were shown in Table 2.

Site volume: 213 Cubic Angstroms	AnFAEA - 1uwc	BRP44	Site volume: 201 Cubic Angstroms
	**Residues in binding site**	**Residues in predicted site 1**	
Average of Best Ligand pose energies: -7.48618 kcal/mol	**LEU74, LEU134, LEU199**	**LEU17, LEU52**	Average of Best Ligand pose energies: -8.10896 kcal/mol
	**TYR80**, TYR100	**TYR13**	
	**ILE196**	**ILE47**	
	**PRO161, PRO200**	**PRO46**	
	**HIS97**	**HIS14**	
	**SER133**	GLU21	
	**THR68**, THR78	PHE43	
	ASP77	ALA45, ALA77	
		MET48, MET76	
		LYS49	

Table 2: Comparison of binding sites and molecular docking results of 1uwc and BRP44. The amino acids highlighted in red are the residues that interact with ferulic acid reported in the X-ray crystallography data [18]. Both site volumes and residues in the binding sites are comparable.

According to X-ray crystallography data of Prates et al (2001), ferulic acid interacts with ASP980, TRP982, and ASN1023 in the binding site of Feruloyl esterase module of Xylanase 10B from *Clostridium thermocellum*. LEU958 provide a hydrophobic environment in the binding pocket. Protein-ligand binding site prediction and docking studies were performed with Feruloyl Esterase Module of Xylanase 10B (PDB Code: 1gkl). The molecular docking results from 1gkl and BRP44 reveal that ferulic acid has more affinity towards the binding site of BRP44. Comparison of docking energies and binding site residues were shown in Table 3.

Site volume : 223 Cubic Angstroms	Feruloyl esterase module of Xylanase 10B – 1gkl	BRP44	Site volume : 201 Cubic Angstroms
	**Residues in binding site**	**Residues in predicted site 1**	
Average of Best Ligand pose energies: -6.82768 kcal/mol	**ALA954, ALA1020, ALA1022**	**ALA45, ALA77**	Average of Best Ligand pose energies: -8.10896 kcal/mol
	**LEU958**	**LEU17, LEU52**	
	**MET955**	**MET48, MET76**	
	**ASN1023**	TYR13	
	**TRP982**	ILE47	
	SER978	PRO46	
	**ASP980**	HIS14	
	ILE1019	GLU21	
	GLY957, GLY979	PHE43	
		LYS49	

Table 3: Comparison of binding sites and molecular docking results of 1gkl and BRP44. The amino acids highlighted in red are the residues that interact with ferulic acid reported in the X-ray crystallography data [19].

1.3. Homology modeling and Structure validation of FMP43:

A total of 11 PDB templates were obtained (PDB Advanced Search Interface) with different E Cut values for amino acid sequence similarity with FMP43. These templates were shortlisted based on resolution, sequence similarity and secondary structure similarity (SOPMA) covering the maximum range of FMP43 sequence. Chain B of 2BMX template and Chain W of 2O26 were used for homology modeling of FMP43. 2BMX is a key element of the Mycobacterium tuberculosis defense system against oxidative stress [3]. 2O26 is a Cytokine/signaling Protein [4]. Structure validation was done as described previously and showed in Tables 4A, 4B & 4C.

Table 4A: Ramachandran Plot Summary from Procheck

	Most favoured regions	Additionally allowed regions	Generously allowed regions	Disallowed regions
FMP43	88.3%	11.7%	0.0%	0.0%
2bmxB	87.8%	12.2%	0.0%	0.0%
2o26W	82.2%	17.8%	0.0%	0.0%

Table 4B: RMS deviations of BRP44 Model-1 and its respective templates

	Number of close contacts (within 2.2 Å)	RMS deviation for bond angles	RMS deviation for bond lengths
FMP43	0	2.0 °	0.015 Å
2bmxB	0	1.8 °	0.019 Å
2o26W	0	1.3 °	0.006 Å

Table 4C: Structure Quality Factors - overall statistics

Protein structure validation software suite	Verify3D		ProsaII (-ve)		Procheck G-factor (phi / psi only)		Procheck G-factor (all dihedral angles)	
	Raw score	Z-score	Raw score	Z-score	Raw score	Z-score	Raw score	Z-score
FMP43	0.02	-7.06	-0.91	-6.45	-0.46	-1.49	-0.10	-0.59
2bmxB	0.41	-0.80	0.63	-0.08	-0.28	-0.79	-0.24	-1.42
2o26W	0.41	-0.80	0.45	-0.83	-0.72	-2.52	-0.62	-3.67

Tables 4A, 4B and 4C: Structure validation results of FMP43 and its templates. (For explanation of scores and overall statistics, the reader is referred to visit respective references cited in Materials & Methods section)

1.4. Protein-ligand binding site prediction and molecular docking studies –FMP43:

From the Q-SiteFinder predicted protein-ligand binding sites of FMP43, five potential binding sites were selected based on site volume and molecular docking was performed using Ferulic acid as ligand. Analysis of best ligand pose energies showed that predicted sites-3 & 4 have affinity for ferulic acid. Predicted site-3 contains 15 amino acids surrounding the cavity with a site volume of 228 Cubic Angstroms. Predicted site-4 contains 12 amino acids

surrounding the cavity with a site volume of 228 Cubic Angstroms. The molecular surface structure of FMP43 with predicted binding sites-3 & 4 was shown in Figure 4A along with the Ramachandran plot obtained from Procheck program in Figure 4B.

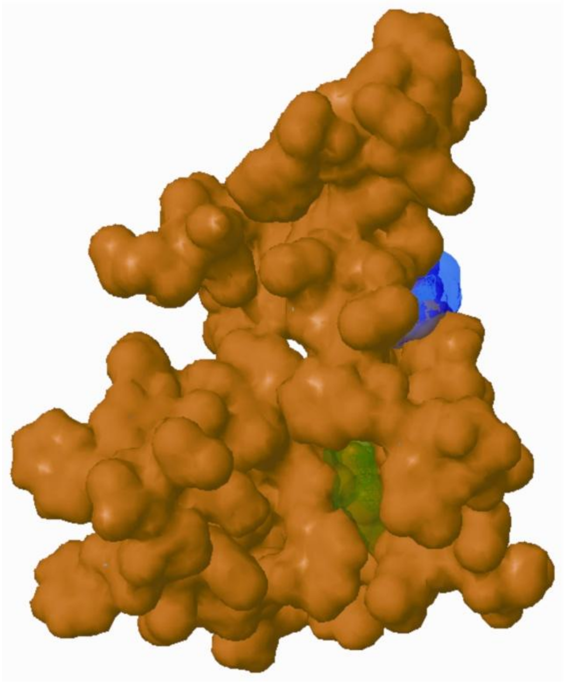

Figure 4A: Molecular surface structure of FMP43 showing predicted binding site-3 in green and predicted binding site-4 in blue.

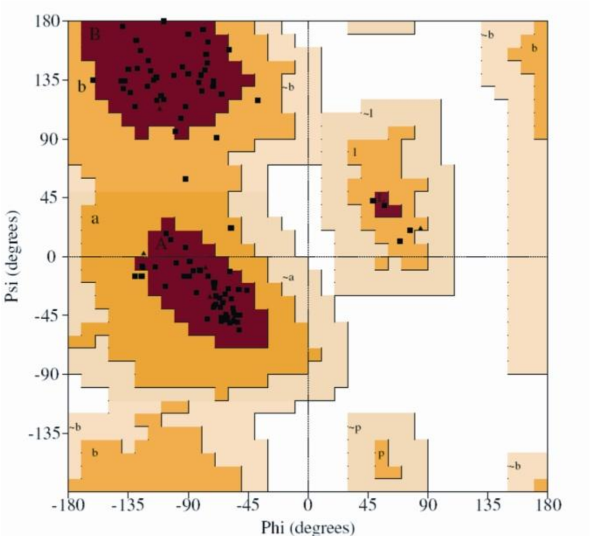

Figure 4B: Ramachandran plot of FMP43 structure obtained from Procheck program. None of the amino acid residues are in disallowed regions

As shown in Tables 5 and 6, Comparison of amino acid residues in binding sites reveals that FMP43 predicted site-3 has some similarities with ferulic acid binding site of 1gkl (FAE domain of Xylanase 10B). Whereas, FMP43 predicted site-4 has some similarities with ferulic acid binding site of 1uwc (AnFAEA).

Site volume : 213 Cubic Angstroms	AnFAEA - 1uwc<br>Residues in binding site		FMP43<br>Residues in predicted site 4	Site volume : 203 Cubic Angstroms
Average of Best Ligand pose	LEU74, LEU134, LEU199		LEU85, LEU93	Average of Best Ligand pose
	THR68, THR78		THR65, THR 88	

Site volume : 223 Cubic Angstroms	Feruloyl esterase module of Xylanase 10B - 1gkl<br>Residues in binding site		FMP43<br>Residues in predicted site 3	Site volume : 228 Cubic Angstroms
Average of Best Ligand pose energies : -6.82768 kcal/mol	**ALA954, ALA1020, ALA1022**		**ALA52, ALA59,ALA61**	Average of Best Ligand pose energies : [illegible] kcal/mol
	**LEU958**		**LEU58, LEU62**	
	**ASN1023**		**ASN15**	
	**TRP982**		**TRP14**	
	SER978		SER50	
	**ASP980**		ASN54	
	[illegible]		[illegible]	
	GLY 957,GLY 979		PHE13	
			GLU47	
			GLN53	
			ARG66	

Table 5: Comparison of binding sites and molecular docking results of 1gkl and FMP43

energies : -7.48618 kcal/mol	**HIS97**		**HIS92**	energies: -7.76721 kcal/mol
	**ILE196**		**ILE63**	
	**TYR80, TYR100**		**TYR76**	
	**SER133**		TRP64	
	ASP77		LYS74	
	PRO161, PRO200		ASN75	
			PHE84	
			ALA89	

Table 6: Comparison of binding sites and molecular docking results of 1uwc and FMP43

2. Conclusion

3D structures of BRP44 and FMP43 were built by homology modeling. Molecular docking of BRP43, FMP43, 1usw and 1gkl was done using ferulic acid as ligand. The best ligand pose energies reveal that ferulic acid can bind in one predicted site of BRP44 and two predicted sites of FMP43. Furthermore, comparison of ferulic acid binding sites in 1usw, 1gkl with BRP44 and FMP43 showed some similarities in amino acid residues surrounding the binding sites. The site volumes and docking energies were also quite comparable. FMP43 showed high structural similarity with 2BMX, a key molecule of the Mycobacterium tuberculosis defense system against oxidative stress.

References

[1] E. M. Katherine, A. Svendsen, S. A. Patkar, K.S. Wilson, Structure of a feruloyl esterase from *Aspergillus niger*, *Acta Cryst.*, D**60** (2004), 878 – 887.

[2] J.A.M. Prates, N. Tarbouriech, S.J. Charnock, C.M.G.A. Fontes, L.M.A. Ferreira, G.J. Davies, The Structure of the Feruloyl Esterase Module of Xylanase 10B from *Clostridium thermocellum* Provides Insights into Substrate Recognition, *Structure*, **9** (2001), 1183–1190.

[3] B.G. Guimarães, H. Souchon, N. Honoré, B. Saint-Joanis, R. Brosch, W. Shepard, S.T. Cole, P.M. Alzari, Structure and mechanism of the alkyl hydroperoxidase AhpC, a key element of the Mycobacterium tuberculosis defense system against oxidative stress, *J Biol Chem.*, **280** (2005), 25735-42.

[4] H. Liu, X. Chen, P.J. Focia, X. He, Structural basis for stem cell factor–KIT signaling and activation of class III receptor tyrosine kinases, *EMBO J.*, **26**(2007), 891-901.
